# Supplementary material for: Few layer 2D pnictogens catalyze the alkylation of soft nucleophiles with esters
Source: Nat Commun. 2019 Jan 31;10:509. doi: 10.1038/s41467-018-08063-3 (PMC6355817; doi:10.1038/s41467-018-08063-3)
Supplement: Supplementary file 1 — Supplementary Information [file 41467_2018_8063_MOESM1_ESM.pdf]

# Supplementary Information

**Few layer 2D pnictogens catalyze the alkylation of soft nucleophiles with esters.**

**Lloret et al.**

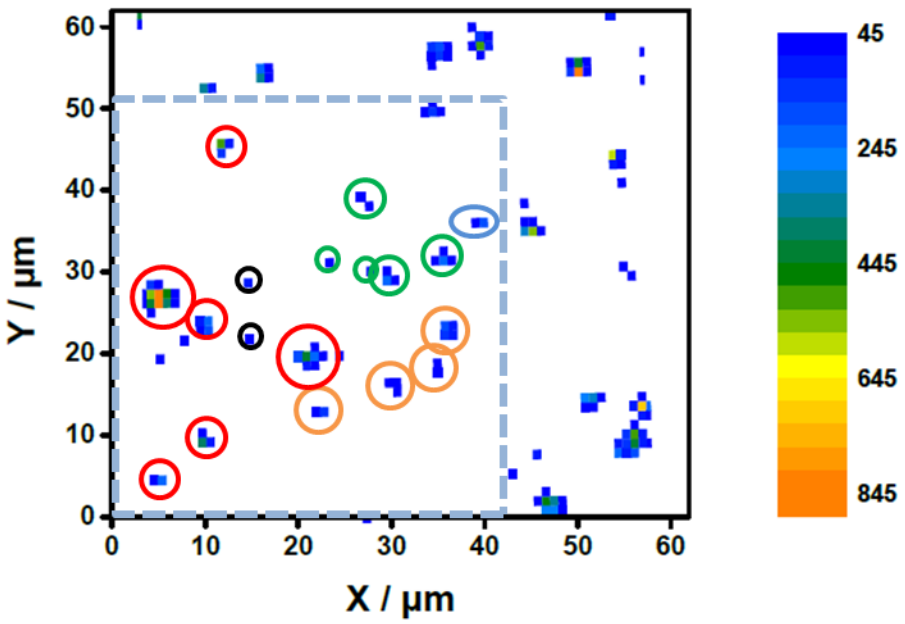


**Supplementary Figure 1.** Raman map of the $A^2_g$ band of BP which correlates to AFM image shown in Supplementary Figure 2. Source data are provided as a Source Data file.

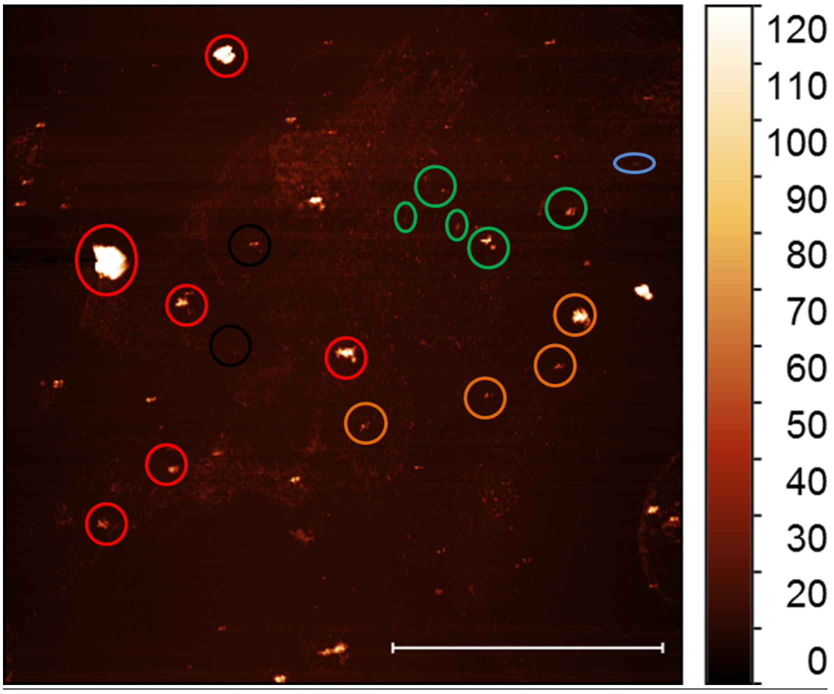


**Supplementary Figure 2.** AFM image corresponding to the Raman mapping shown in Supplementary Figure 1 (scale bar represents 20 µm). Source data are provided as a Source Data file.

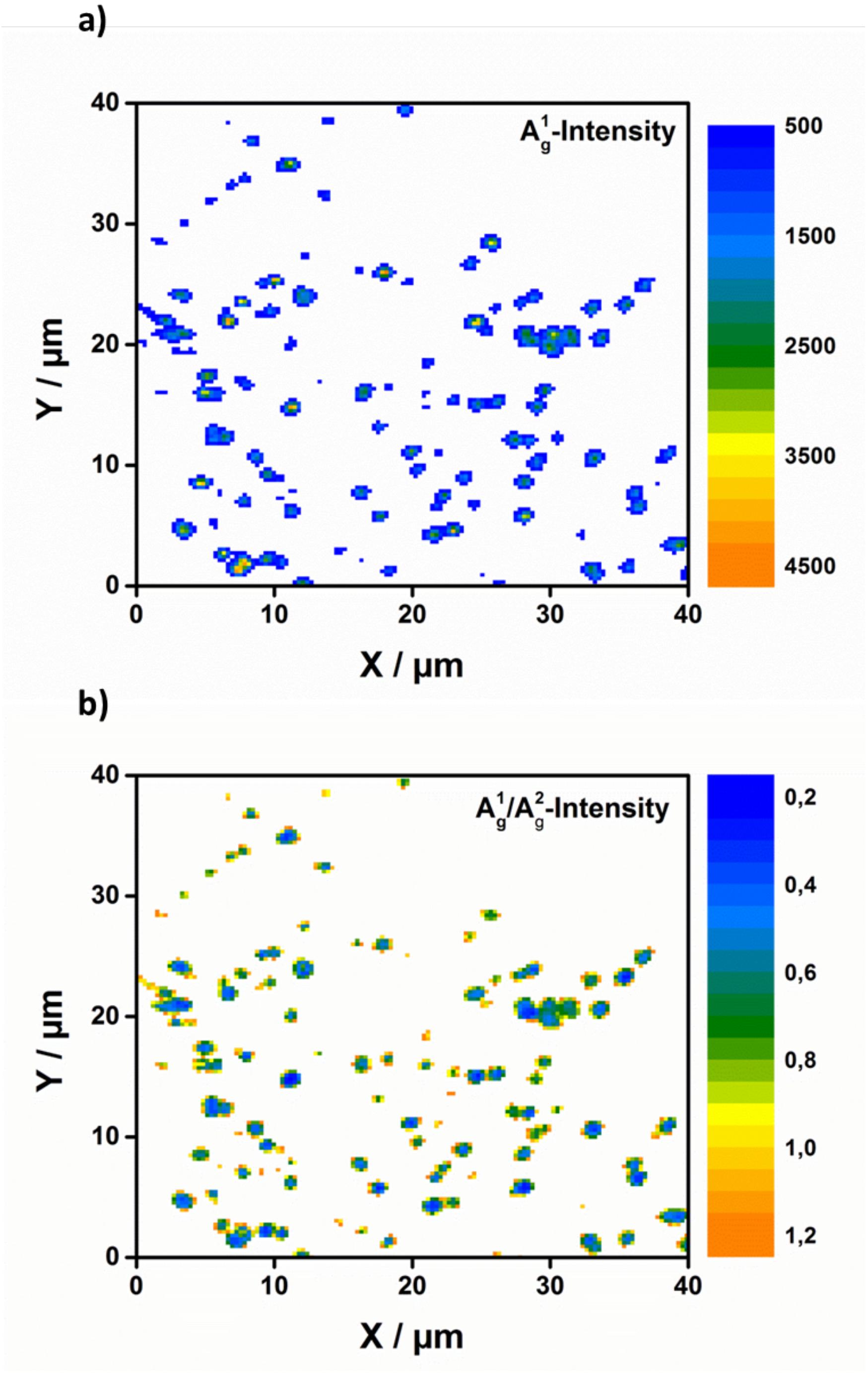


**Supplementary Figure 3.** a) Raman mapping of the $A^1{}_g$ band of BP and b) Raman mapping of $A^1{}_g/A^2{}_g$ ratio. Source data are provided as a Source Data file.

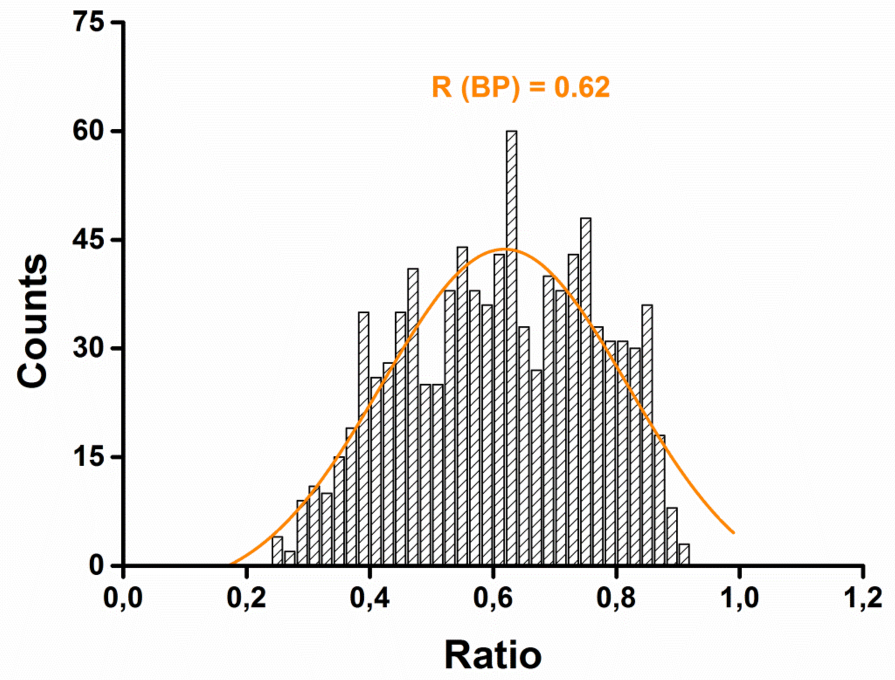


**Supplementary Figure 4**. a) Histogram showing the $A^1_g/A^2_g$ ratio of BP. The orientation of the flakes is random and therefore the ratio varies between values of 0.3 and 0.9. Source data are provided as a Source Data file.

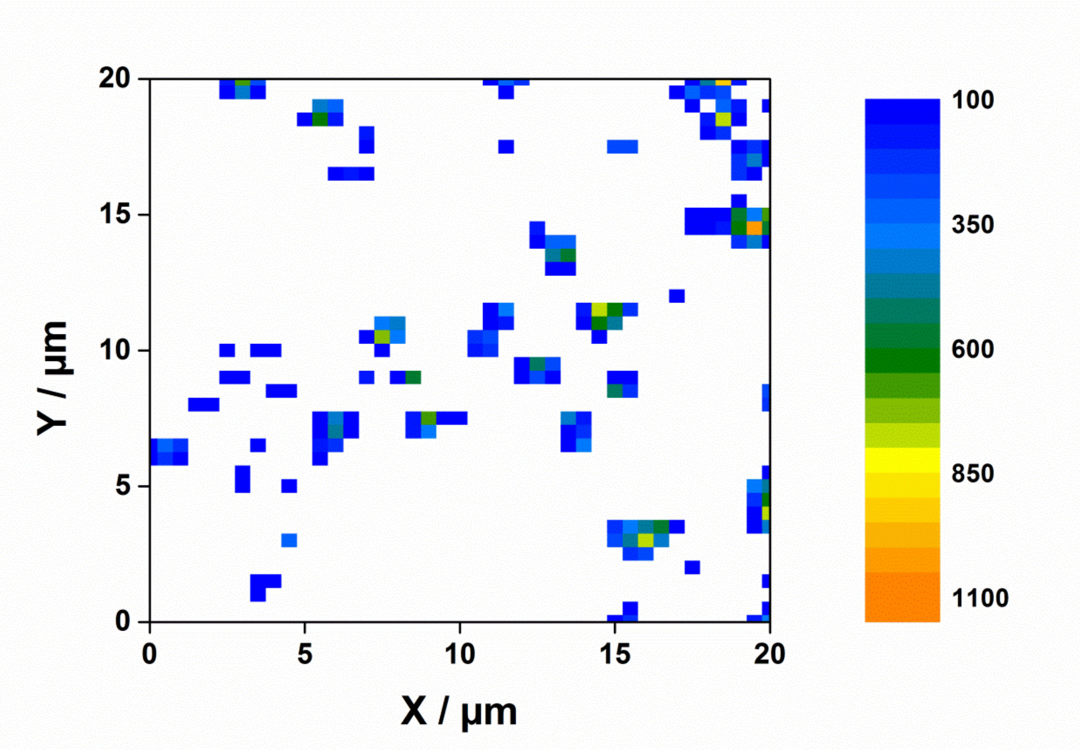


**Supplementary Figure 5.** Raman map of the $A^2_g$ band of BP which correlates to AFM image shown in Supplementary Figure 6. Source data are provided as a Source Data file.

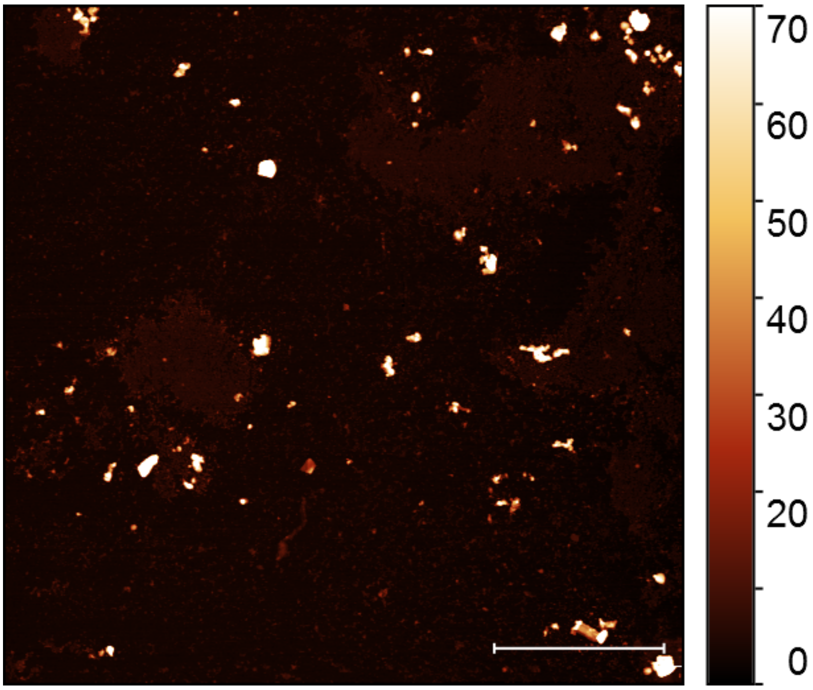


**Supplementary Figure 6.** AFM image corresponding to the Raman mapping shown in Supplementary Figure 5 (scale bar represents 5 µm). Source data are provided as a Source Data file.

a)

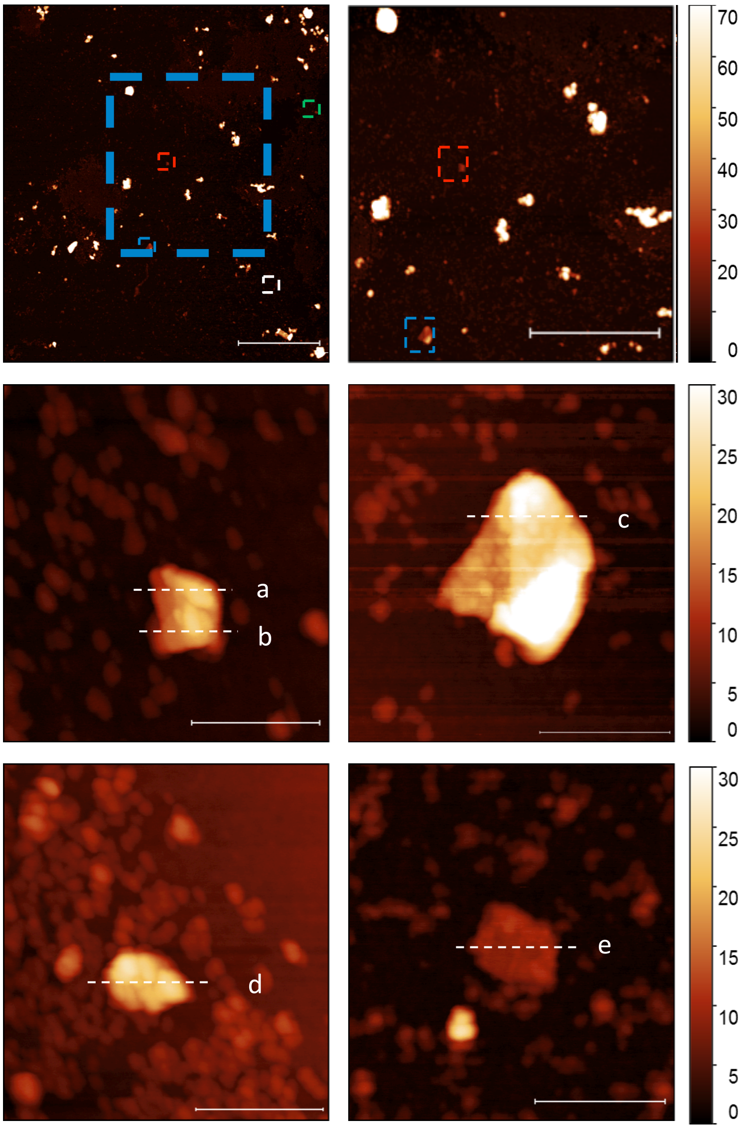


b)

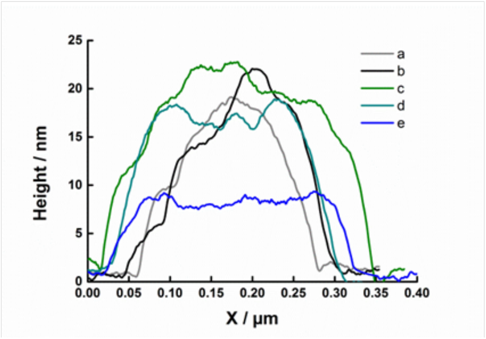


**Supplementary Figure 7.** a) Series of 6 AFM images showing several zooms of areas with thin flakes (scale bars: top right 5 µm, left 2 µm and bottom 200 nm). The first image is the one which was correlated to the Raman map in Supplementary Figure 5 b) Corresponding AFM height profiles across lines a–e.

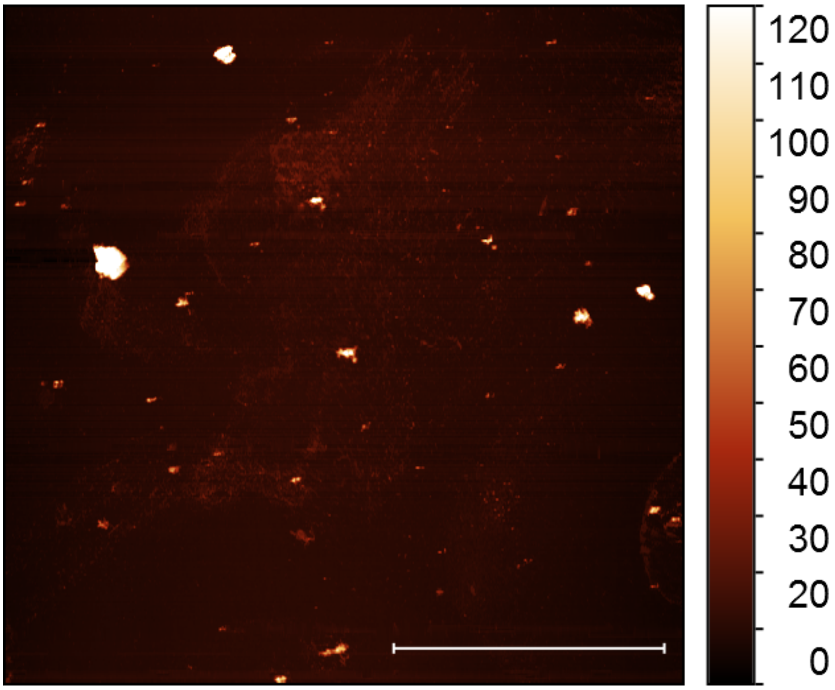


**Supplementary Figure 8**. AFM image of IL deposited on a $Si/SiO_2$ substrate washed with ACN used as reference (scale bar represents 2 μm). Source data are provided as a Source Data file.

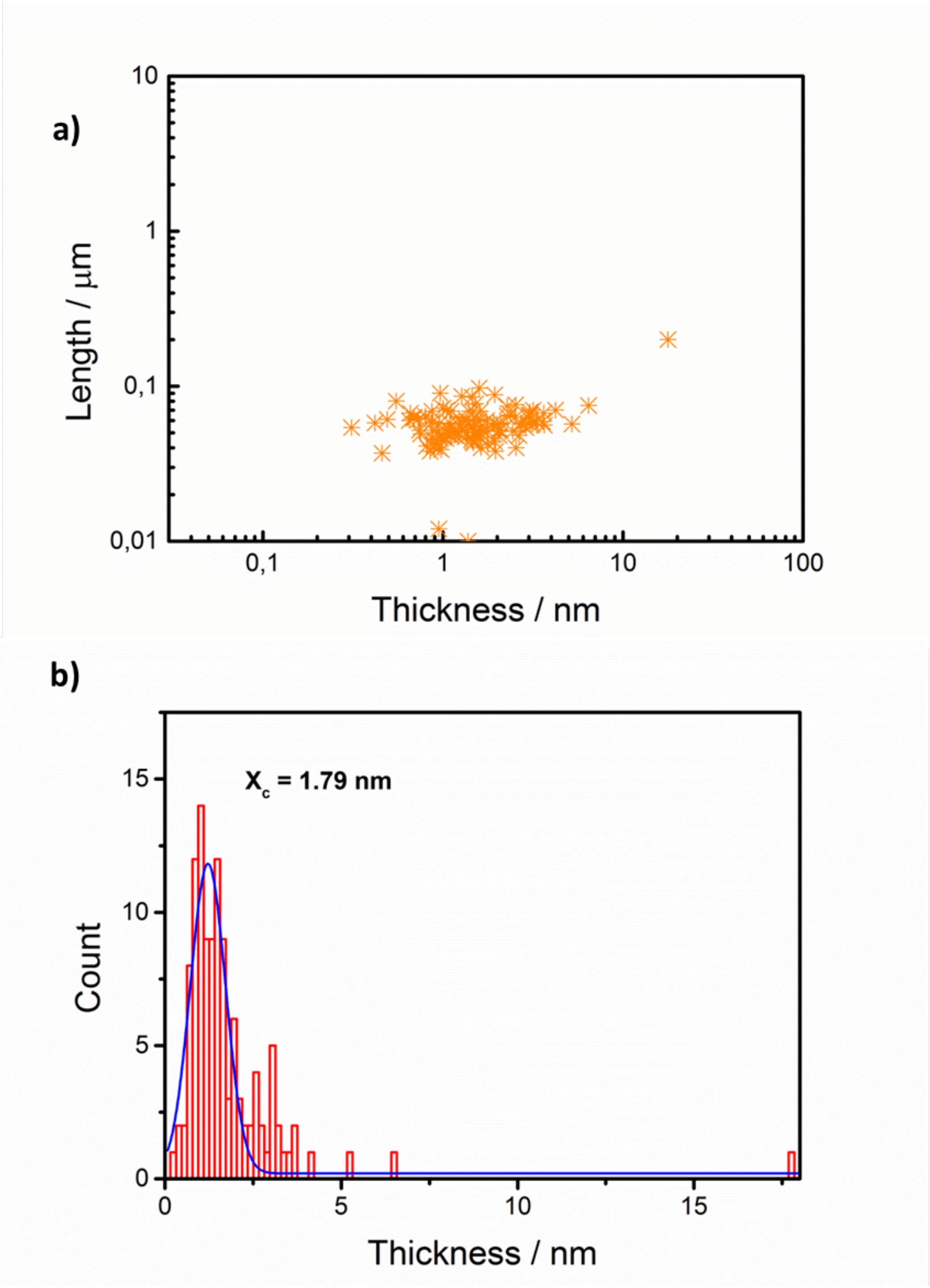


**Supplementary Figure 9**. a) Scatter plot of the AFM values obtained from the length and height of IL deposited on Si/$SiO_2$ substrate (Supplementary Figure 8). b) Represents the histogram of the height values with a mean value of 1.79 nm considering a total amount of 116 replicates. Source data are provided as a Source Data file.

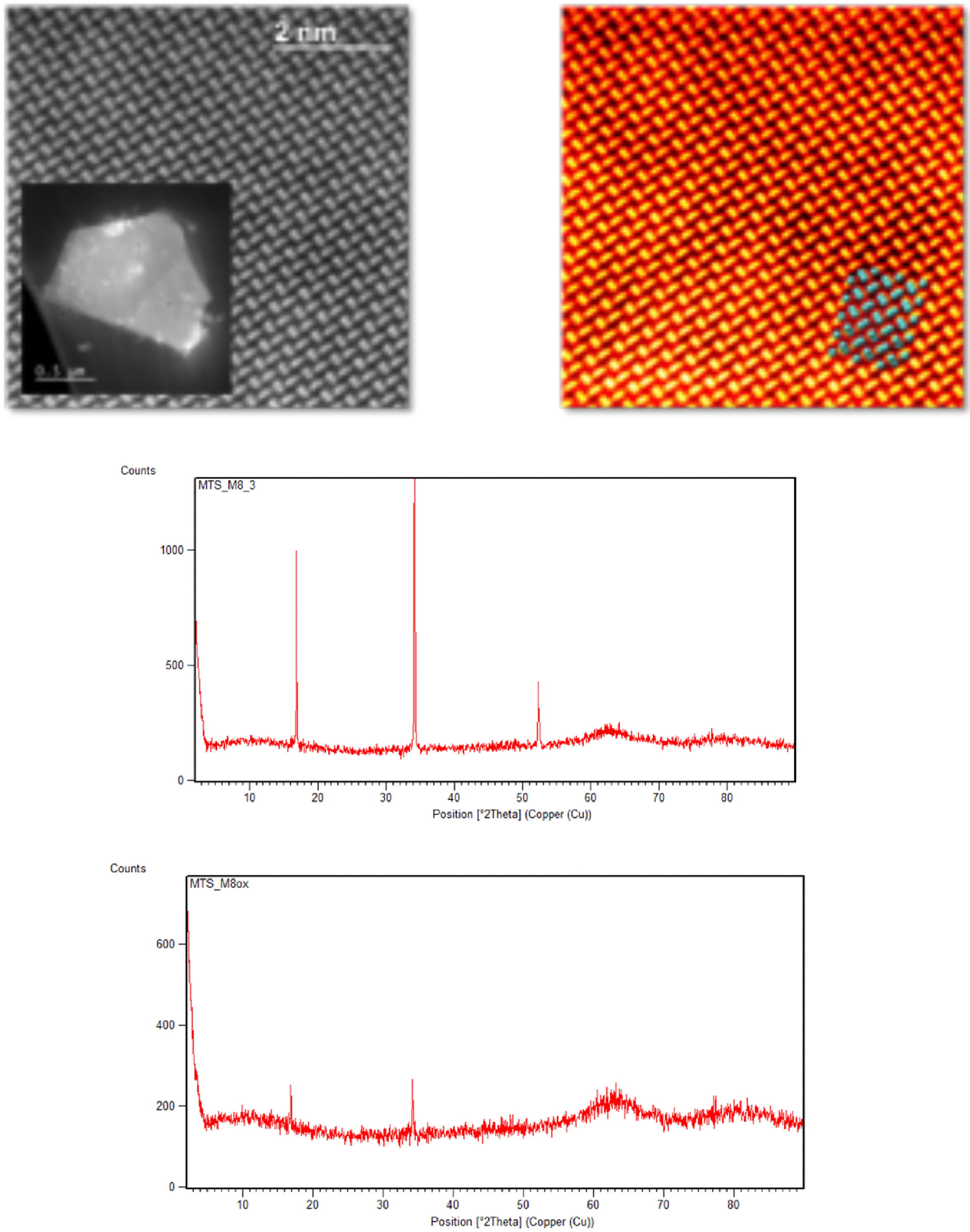


**Supplementary Figure 10.** (Top-left) Atomic resolution HAADF image acquired down the [110] axis, from the edge of a free–standing portion of a flake, including the raw image and a Fourier filtered (FFT) version, in false color (top-right). A sketch of the crystal structure is overlayed. The scale bar represents 2 nm. The inset exhibits a low magnification image of a FL–BP flake, the scale bar is 500 nm. Data acquired at 80 kV. (Middle) XRD of reaggregated FL–BP after washings with tetrahydrofurane solvent, ultracentrifugation and evaporation under inert atmosphere, and the corresponding spectrum after exposure to air for 30 min (bottom).

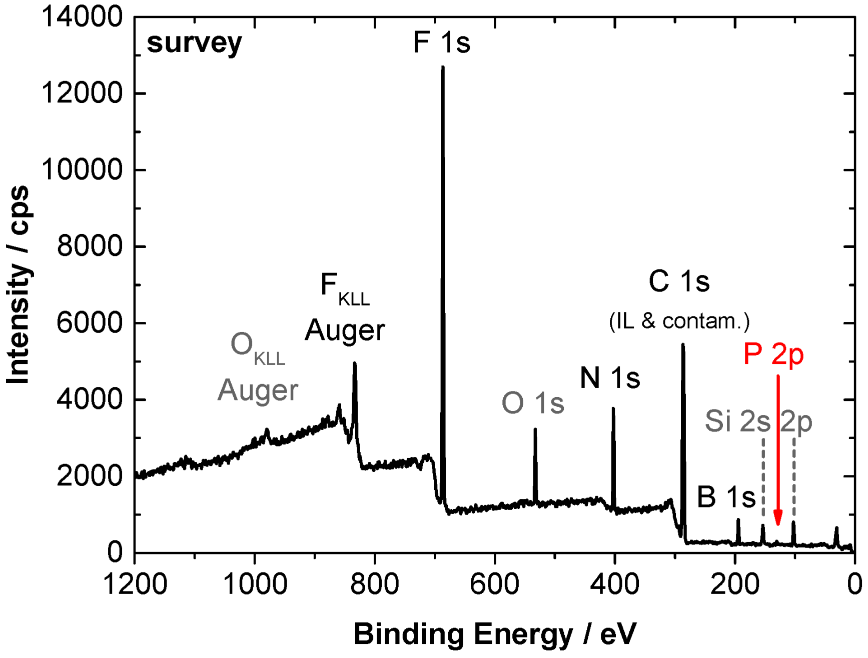


**Supplementary Figure 11.** XPS survey spectrum of FL-BP (Si, O, and C signals originating from an IL contamination are indicated in gray). Source data are provided as a Source Data file.

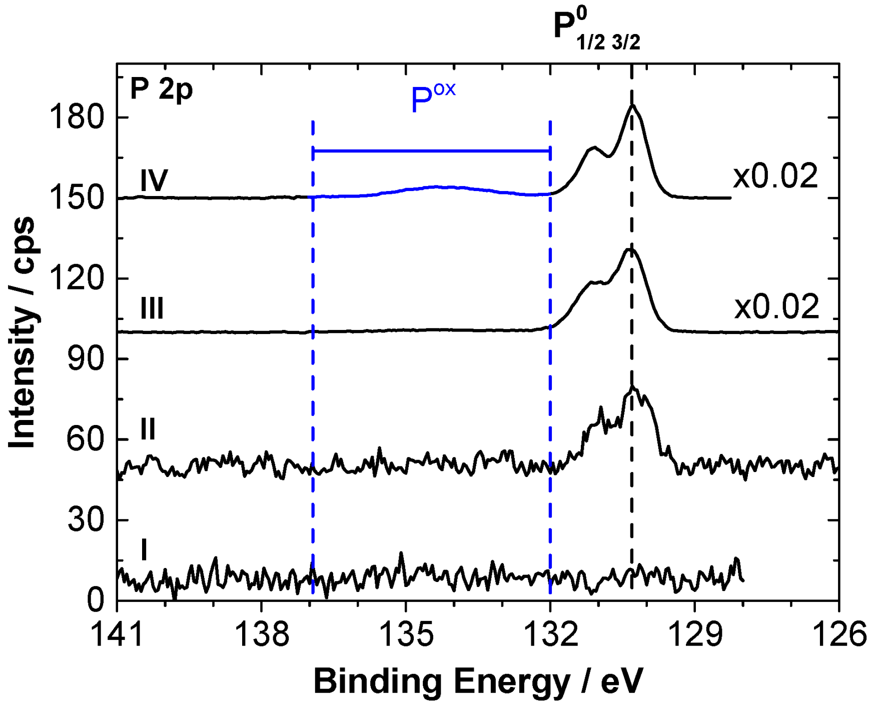


**Supplementary Figure 12.** XPS P 2p region of the neat bmim-$BF_4$ IL (I), the highly-concentrated FL-BP suspension (II) showing only P in oxidation state zero at P $2p_{3/2}$ = 130.2 eV (region for oxidized P species is indicated), after removal of most of the IL by heating in UHV (III), and after having exposed the sample subsequently to environmental conditions for a day, showing the presence of a broad oxide P component around 134 eV (IV). Spectra are offset and re-scaled for sake of clarity. Source data are provided as a Source Data file.

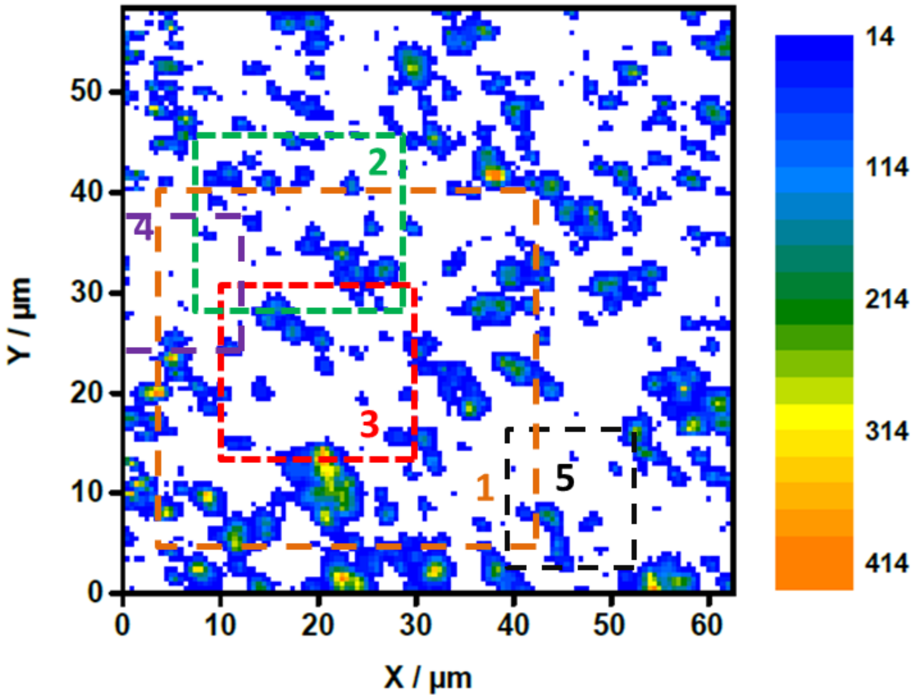


**Supplementary Figure 13.** Raman map of the $A_{1g}$ band of Sb with different squares numbered from 1 to 5 in order to correlate the Sb Raman signals with AFM images. Source data are provided as a Source Data file.

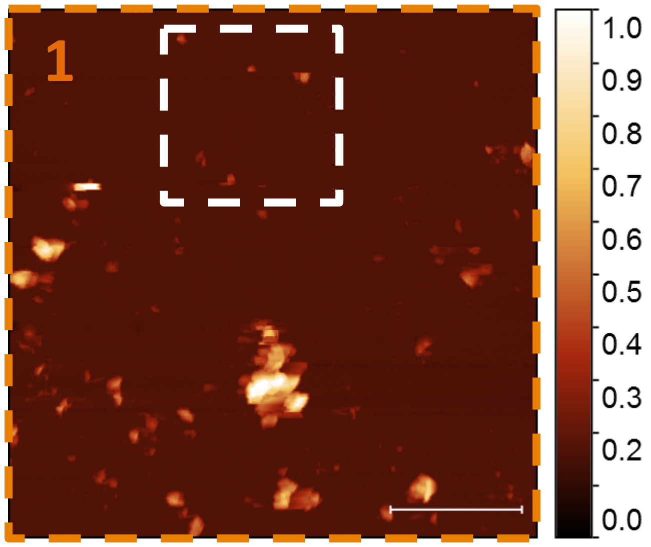


**Supplementary Figure 14.** AFM image showing area 1 (orange) of the corresponding Raman map in Supplementary Figure 13 (scale bar represents10 µm). Source data are provided as a Source Data file.

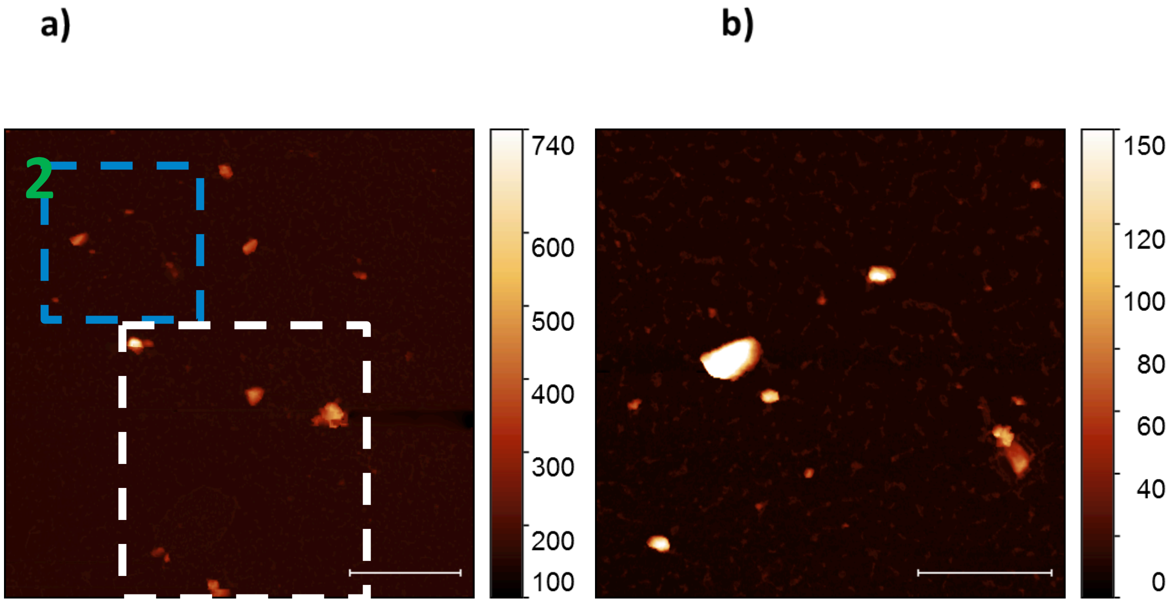


**Supplementary Figure 15.** AFM image showing a Zoom in of area 2 (green) in Supplementary Figure 13, highlighting the white area (a) and a further zoom in b) (scale bars: left 5 µm and right 2 µm). Source data are provided as a Source Data file.

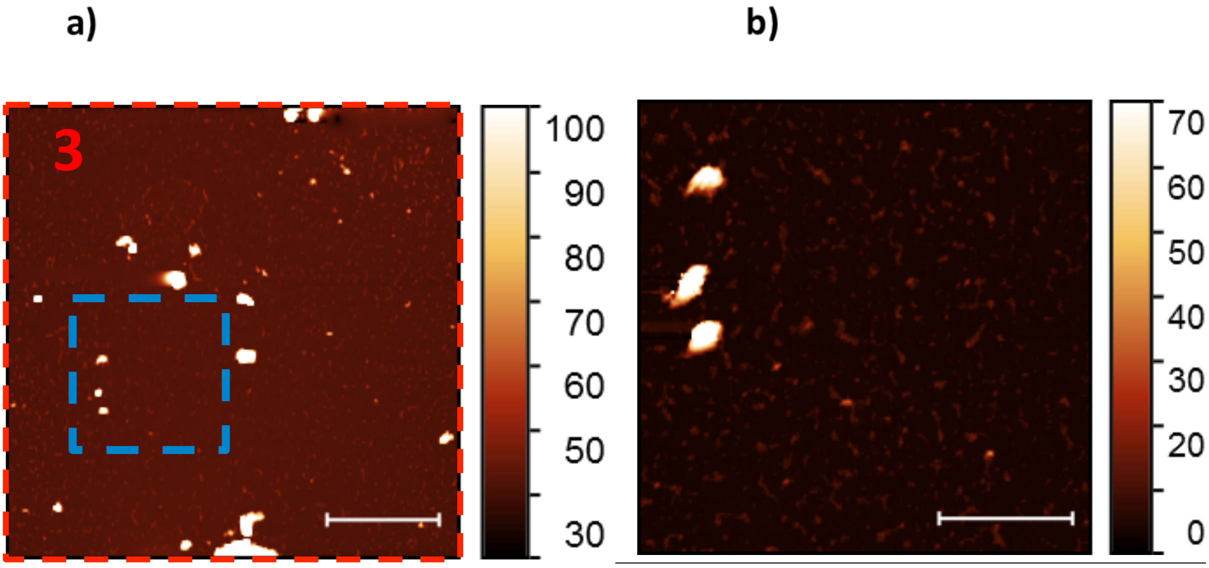


**Supplementary Figure 16.** a) AFM image showing the Zoom in of area 3 (red) in Supplementary Figure 13 and b) the further zoom in of the blue area (scale bars: left 5 µm and right 2 µm). Source data are provided as a Source Data file.

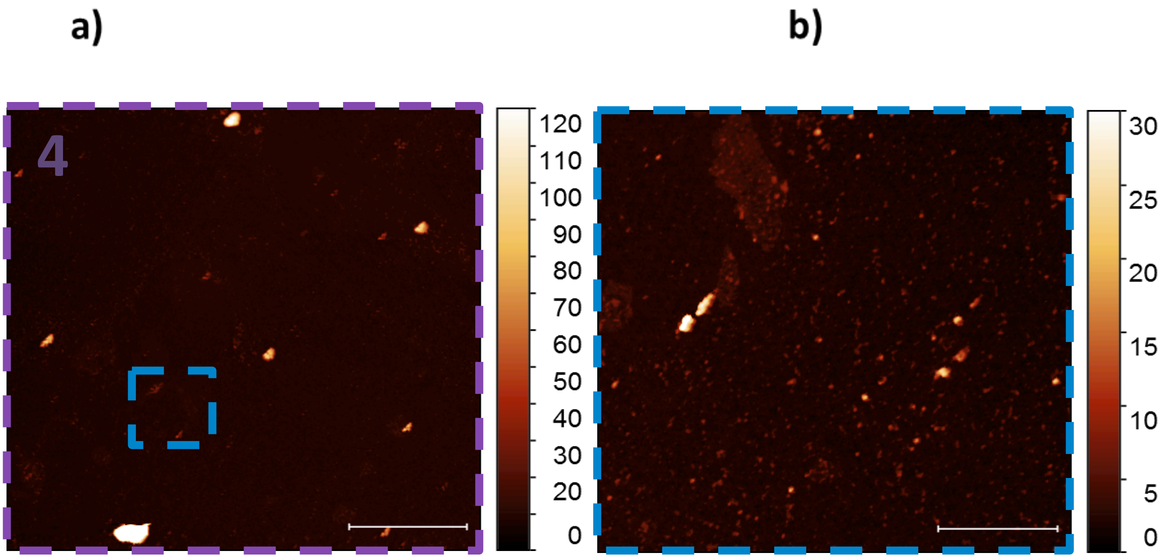


**Supplementary Figure 17.** a) AFM image showing the Zoom in of area 4 (violet) in Supplementary Figure 13 and b) the further zoom in of the blue area (scale bars: left 5 µm and right 1 µm). Source data are provided as a Source Data file.

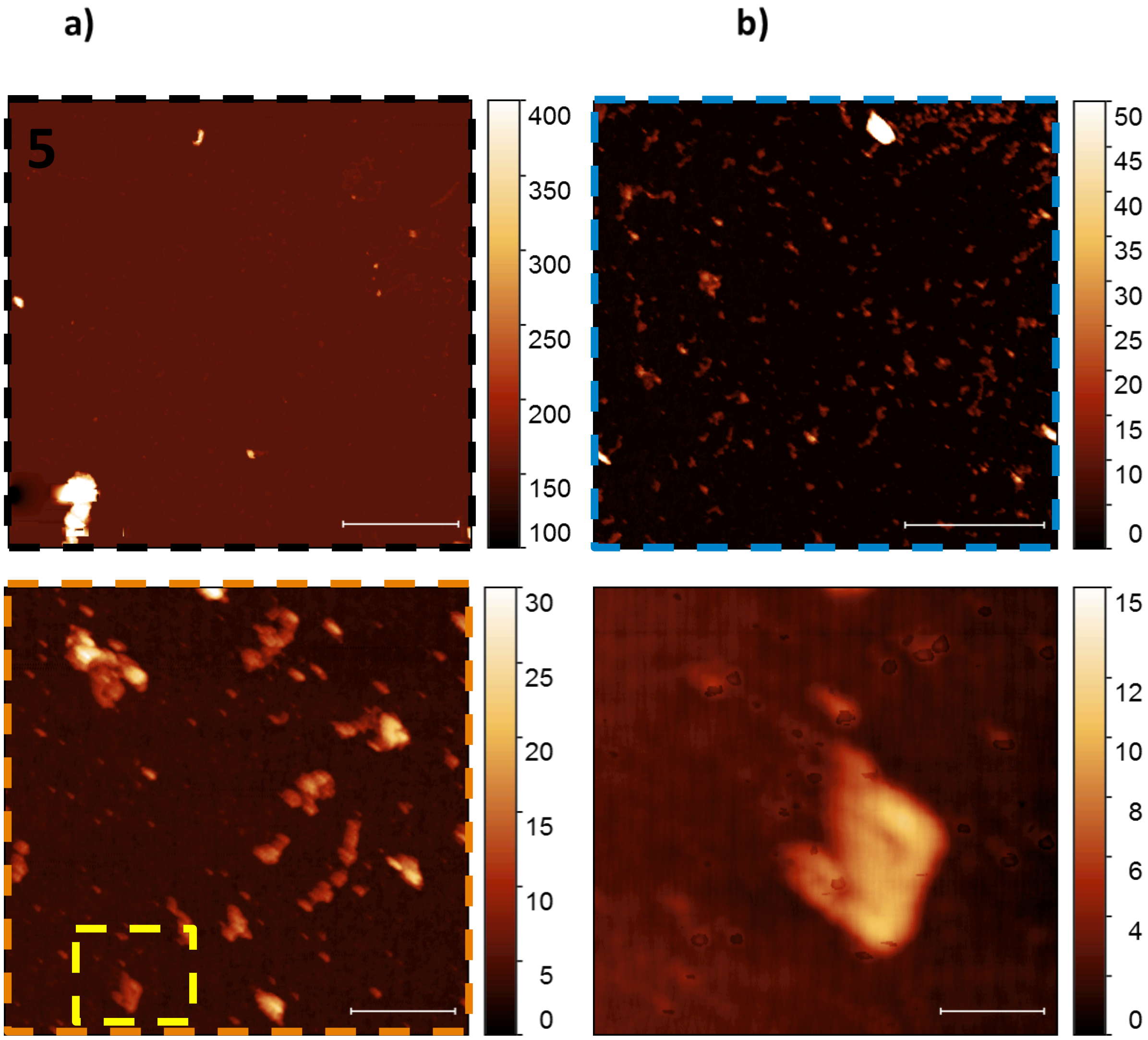


**Supplementary Figure 18.** a) AFM image showing the zoom in of area 5 (black, left; scale bar represents 5 µm) in Supplementary Figure 13 and further zoom in of the selected areas (b–d, scale bars: right 2 µm, bottom left 500 nm and right 100 nm).

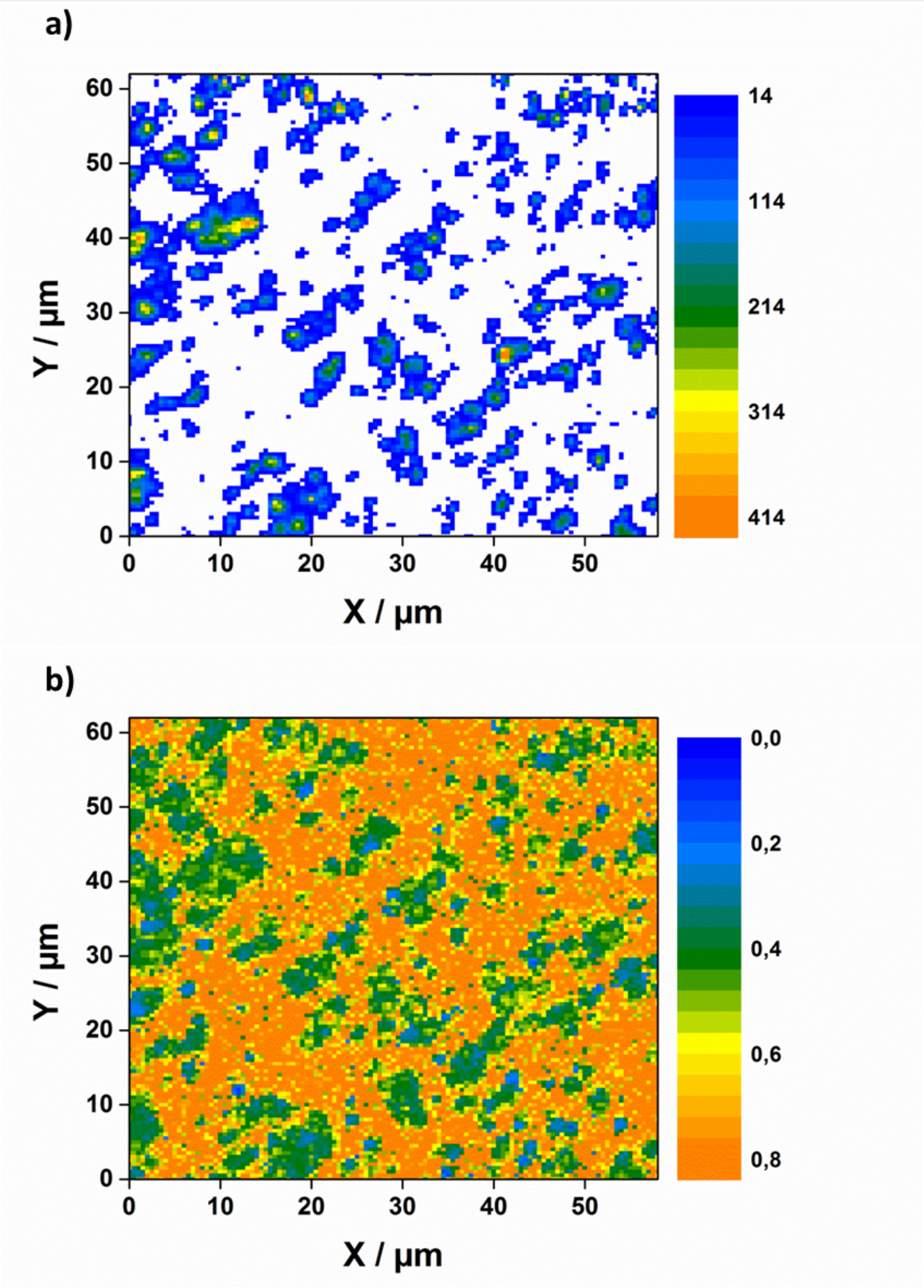


**Supplementary Figure 19**. a) Raman mapping of the $A^1_g$ band of Sb and b) Raman mapping of the Ratio (R) between the $A^1_g$ and $E_g$ Raman mode of Sb. Source data are provided as a Source Data file.

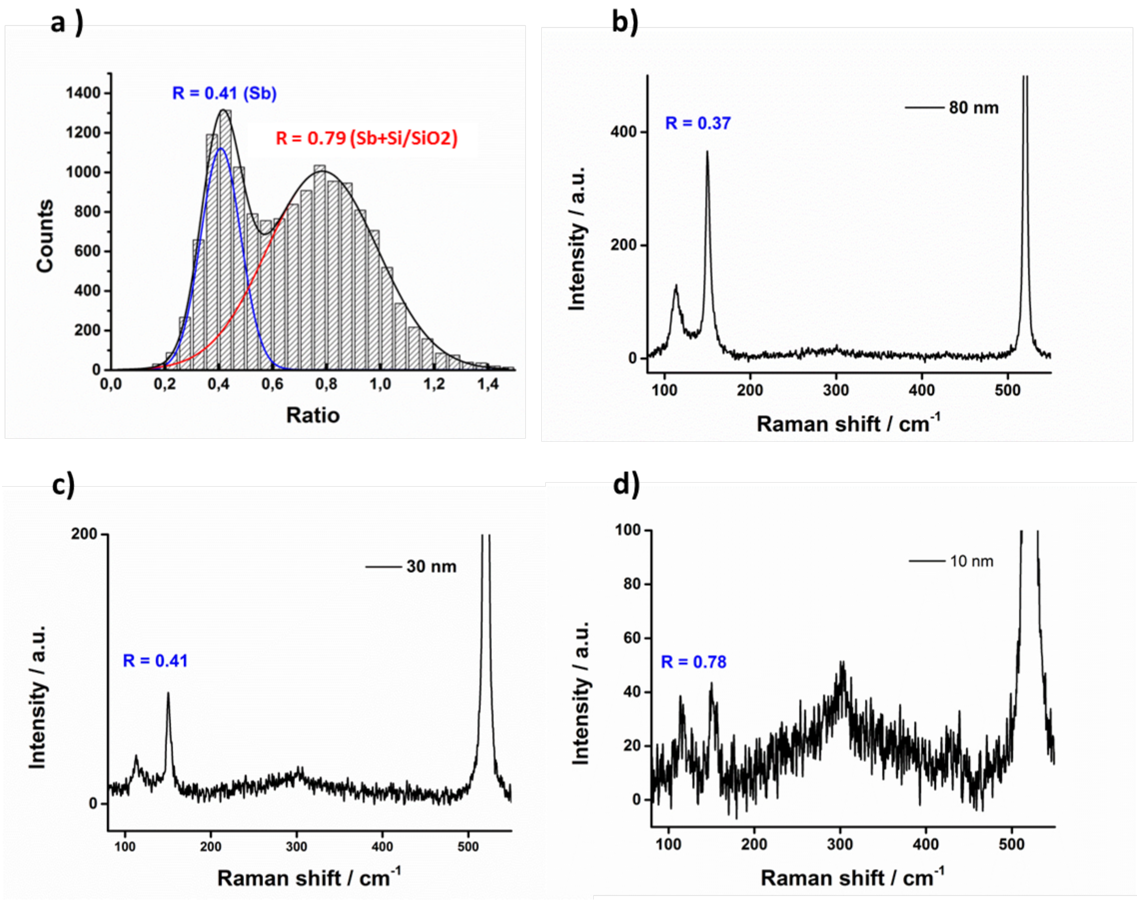


**Supplementary Figure 20.** a) Histogram showing the Ratio $A^1{}_g/E_g$ of Sb (blue R=0.41). Raman single point spectra of different Sb flakes with thicknesses from 80 nm to 10 nm (b–d) highlighting the ratio. Note that with decreasing thickness the Ratio increases but at a certain point cannot be separated anymore from the Si/$SiO_2$ substrate because of the low counts. Source data are provided as a Source Data file.

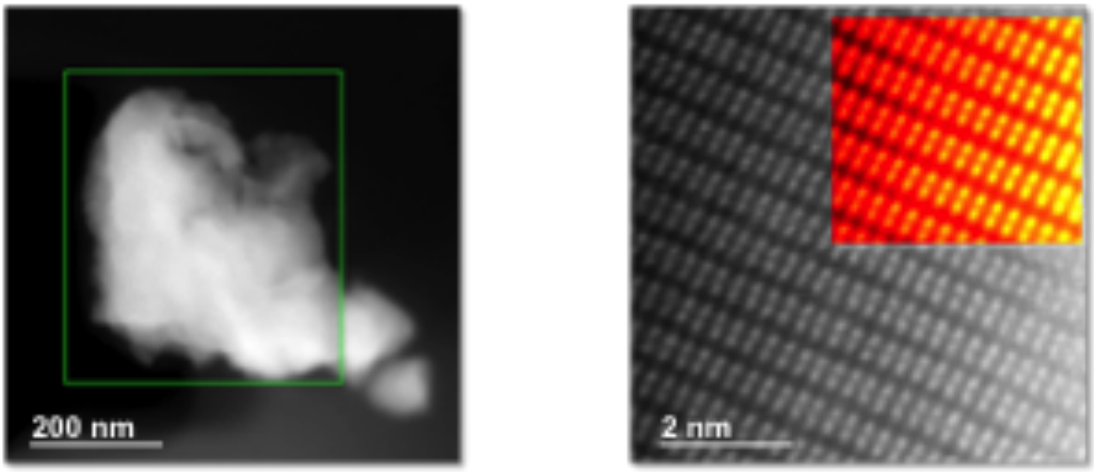


**Supplementary Figure 21.** (left) Low magnification HAADF STEM image of a FL–Sb flake. The scale bar represents 200 nm. Compositional maps derived from EEL spectrum images of the flake shown in Figure 2 of the main text has been acquired from the area highlighted with a green rectangle. (right) Atomic resolution HAADF image acquired on the edge of a free–standing portion of a flake, near the edge, along with a Fourier filtered (FFT) image in the inset, acquired down the [210] orientation. The scale bar is 2 nm. Data acquired at 80 kV.

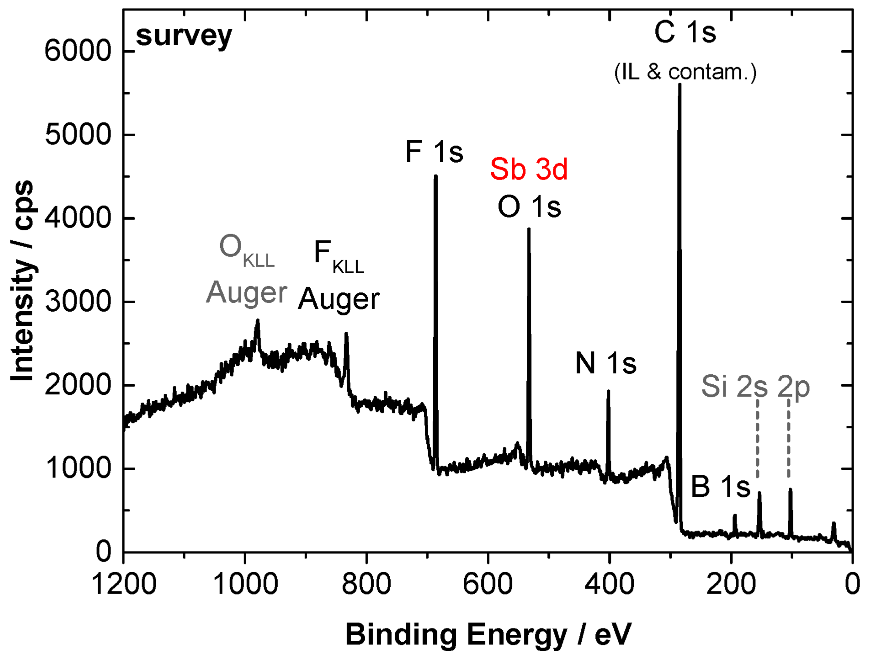


**Supplementary Figure 22.** XPS survey spectrum of FL-Sb. Source data are provided as a Source Data file.

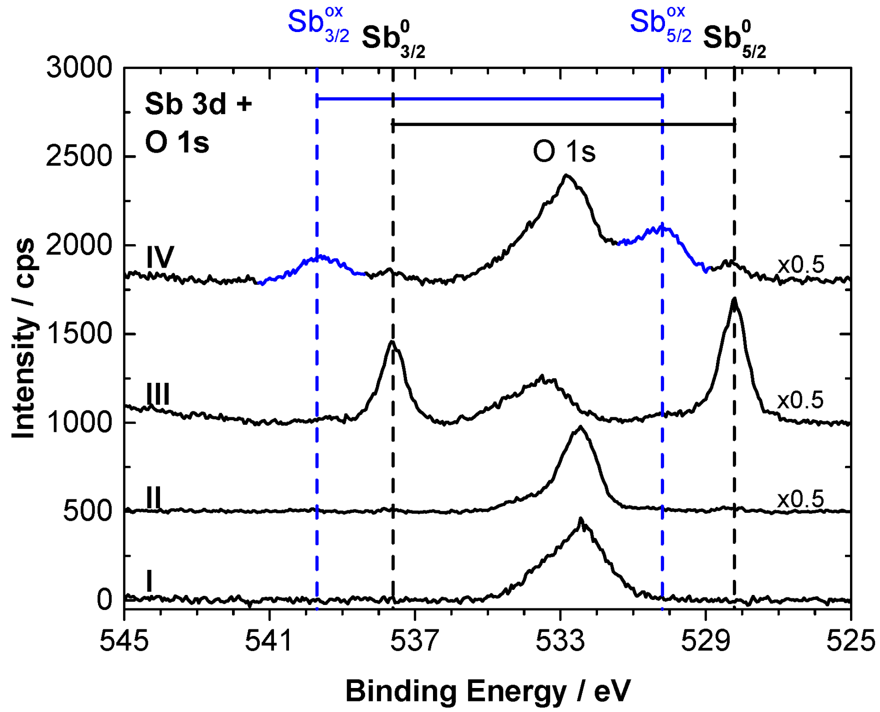


**Supplementary Figure 23.** XPS Sb 3d and O 1s region of the neat bmim-$BF_4$ IL (I) showing oxygen signals from the IL surface contamination layer, of the highly-concentrated FL-Sb suspension (II) showing small signals of non-oxidized (Sb $3d_{5/2}$ at 528.2 eV) and minor contributions from oxidized (530.3 eV) antimony next to the oxygen contamination, after removal of most of the IL by heating in UHV (III), and after submitting the sample to environmental conditions for a day, showing a drastic decrease in Sb(0) and concomitant

increase of the oxidized Sb species. Spectra are offset and re-scaled for sake of clarity. Source data are provided as a Source Data file.

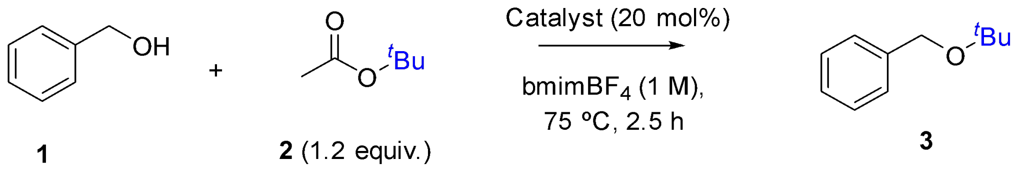


**1** **2** (1.2 equiv.) **3**

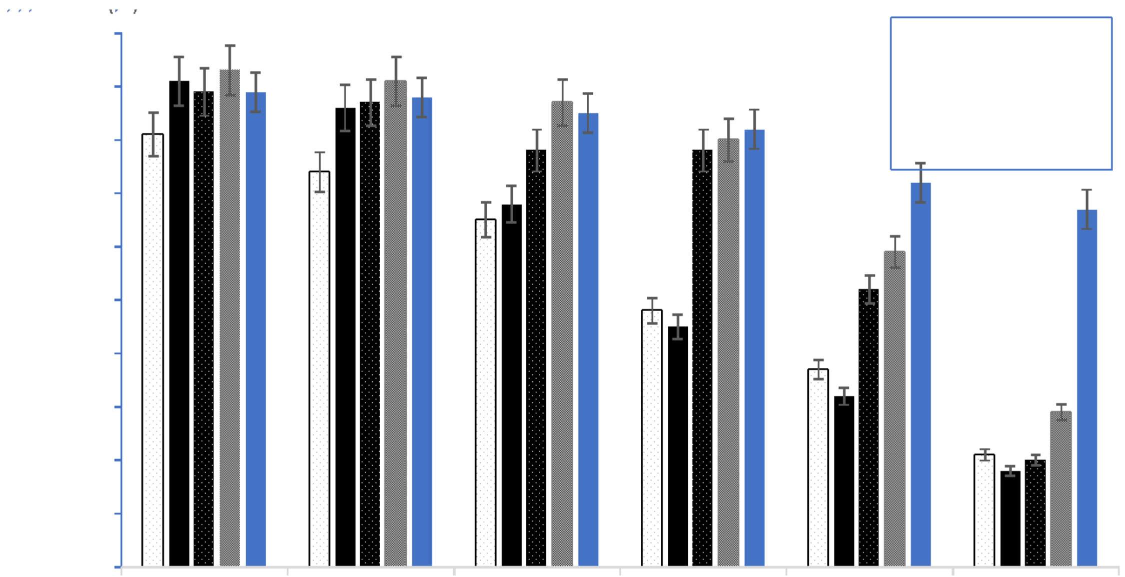

**Supplementary Figure 24.** Reuse tests. The FL-BP catalyst lifetime was prolonged with a sonication treatment after each use (**5**). Error bars account for 5% uncertainty. Source data are provided as a Source Data file.

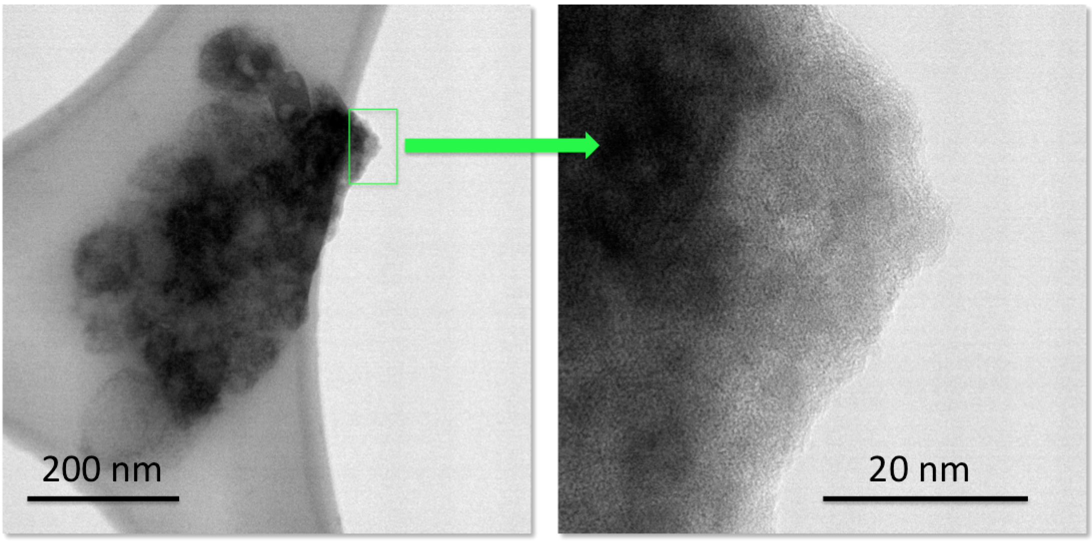


**Supplementary Figure 25.** Low magnification (left) and high magnification (right) annular bright field images of a $N_2$–cycled FL–Sb sample exhibiting significant edge amorphization.

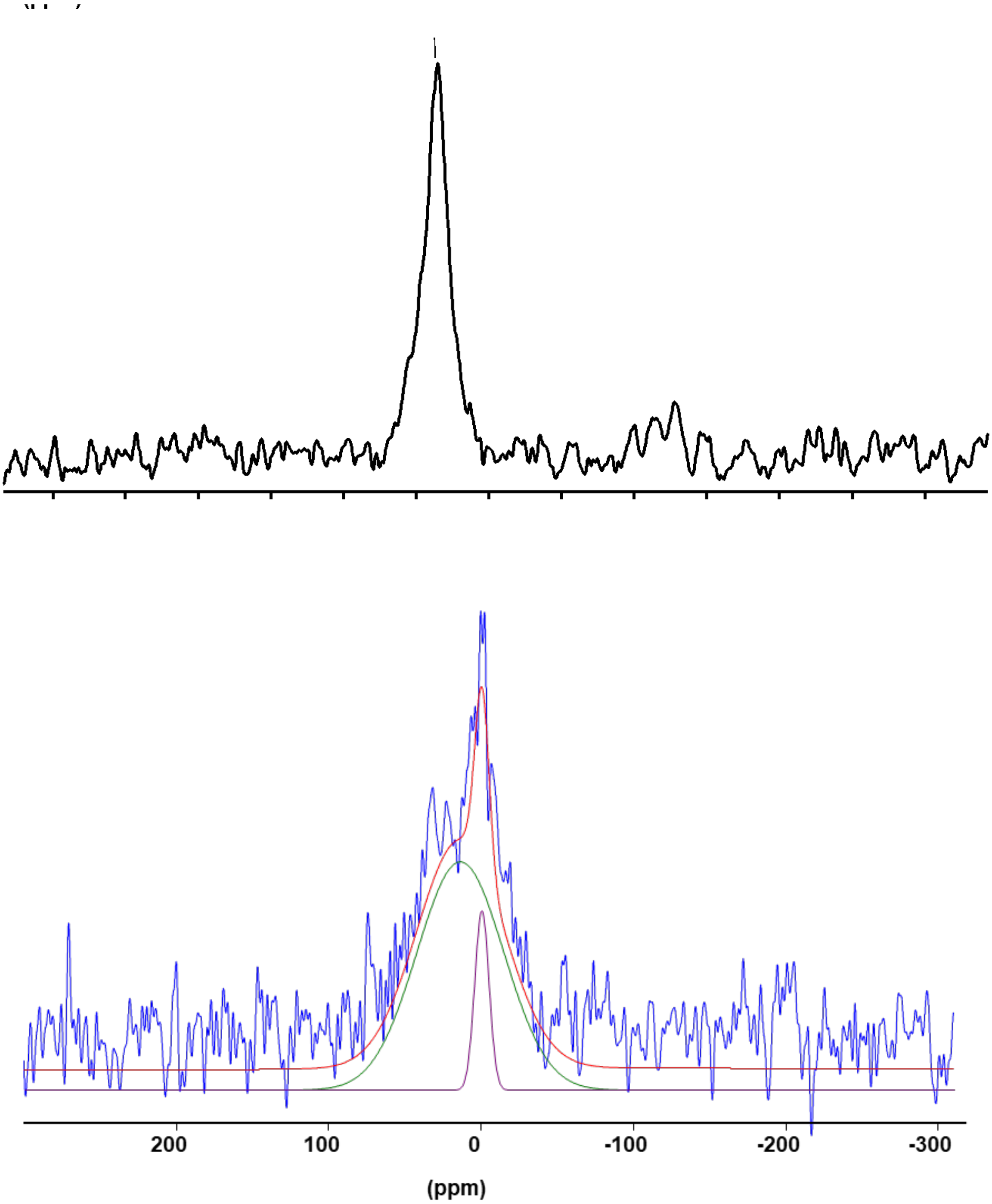


**Supplementary Figure 26.** Top: $^{31}$P MAS NMR spectrum of FL–BP in the presence of ten–fold amounts of **1** and **2**. Bottom: Deconvoluted solid state NMR of FL–BP in statics, without spinning. Blue line shows the raw whole spectrum, red line the sum of components, green line P in zero oxidation state and purple line oxidized P, which accounts for 8% out of the total.

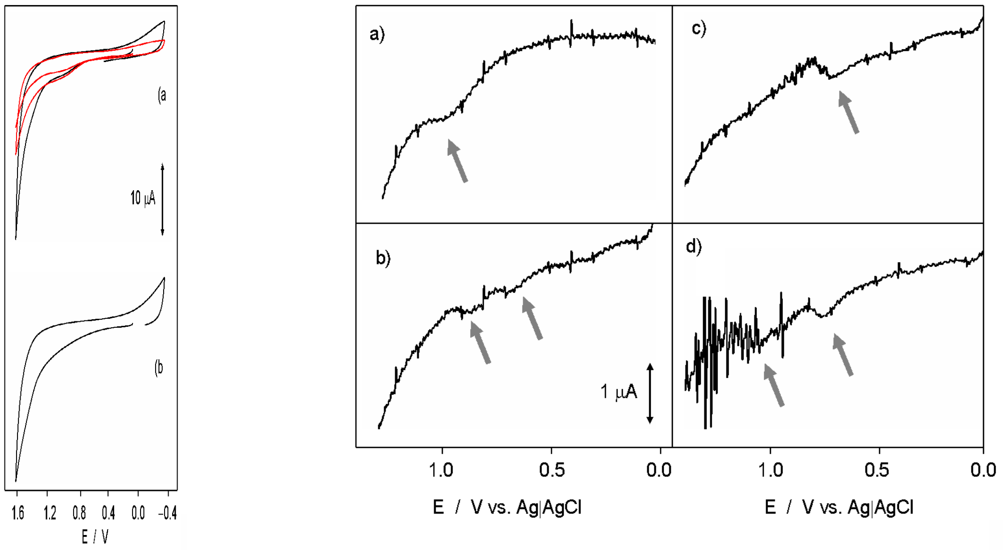


**Supplementary Figure 27** Left: Cyclic voltammograms at glassy carbon electrode of suspensions of a) FL–BP and b) Fl–Sb (black lines) in air-saturated $bmimBF_4$. Red line in a) corresponds to the blank voltammogram in $bmimBF_4$ under the same experimental conditions. Potential scan rate 50 mV $s^{-1}$; potentials relative to Pt pseudo-reference electrode. Right: Linear potential scan voltammograms, after semi-derivative convolution, of a,b) FL-Sb and c,d) FL-BP nanoparticulate deposits on glassy carbon electrode immersed into 0.10 M potassium phosphate aqueous buffer at pH 7.0 extracted from IL solutions a,c) before and b,d) after addition of the BrOAc plus *t*-BuOAc reactants. Potential scan initiated at 0.0 V in the positive direction. The arrows indicate signals of agglomeration/fractioning of the particles.

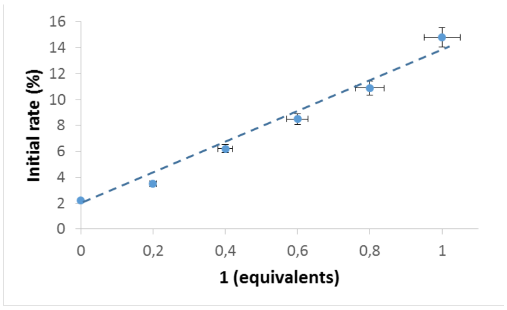

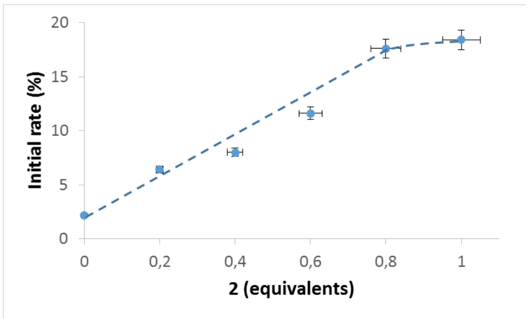

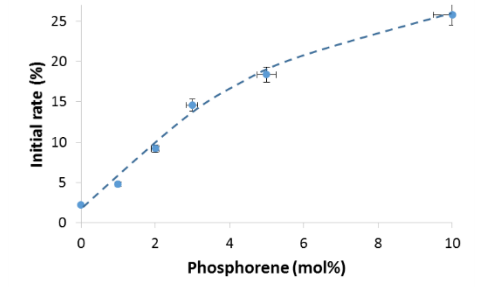


**Supplementary Figure 28.** Reaction order for each reagent in the alkylation reaction (Table 2) with FL–BP catalyst. Similar lines obtained for FL–Sb. Error bars account for 5% uncertainty. Source data are provided as a Source Data file.

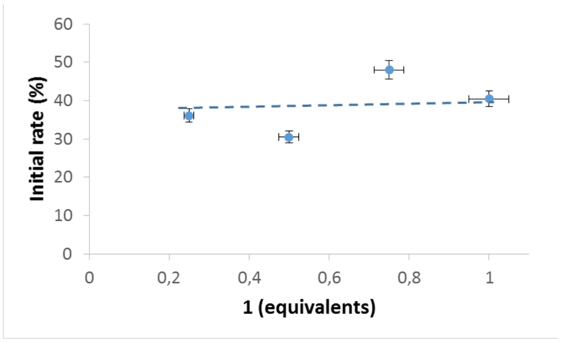

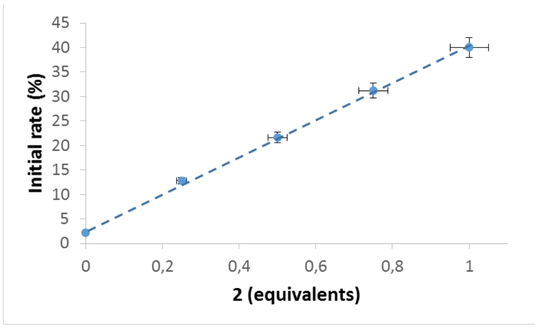

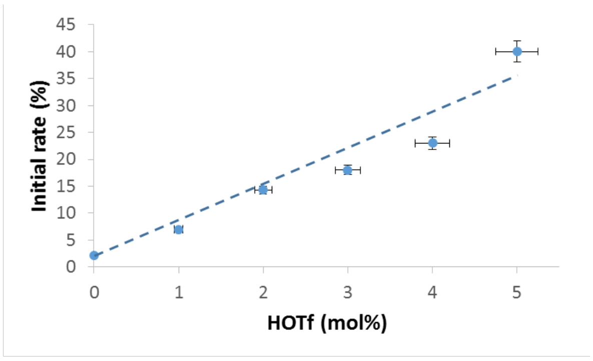


**Supplementary Figure 29.** Reaction order for each reagent in the alkylation reaction of **1** with **2** in the presence of HOTf catalyst at 25 ºC. Error bars account for 5% uncertainty. Source data are provided as a Source Data file.

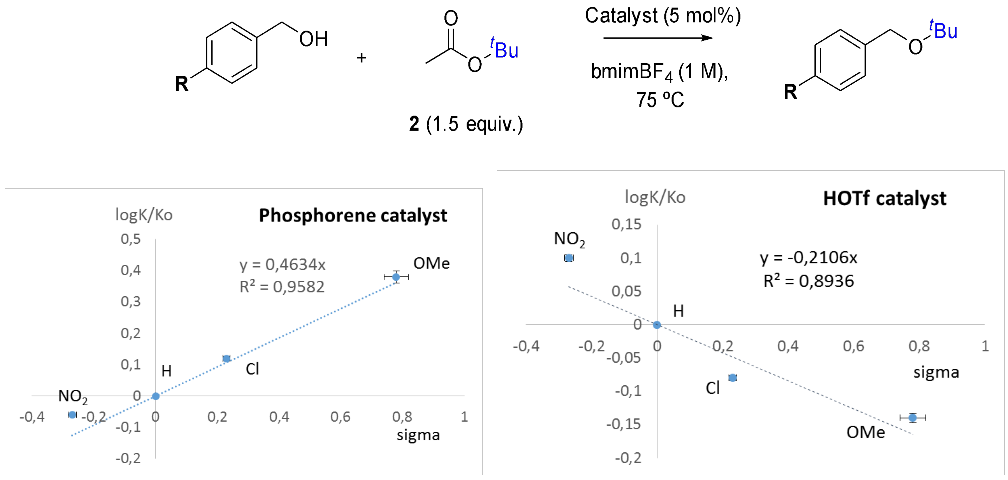


**Supplementary Figure 30.** Hammett–plot for the reaction above with FL–BP (left) and triflic acid (HOTf, right) catalyst. Error bars account for 5% uncertainty. Source data are provided as a Source Data file.

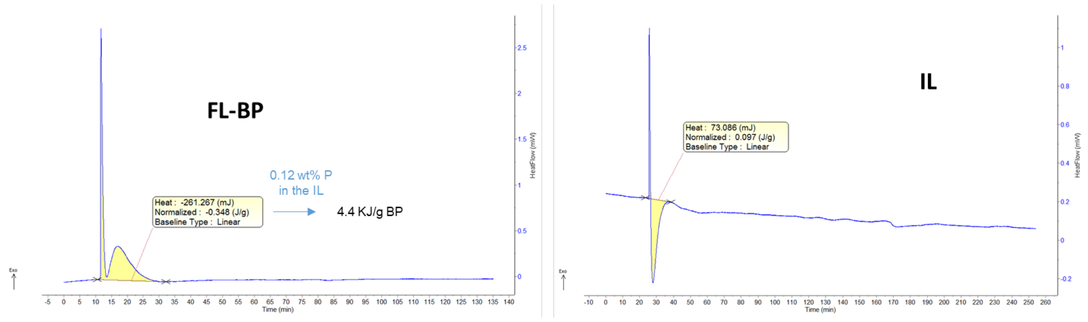


**Supplementary Figure 31** Calorimetry results for the adsorption of **1** on FL-BP (left) and neat IL (right).

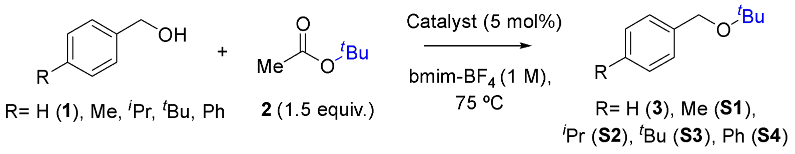


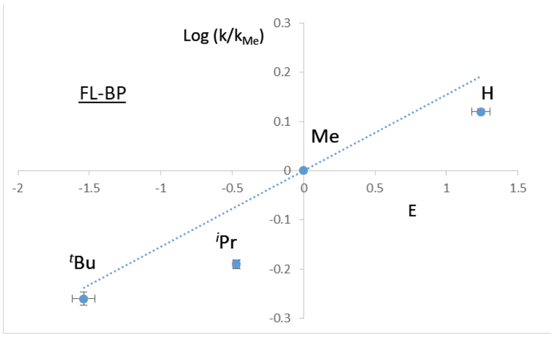


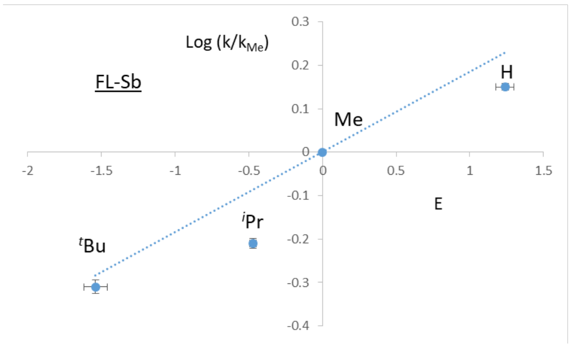


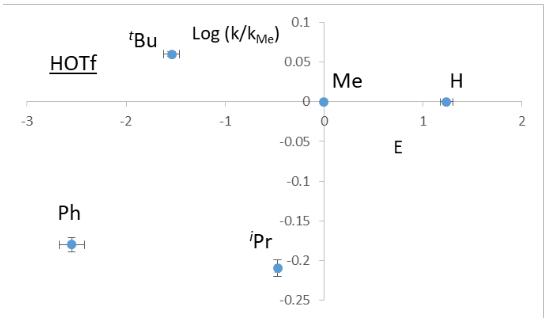


**Supplementary Figure 32.** Taft plots for FL-BP, FL-Sb and HOTf (0.5 mol%), under reaction conditions in Table 2, otherwise indicated. Error bars account for 5% uncertainty. Source data are provided as a Source Data file.

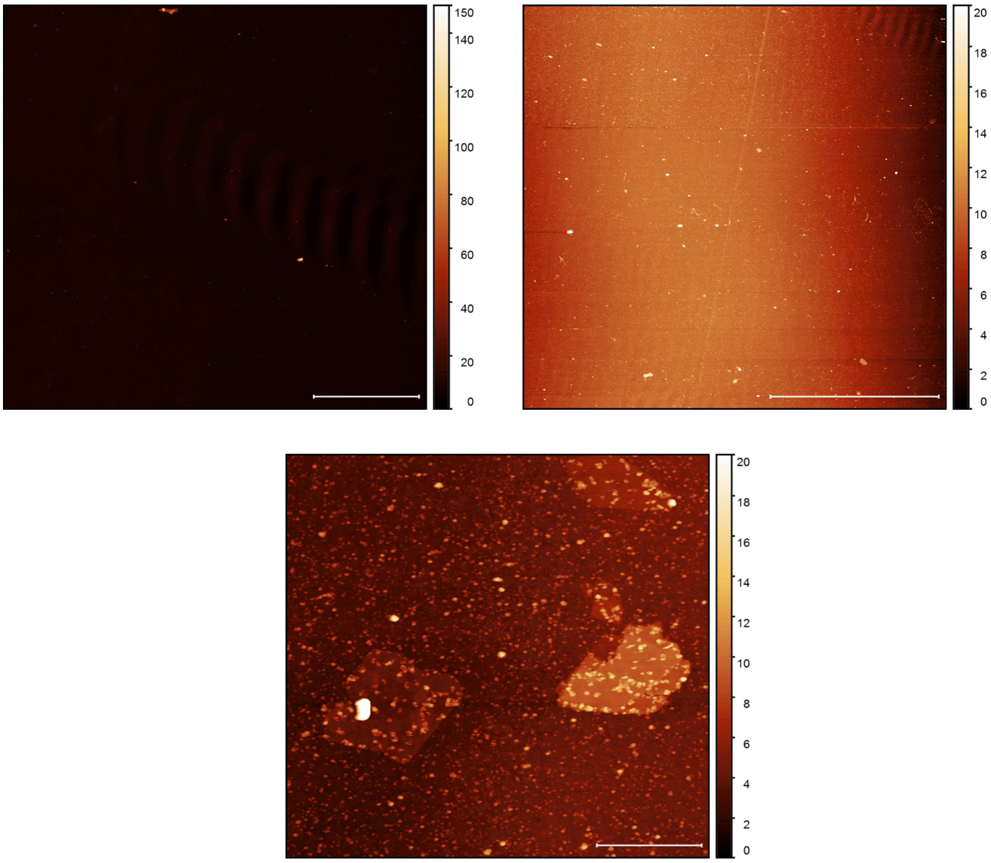


**Supplementary Figure 33.** AFM topography image of the exfoliated FL–BP sample prepared without ultracentrifugation (left, scale bar represents 20 μm ). AFM topography image of the same sample submitted to ultracentrifugation (2 h at 13.000 rpm) (right, scale bar represents 20 μm ), and zoom–in of some thin flakes with minimum thicknesses of *ca*. 1.5 nm (bottom, scale bar represents 500 nm).

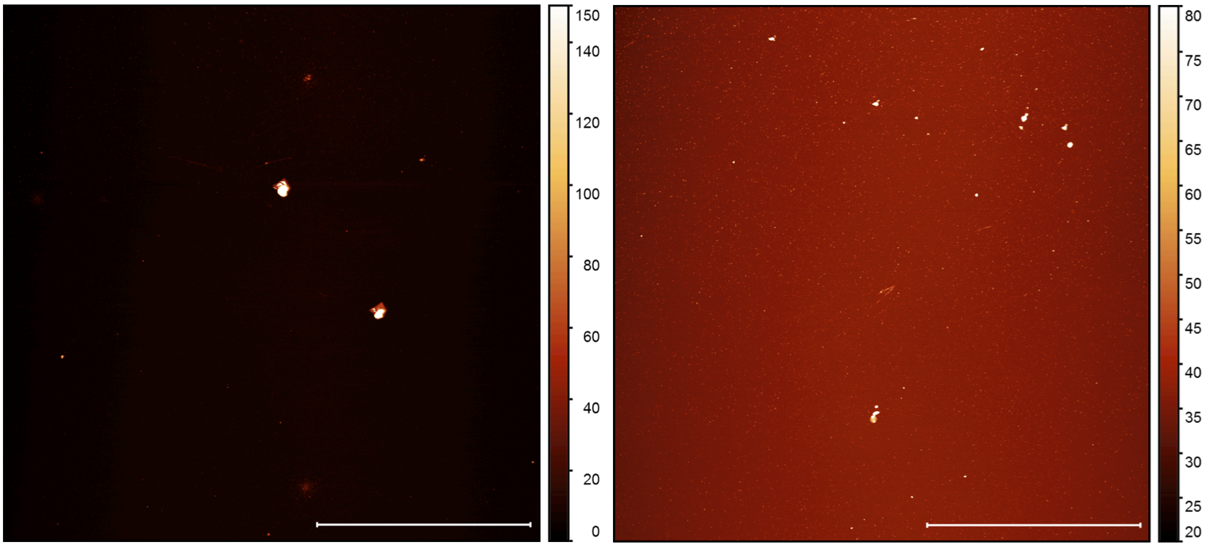


**Supplementary Figure 34.** AFM topography image of the exfoliated FL–Sb sample prepared without ultracentrifugation (left, scale bar represents 20 μm). AFM topography image of the same sample submitted to ultracentrifugation (2h at 13.000 rpm) (right, scale bar represents 20 μm). Source data are provided as a Source Data file.

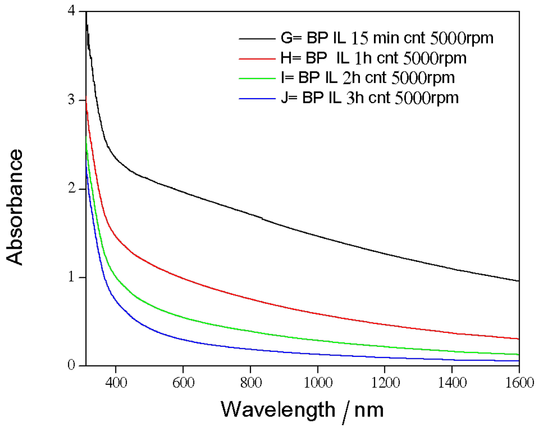


**Supplementary Figure 35.** Absorption visible spectra for FL-BP centrifuged for different times. Black and blue lines correspond to 0.12 and 0.01 mmol P $g^{-1}$, respectively. It is worth to remark the high quality of the solvent used for the dispersions, which is reflected in the absence of water absorption peaks in the NIR spectra at around 1430 nm. Source data are provided as a Source Data file.

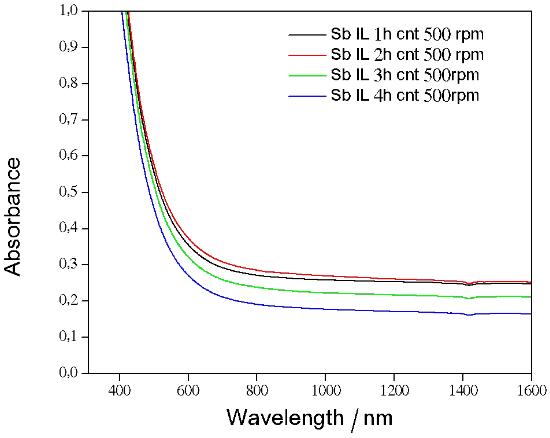


**Supplementary Figure 36.** Absorption visible spectra for FL-Sb centrifuged for different times. Black and blue lines correspond to 0.15 and 0.02 mmol Sb $g^{-1}$, respectively. Source data are provided as a Source Data file.

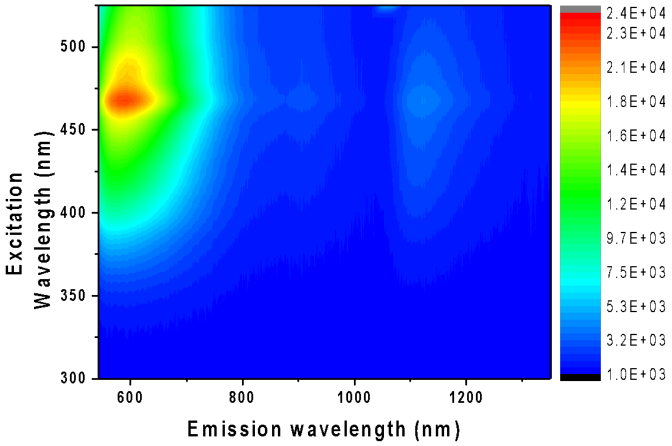


**Supplementary Figure 37.** Photoluminescence emission–excitation contour maps measured on a FL-BP dispersion centrifuged for 3h at 5000 rpm under inert gas conditions (see Methods) measured with a 550 nm cut-off filter in emission. According to Hanlon et al. (*Nature Communications*, 2015, *6*, 8563) the observed emission lines at ~600, 900, and 1125 nm can be associated with PL from 1, 2, and 3 layers of BP, respectively. Source data are provided as a Source Data file.

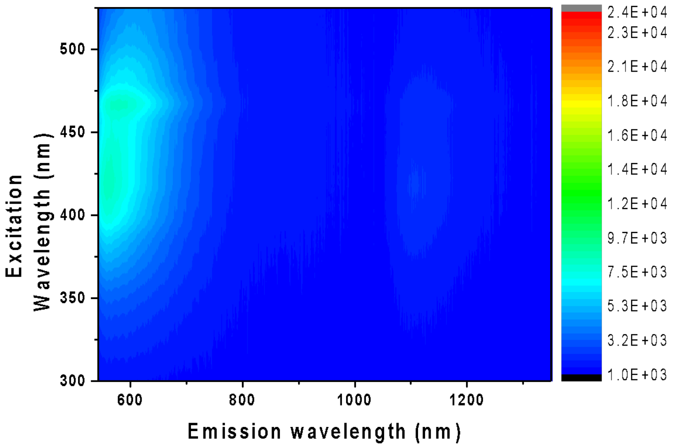


**Supplementary Figure 38.** Photoluminescence emission–excitation contour maps measured on pristine bmim-$BF_4$ under inert gas conditions (see Methods) measured with a 550 nm cut-off filter in emission. The observed weak emissions have been attributed to supramolecular aggregates (Bath et al. *Journal of Molecular Liquids*, 2013, *181*, 142–151 and references therein). Source data are provided as a Source Data file.

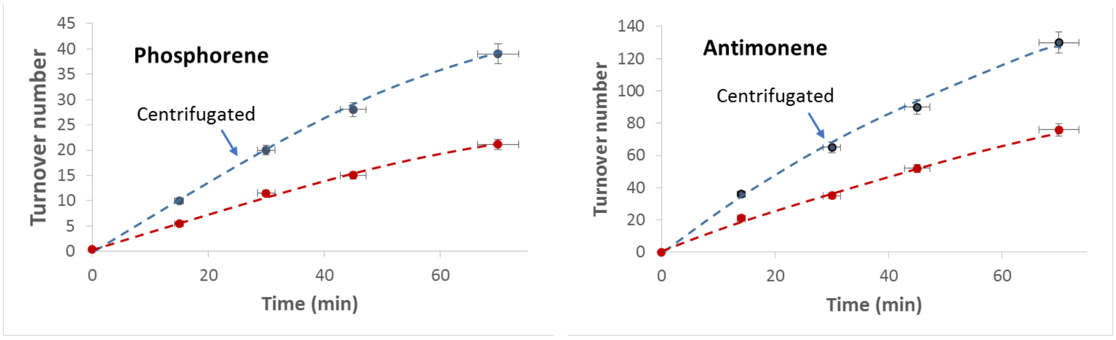


**Supplementary Figure 39.** Turnover number–time plot for FL–BP (left) and FL–Sb (right) before (red circles) and after centrifugation (blue circles). Error bars account for 5% uncertainty. Source data are provided as a Source Data file.

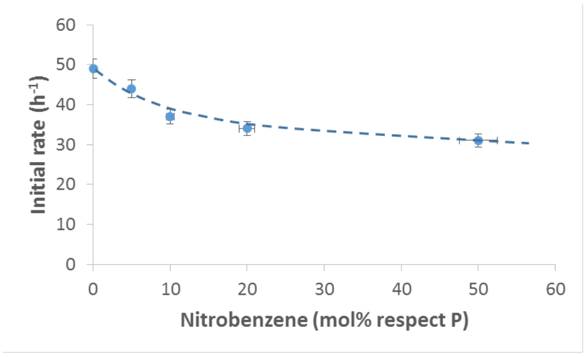


**Supplementary Figure 40.** Inhibition kinetics with nitrobenzene in the presence of FL–BP (10 mol%). Error bars account for 5% uncertainty. Source data are provided as a Source Data file.

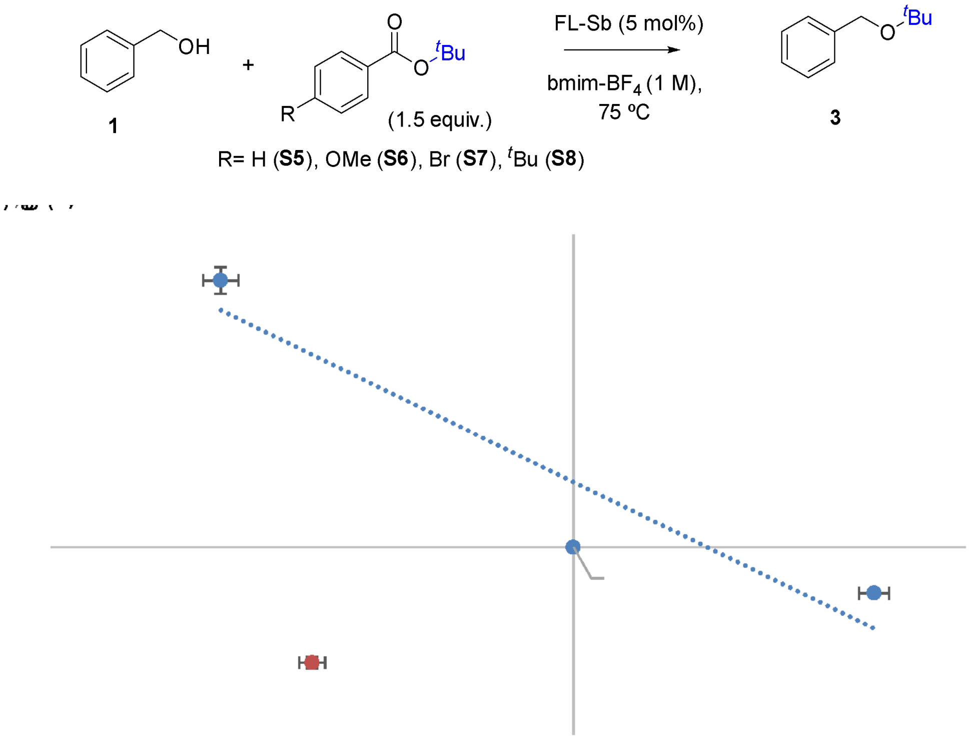


**Supplementary Figure 41.** Hammett plot for the tert-butylation of **1** with different benzoates under FL-Sb catalysis. Error bars account for 5% uncertainty. Source data are provided as a Source Data file.

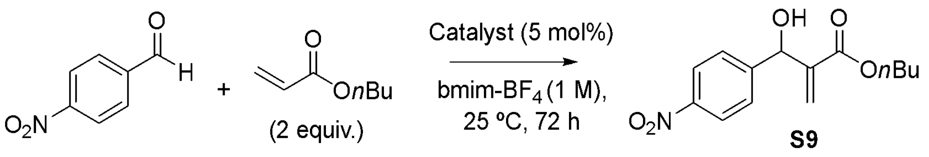


| FL-P / Fl-Sb | **S9** (TON) |
|---|---|
| Non-centrifugated | 0.6 / 1.0 |
| Centrifugated | 1.8 / 2.3 |
| DABCO | 1.0 |
| Blank | 0 |

**Supplementary Figure 42.** Baylis-Hillman reaction catalysed by FL-P, FL-Sb or DABCO in IL.

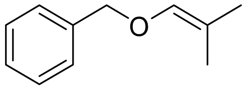

**Supplementary Table 1** Reported procedures to synthesize the allylic benzyl alcohol above (see references below).

| Entry | Reaction | Yield (%) |
|---|---|---|
| 1[1] | 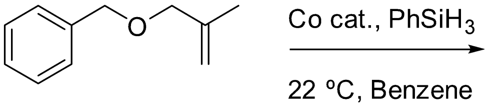 | 81 |
| 2[2] | 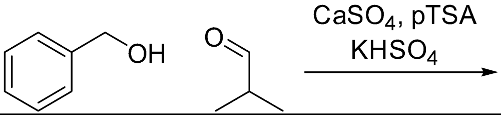 | - |
| 3[2] | 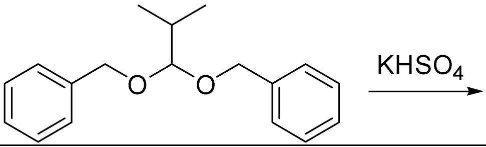 | 70 |
| 4[3] | 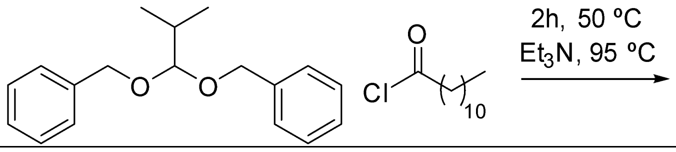 | 69 |
| 5[4] | 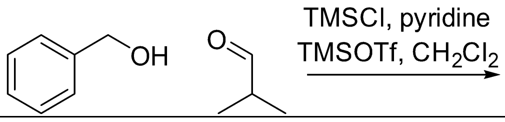 | - |
| 6[4] | 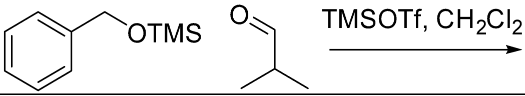 | 42 |
| 7[5] | 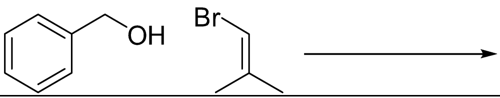 | - |
| 8[6] | 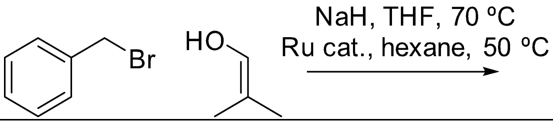 | - |
| 9[6] | 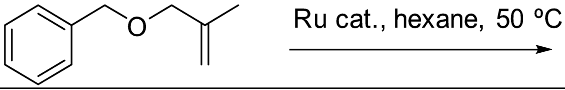 | 95 |
| 10[7] | 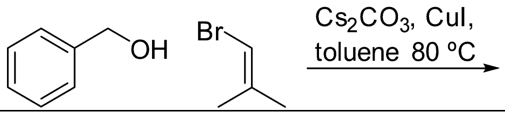 | 64 |
| 11[8] | 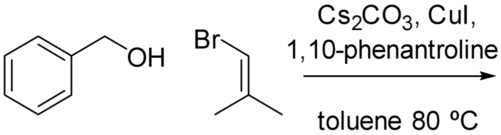 | 50 |

| | | |
|---|---|---|
| 12[9] | 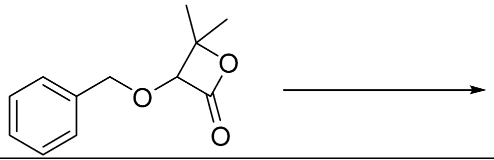 | 85 |

**Supplementary Table 2** Data showing the Taft parameters (Es and σ) for the substituents and log($k/k_{Me}$) for all the catalysts in competitive experiments. For reaction conditions see Table 1.

| Group | Es | σ | HOTf | FL-BP | FL-Sb |
|---|---|---|---|---|---|
| *H* | 1.24 | 0.49 | 0.182 | -0.118 | 0.343 |
| *Me* | 0 | 0 | 0 | 0 | 0 |
| *iPr* | -0.47 | -0.19 | 0.223 | -0.028 | 0.375 |
| *tBu* | -1.54 | -0.3 | -0.980 | -0.639 | -0.383 |
| *Ph* | -2.55 | 0.6 | -1.674 | n.m. | n.m. |

**Supplementary Table 3** Multiple regression analysis of the different catalysts in competitive experiments. S.D. = standard deviation. δ = steric effects. $\rho^*$ = polar effects.

| | Intercept | S.D. | δ | S.D. | ρ* | S.D. |
|---|---|---|---|---|---|---|
| *HOTf* | -0.213 | 0.163 | -0.680 | 0.479 | 2.280 | 1.578 |
| *FL-BP* | -0.038 | 0.029 | 0.825 | 0.084 | -2.229 | 0.278 |
| *FL-Sb* | -0.160 | 0.122 | 0.276 | 0.359 | 0.424 | 1.182 |

**Supplementary Table 4** Multiple regression analysis of the different catalysts in competitive experiments forcing the analysis to intercept the origin of coordinates (0, 0, 0). S.D. = standard deviation. δ = steric effects. $\rho^*$ = polar effects.

| | Intercept | S.D. | δ | S.D. | ρ* | S.D. |
|---|---|---|---|---|---|---|
| *HOTf* | 0 | -- | -0.325 | 0.460 | 1.156 | 1.545 |
| *FL-BP* | 0 | -- | 0.887 | 0.081 | -2.427 | 0.272 |
| *FL-Sb* | 0 | -- | 0.542 | 0.345 | -0.418 | 1.157 |

**Supplementary Table 5** Comparison between graphene and graphene-based catalysts with traditional catalyst in common reactions in organic chemistry. G = graphene; GO = graphene oxide; rGO = reduced graphene oxide; (X)G = graphene doped with X).

| Entry | Reaction | Catalyst | Temperature | Yield (%) |
|---|---|---|---|---|
| 1[10] | 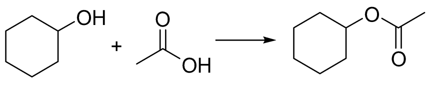 | G-SO3H | 100 ºC | 79.5 |
| 2[10] | | GO | 100 ºC | 11.5 |
| 3[10] | | rGO | 100 ºC | 10.2 |
| 4[11] | | Fe2O3/C | 116 ºC | 97 |
| 5[10] | 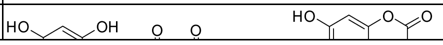 | G-SO3H | 110 ºC | 82.1 |

| | | | | |
|---|---|---|---|---|
| 6[10] | | GO | 110 ºC | 12.3 |
| 7[10] | | rGO | 110 ºC | 11.1 |
| 8[12] | | H2SO4 | 25 ºC | 97 |
| 9[10] | 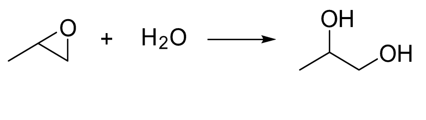 | G-SO3H | 27 ºC | 66.8 |
| 10[10] | | GO | 27 ºC | 0 |
| 11[10] | | rGO | 27 ºC | 0 |
| 12[13] | | Co-salen | 40 ºC | 97 |
| 13[14] | 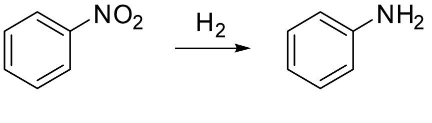 | rGO | 100 ºC | 94.2 |
| 14[14] | | GO | 100 ºC | 91.1 |
| 15[15] | | Pd | 25 ºC | 100 |
| 16[16] | Selective acetylene hydrogenation | G | 110 ºC | 81 |
| 17[16] | | rGO | 110 ºC | 87.5 |
| 18[17] | | PdIn/Al2O3 | 60 ºC | 95 |
| 19[18] | 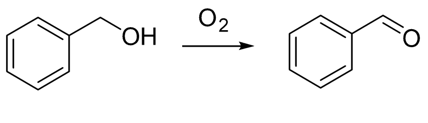 | GO 200 wt% | 100 ºC | 92 |
| 20[19] | | Pt/TiO2 | 25 ºC | 76.7 |
| 21[18] | 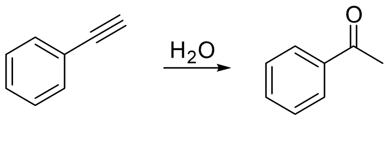 | GO 200 wt% | 100 ºC | 98 |
| 22[20] | | TfOH | 25 ºC | 100 |
| 23[21] | 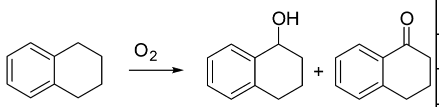 24 h, 1 bar O2 | - | 120 ºC | 8.2 |
| 24[21] | | GO | 120 ºC | 18.4 |
| 25[21] | | G | 120 ºC | 14.7 |
| 26[21] | | (N)G | 120 ºC | 34.0 |
| 27[21] | | (B)G | 120 ºC | 31.1 |
| 28[21] | | (B,N)G | 120 ºC | 40 |

**Supplementary Note 1**

**Compound characterization.**

**(*Tert*–butoxymethyl)benzene 3**. IR (ν, $cm^{-1}$): 2975, 2867, 1455, 1363, 1197, 1068, 913, 745. $^1H$ NMR (™, ppm; J, Hz): 7.27 (5H, m), 4.38 (2H, s), 1.22 (9H, s). $^{13}C$ NMR (™, ppm; J, Hz): 139.9 (C), 128.3 (2 x CH), 127.4 (2 x CH), 127.1 (CH), 73.4 (C), 64.1 ($CH_2$), 27.7 (3 x $CH_3$). HRMS (ESI) [$M+H^+$, major peak; calculated for $C_{11}H_{16}O$: 165.1280] found 165.1282 *m/z*.

**1–(*Tert*–butoxymethyl)–2–iodobenzene 4**. IR (ν, $cm^{-1}$): 3062, 2973, 2869, 1468, 1363, 1195, 1085, 1012, 746. $^1H$ NMR (™, ppm; J, Hz): 7.71 (1H, d, J = 7.9), 7.43 (1H, d, J = 7.7), 7.27 (1H, t, J = 7.5), 6.88 (1H, t, 7.6). $^{13}C$ NMR (™, ppm; J, Hz): 142.0 (C), 138.8 (CH), 128.7 (CH), 128.6 (CH), 128.2 (CH), 97.3 (C), 73.8 (C), 68.4 ($CH_2$), 27.7 (3 x $CH_3$). HRMS (ESI) [$M^+$, major peak; calculated for $C_{11}H_{15}IO$: 290.0168] found 290.0163 *m/z*.

**((*E*)–3–(((*E*)–3–Iodobut–2–en–1–yl)oxy)prop–1–en–1–yl)benzene 5**. IR (ν, $cm^{-1}$): 3457, 2921, 1677, 1626, 1125, 974, 750, 689. $^1H$ NMR (™, ppm; J, Hz): 7.36–7.16 (5H, m), 6.54 (1H, d, J = 15.9), 6.31 (1H, t, J = 6.9), 6.20 (1H, dt, J = 15.9, 6.1), 4.07 (2H, dd, J = 6.1, 1.3), 3.91 (2H, d, J = 6.9), 2.95 (3H, s). $^{13}C$ NMR (™, ppm; J, Hz): 193.7 (C), 152.7 (C), 129.1 (CH), 128.6 (CH), 128.5 (2 x CH), 127.8 (CH), 126.5 (2 x CH), 125.6 (CH), 70.7 ($CH_2$), 66.6 ($CH_2$), 28.2 ($CH_3$). HRMS (ESI) [M+, major peak; calculated for $C_{13}H_{15}IO$: 314.0168] found 314.0171 *m/z*.

**9–((1phenylethyl)thio)nonan–1–ol, 6**. $^1H$ NMR (™, ppm; J, Hz): 7.21 (5H, m), 3.87 (1H, q, J = 7.1), 3.21 (2H, t, J = 6.7), 2.22 (2H, m), 1.61–1.45 (8H, m), 1.36 (3H, d, 6.5), 1.30–1.10 (6H, m). $^{13}C$ NMR (™, ppm; J, Hz): 144.3 (C), 128.3 (2 x CH), 127.2 (CH), 126.1 (2 x CH), 68.8 ($CH_2$), 44.1 (CH), 39.2 ($CH_2$), 31.3 ($CH_2$), 29.9 ($CH_2$), 29.3 (2 x $CH_2$), 29.1 ($CH_2$), 28.9 ($CH_2$), 26.1 ($CH_2$), 24.2 ($CH_3$).

**4–(*Tert*–butylthio)phenol 7**. IR (ν, $cm^{-1}$): 3378, 2965, 1598, 1494, 1363, 1166, 832, 529. $^1H$ NMR (™, ppm; J, Hz): 7.32 (2H, d, J = 8.7), 6.72 (2H, d, J = 8.7), 1.18 (9H, s). $^{13}C$ NMR (™, ppm; J, Hz): 156.4 (C), 139.1 (2 x CH), 123.7 (C), 115.5 (2 x CH), 45.6

(C), 30.7 (3 x $CH_3$). HRMS (ESI) [$M–H^+$, major peak; calculated for $C_{10}H_{13}OS$: 181.0687] found 181.0680 *m/z.*

**3–(*Tert*–butyl)–1*H*–indole 8a**. IR (ν, $cm^{-1}$): 3417, 3063, 2962, 1702, 1461, 1361, 743. $^{1}H$ NMR (™, ppm; J, Hz): 7.79 (1H, s), 7.75 (1H, d, J = 7.8), 7.29 (1H, d, J = 8.1), 7.10 (1H, t, J = 7.5), 7.02 (1H, t, J = 7.5), 6.87 (1H, s), 1.78 (2H, s), 1.39 (9H, s). $^{13}C$ NMR (™, ppm; J, Hz): 137.2 (C), 126.8 (C), 126.0 (C), 121.4 (CH), 121.3 (CH), 119.2 (CH), 118.8 (CH), 111.3 (CH), 31.6 (C), 30.7 (3 x $CH_3$). HRMS (ESI) [$M^+$, major peak; calculated for $C_{12}H_{16}N$: 174.1283] found 174.1281 *m/z.*

**1,3–Di–*tert*–butyl–1*H*–indole 8b**. IR (ν, $cm^{-1}$): 2963, 1711, 1481, 1222, 914, 743. $^{1}H$ NMR (™, ppm; J, Hz): 7.75 (1H, d, J = 7.8), 7.54 (1H, d, J = 8.4), 7.06 (1H, t, J = 7.6), 6.98 (1H, t, J = 8.0), 6.91 (1H, s), 1.64 (9H, s), 1.35 (9H, s). HRMS (ESI) [$M^+$, major peak; calculated for $C_{16}H_{24}N$: 230.1909] found 230.1901 *m/z.*

**3–(*Tert*–butyl)–5–methoxy–1*H*–indole 9a**. IR (ν, $cm^{-1}$): 3417, 2962, 1702, 1482, 1224, 1042, 796. $^{1}H$ NMR (™, ppm; J, Hz): 7.68 (1H, s), 7.18 (2H, m), 6.86 (1H, s), 6.78 (1H, dd, J = 8.8, 2.4), 3.81 (3H, s), 1.37 (9H, s). $^{13}C$ NMR (™, ppm; J, Hz): 153.2 (C), 132.5 (C), 126.5 (C), 126.3 (C), 120.2 (CH), 111.8 (CH), 111.2 (CH), 104.1 (CH), 56.1 ($CH_3$), 31.5 (C), 30.6 (3 x $CH_3$). HRMS (ESI) [$M+H^+$, major peak; calculated for $C_{13}H_{17}NO$: 204.1389] found 204.1385 *m/z.*

**1,3–Di–*tert*–butyl–5–methoxy–1*H*–indole 9b**. IR (ν, $cm^{-1}$): 2966, 1709, 1479, 1227, 1038, 668. $^{1}H$ NMR (™, ppm; J, Hz): 7.43 (1H, d, J = 9.1), 7.19 (1H, s), 6.88 (1H, s), 6.74 (1H, dd, J = 9.1, 2.6), 3.80 (3H, s), 1.61 (9H, s), 1.36 (9H, s). HRMS (ESI) [$M+H^+$, major peak; calculated for $C_{17}H_{25}NO$: 260.2014] found 260.2003 *m/z.*

***N*–(2–(5–(*tert*–butoxy)pentanoyl)–4,5–dimethoxyphenethyl)–2,2,2–trifluoroacetamide 11**. IR (ν, $cm^{-1}$): 3325, 2968, 1720, 1518, 1360, 1198. $^{1}H$ NMR (™, ppm; J, Hz): 7.13 (1H, s), 6.66 (1H, s). HRMS (ESI) [$M+H^+$, major peak; calculated for $C_{21}H_{31}FNO_5$: 434.2155] found 434.2159 *m/z.*

**(R)–1–phenylethyl acetate 13**. $^{1}H$ NMR (™, ppm; J, Hz): 7.20 (5H, m), 5.81 (1H, q, J = 6.6), 1.98 (3H, s), 1.45 (3H, d, J = 6.6). $^{13}C$ NMR (™, ppm; J, Hz): 170.3 (C), 141.7 (C), 128.5 (2 x CH), 127.9 (CH), 126.1 (2 x CH), 72.3 (CH), 22.2 ($CH_3$), 21.3 ($CH_3$).

**1-(*tert*-butoxymethyl)-4-methylbenzene S1**. IR (ν, $cm^{-1}$): 2974, 2926, 2867, 1517, 1472, 1389, 1362, 1197, 1082, 1021, 894, 802, 474. GC-MS [$M^{+}$□; calculated for $C_{12}H_{18}O$: 178.1358] (m/z, $M^{+}$□ 178.2), major peaks found (m/z, relative intensity): 57.2 (10%), 77.2 (15%), 93.2 (17%), 105.2 (100%), 163.2 (6%), 178.2 (7%). $^{1}H$ NMR (™, ppm; J, Hz): 7.15 (2H, d, 7.5), 7.05 (2H, d, 7.8), 4.32 (2H, s), 2.25 (2H, s), 1.21 (9H, s). $^{13}C$ NMR (™, ppm; J, Hz): 136.9 (C), 136.7 (C), 129.0 (2 x CH), 127.5 (2 x CH), 73.3 (C), 64.0 ($CH_2$), 27.7 (3 x $CH_3$), 21.1 ($CH_3$).

**1-(*tert*-butoxymethyl)-4-isopropylbenzene S2**. IR (ν, $cm^{-1}$): 2968, 2930, 2871, 1514, 1467, 1388, 1362, 1235, 1196, 1082, 1019, 895, 818, 542. GC-MS [$M^{+}$□; calculated for $C_{14}H_{22}O$: 206.1671] (m/z, $M^{+}$□ 206.2), major peaks found: 43.2 (11%), 57.3 (13%), 79.2 (12%), 87.3 (12%), 107.2 (23%), 117.2 (19%), 133.2 (100%), 163.2 (8%), 206.2 (9%). $^{1}H$ NMR (™, ppm; J, Hz): 7.19 (2H, d, 8.0), 7.10 (2H, d, 8.1), 4.32 (2H, s), 2.80 (1H, m), 1.21 (9H, s), 1.15 (6H, d, 6.9). $^{13}C$ NMR (™, ppm; J, Hz): 147.8 (C), 137.2 (C), 127.6 (2 x CH), 126.4 (2 x CH), 73.3 (C), 64.0 ($CH_2$), 33.9 (CH), 27.7 (3 x $CH_3$), 24.1 (2 x $CH_3$).

**1-(*tert*-butoxymethyl)-4-(*tert*-butyl)benzene S3**. IR (ν, $cm^{-1}$): 2968, 2904, 2868, 1516, 1473, 1389, 1362, 1268. 1197, 1082, 1019, 896, 815, 568. GC-MS [$M^{+}$□; calculated for $C_{15}H_{24}O$: 220.1827] (m/z, $M^{+}$□ 220.3), major peaks found: 57.3 (31%), 91.2 (17%), 107.2 (30%), 117.2 (19%), 132.2 (13%), 147.2 (100%), 149.2 (30%), 163.2 (19%), 205.2 (8%), 220.3 (8%). $^{1}H$ NMR (™, ppm; J, Hz): 7.27 (2H, d, 8.3), 7.20 (2H, d, 8.3), 4.33 (2H, s), 1.23 (9H, s), 1.20 (9H, s). $^{13}C$ NMR (™, ppm; J, Hz): 150.0 (C), 136.8 (C), 127.3 (2 x CH), 125.2 (2 x CH), 73.3 (C), 64.0 ($CH_2$), 34.5 (C), 31.4 (3 x $CH_3$), 27.7 (3 x $CH_3$).

***tert*-butyl 4-methoxybenzoate S6**. $^{1}H$ NMR (™, ppm; J, Hz): 7.86 (2H, d, 9.0), 6.82 (2H, d, 9.0), 3.77 (3H, s), 1.50 (9H, s). $^{13}C$ NMR (™, ppm; J, Hz): 165.6 (C), 163.0 (C), 131.4 (2 x CH), 124.6 (C), 113.4 (2 x CH), 80.5 (C), 35.0 (C), 55.4 ($CH_3$), 28.3 (3 x $CH_3$).

***tert*-butyl 4-bromobenzoate S7**. $^{1}H$ NMR (™, ppm; J, Hz): 7.86 (2H, d, 8.5), 7.56 (2H, d, 8.5), 1.60 (9H, s). $^{13}C$ NMR (™, ppm; J, Hz): 165.0 (C), 131.5 (2 x CH), 131.0 (2 x CH), 130.9 (C), 127.4 (C), 81.4 (C), 28.2 (3 x $CH_3$).

***tert*-butyl 4-(*tert*-butyl)benzoate S8**. $^{1}H$ NMR (™, ppm; J, Hz): 7.85 (2H, d, 8.4), 7.36 (2H, d, 8.4), 1.51 (9H, s), 1.26 (9H, s). $^{13}C$ NMR (™, ppm; J, Hz): 165.8 (C), 156.0 (C), 129.3 (C), 129.2 (2 x CH), 125.1 (2 x CH), 80.6 (C), 35.0 (C), 31.1 (3 x $CH_3$), 28.2 (3 x $CH_3$).

**Butyl 2-(hydroxy(4-nitrophenyl)methyl)acrylate S9**. GC-MS [$M^{+}$▯; calculated for $C_{14}H_{17}NO_5$: 279.2920] (m/z, $M^{+}$▯ ), major peaks found: 279.1 (8%), 223.1 (27%), 206.1 (100%). $^{1}H$ NMR (™, ppm; J, Hz): 8.00 (4H, m), 6.32 (1H, s), 5.77 (1H, s), 5.55 (1H, s), 4.07 (2H, t, 6.6), 2.10 (1H, s), 1.56 (2H, m), 1.18 (2H, m), 0.84 (3H, t, 7.4).

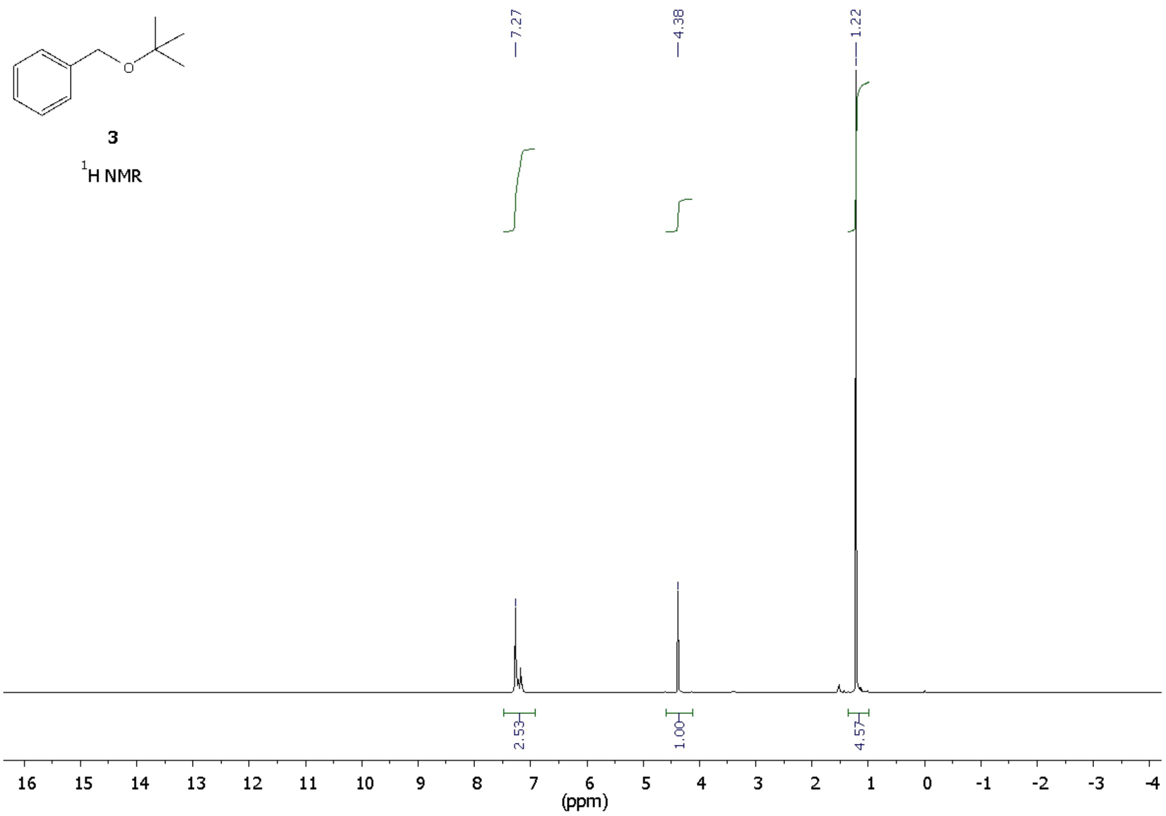

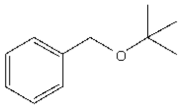

**3**

$^{13}C$ NMR

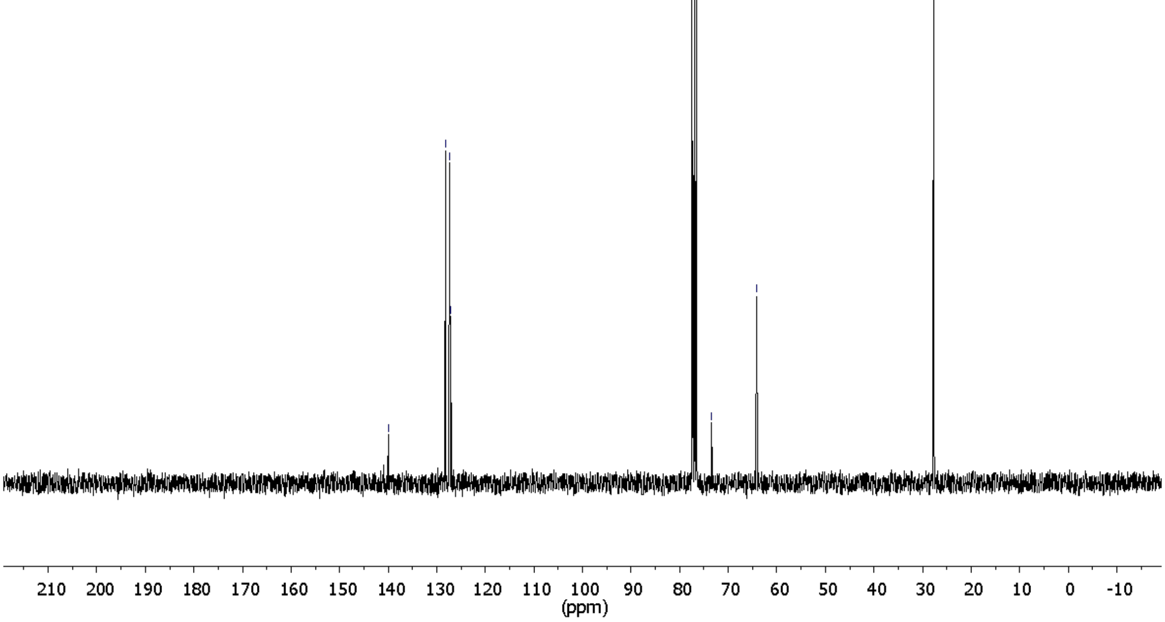


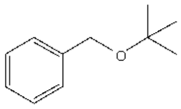

**3**

DEPT

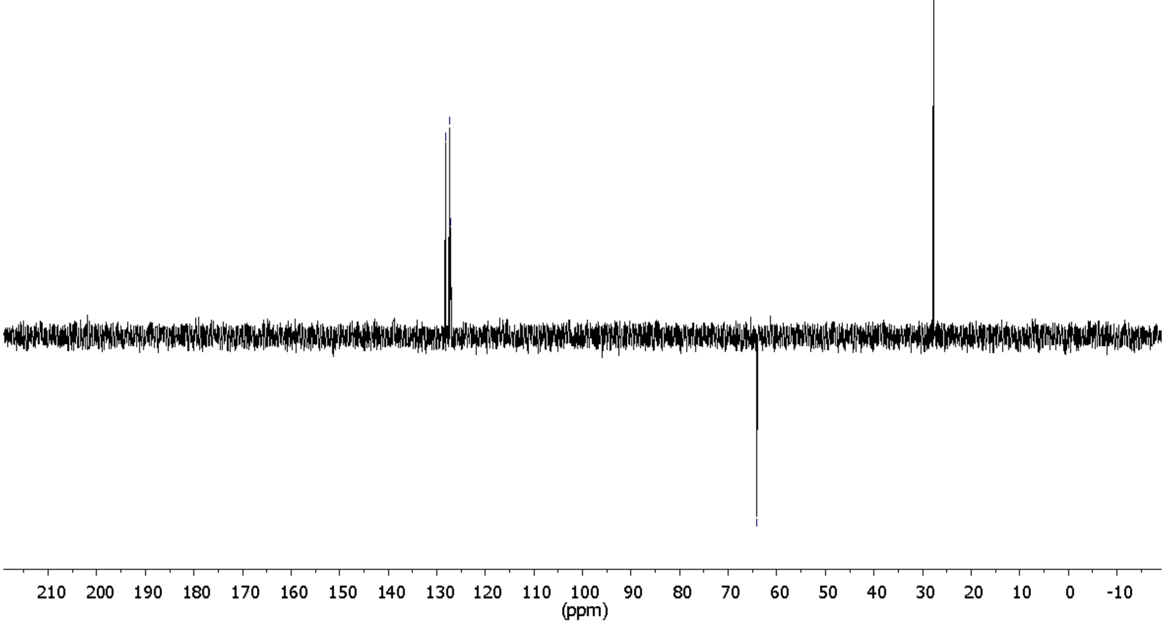

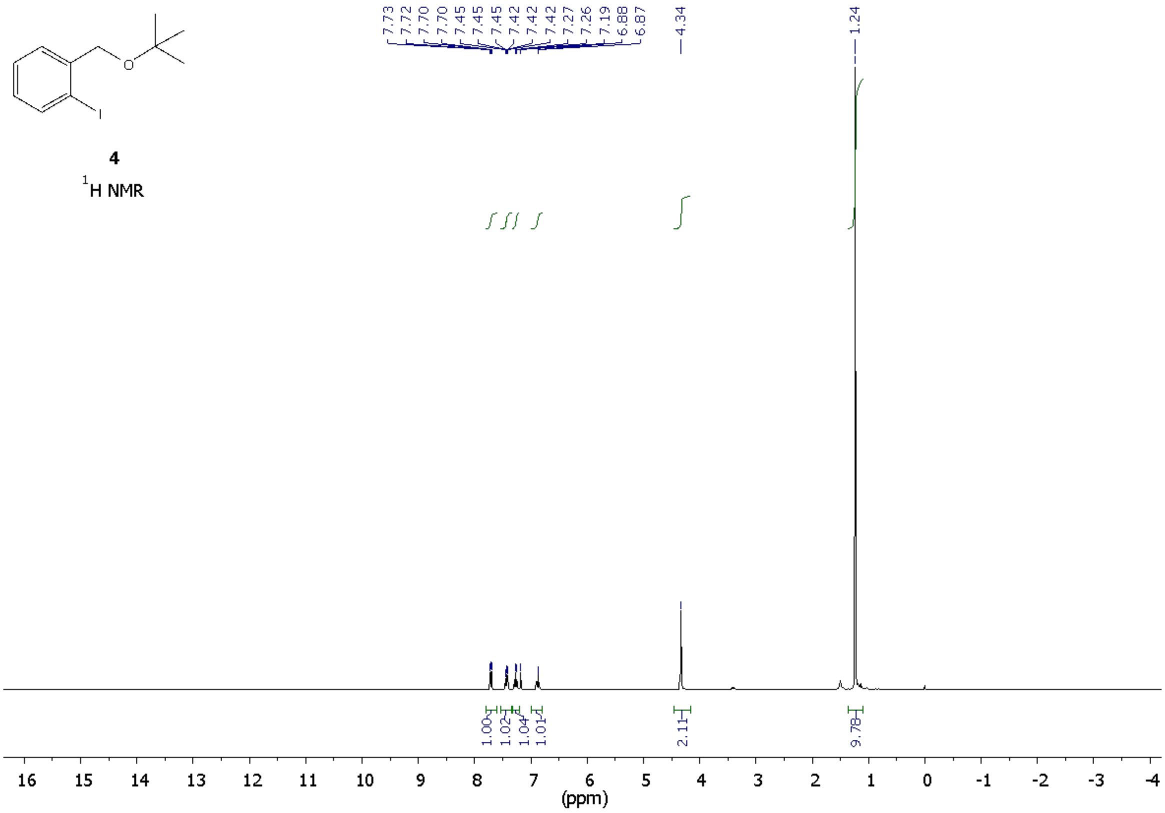


**4**
$^{1}$H NMR

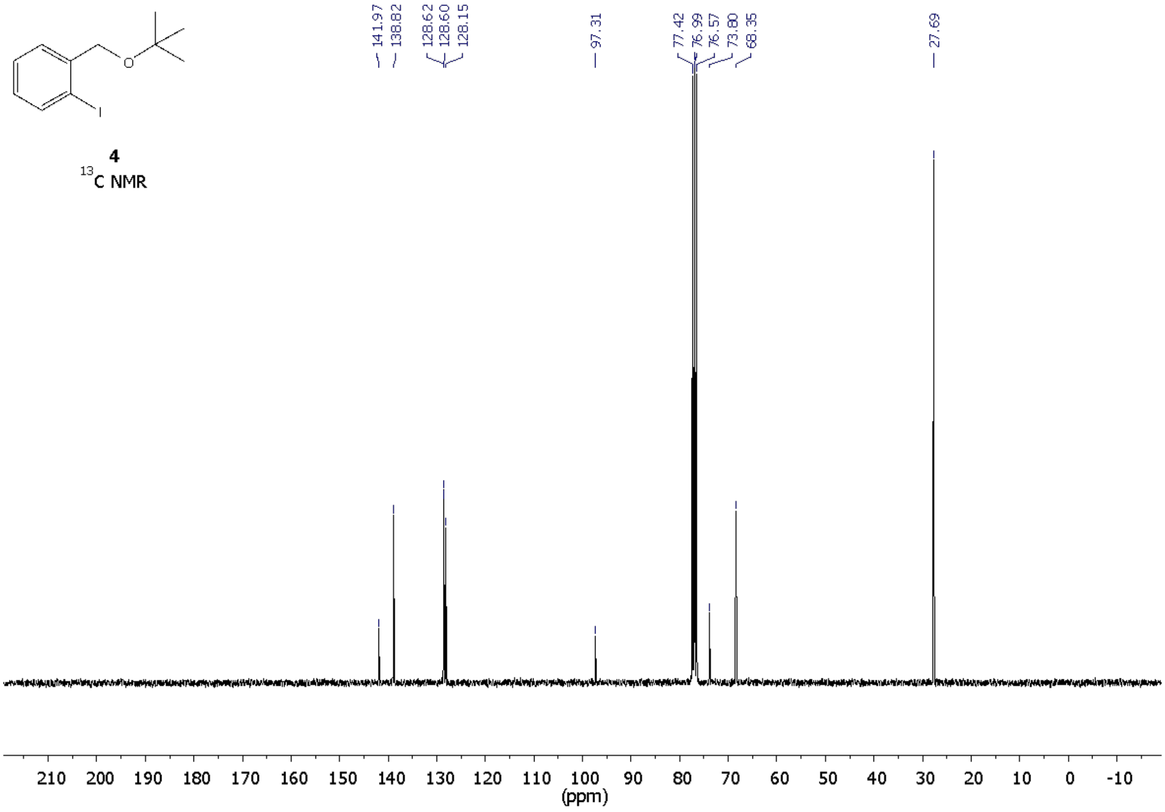


**4**
$^{13}$C NMR

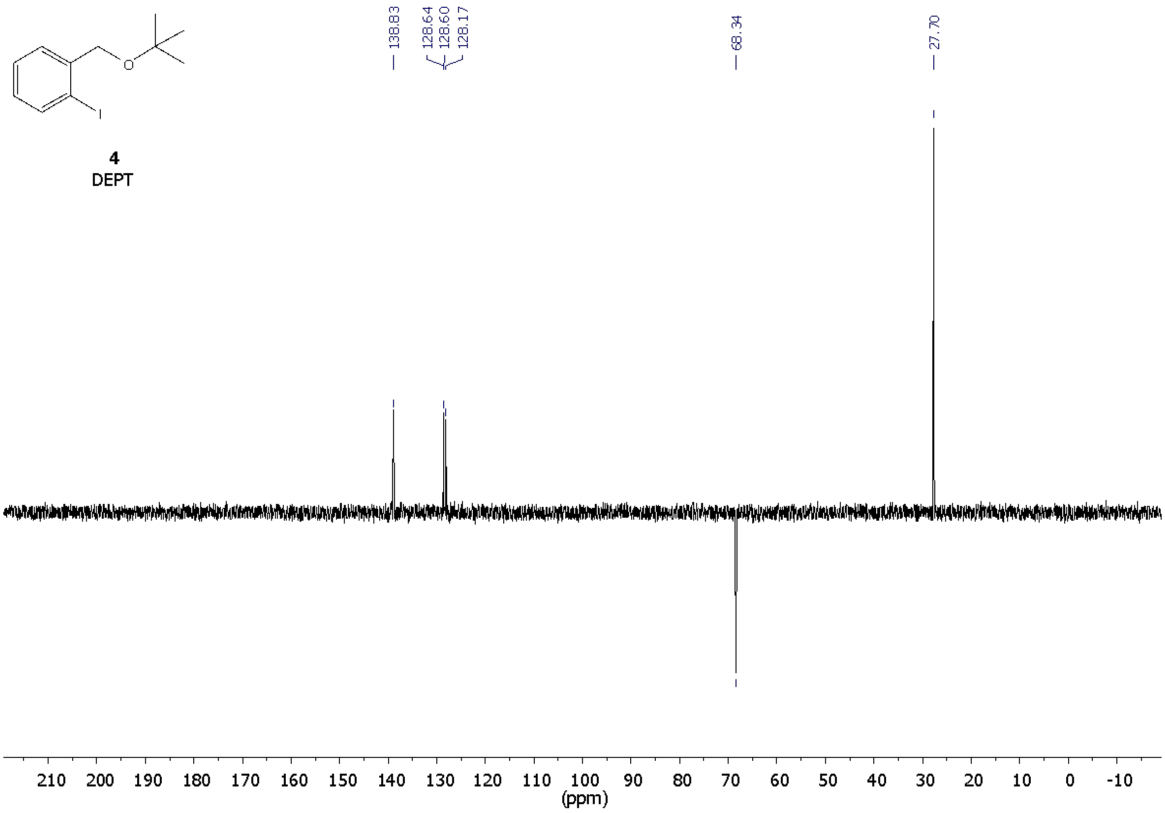
4
DEPT
138.83
128.64
128.60
128.17
68.34
27.70
210 200 190 180 170 160 150 140 130 120 110 100 90 80 70 60 50 40 30 20 10 0 -10
(ppm)

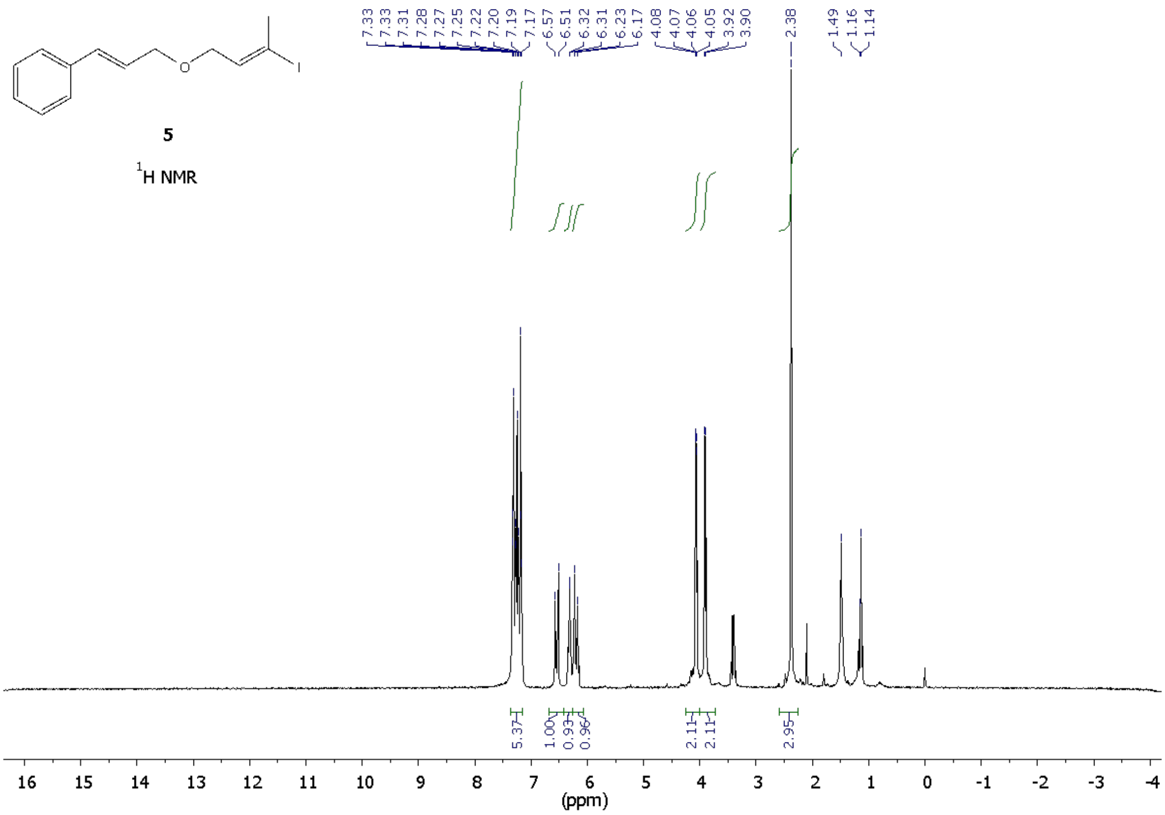
5
1H NMR
7.33 7.33 7.31 7.28 7.27 7.25 7.22 7.20 7.19 7.17 6.57 6.51 6.32 6.31 6.23 6.17 4.08 4.07 4.06 4.05 3.92 3.90 2.38 1.49 1.16 1.14
5.37 1.00 0.93 0.96 2.11 2.11 2.95
16 15 14 13 12 11 10 9 8 7 6 5 4 3 2 1 0 -1 -2 -3 -4
(ppm)

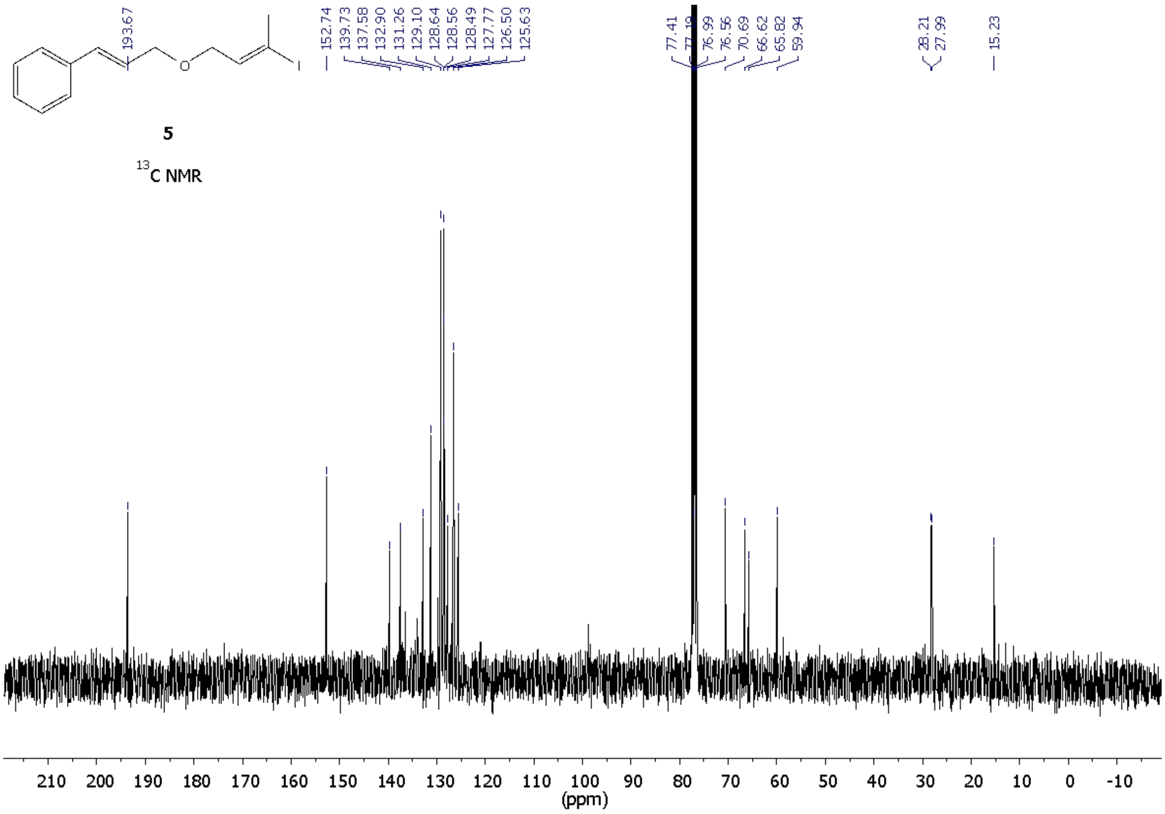
193.67
152.74
139.73
137.58
132.90
131.26
129.10
128.64
128.56
128.49
127.77
126.50
125.63
77.41
77.19
76.99
76.56
70.69
66.62
65.82
59.94
28.21
27.99
15.23
O
I
5
13C NMR
210 200 190 180 170 160 150 140 130 120 110 100 90 80 70 60 50 40 30 20 10 0 -10
(ppm)

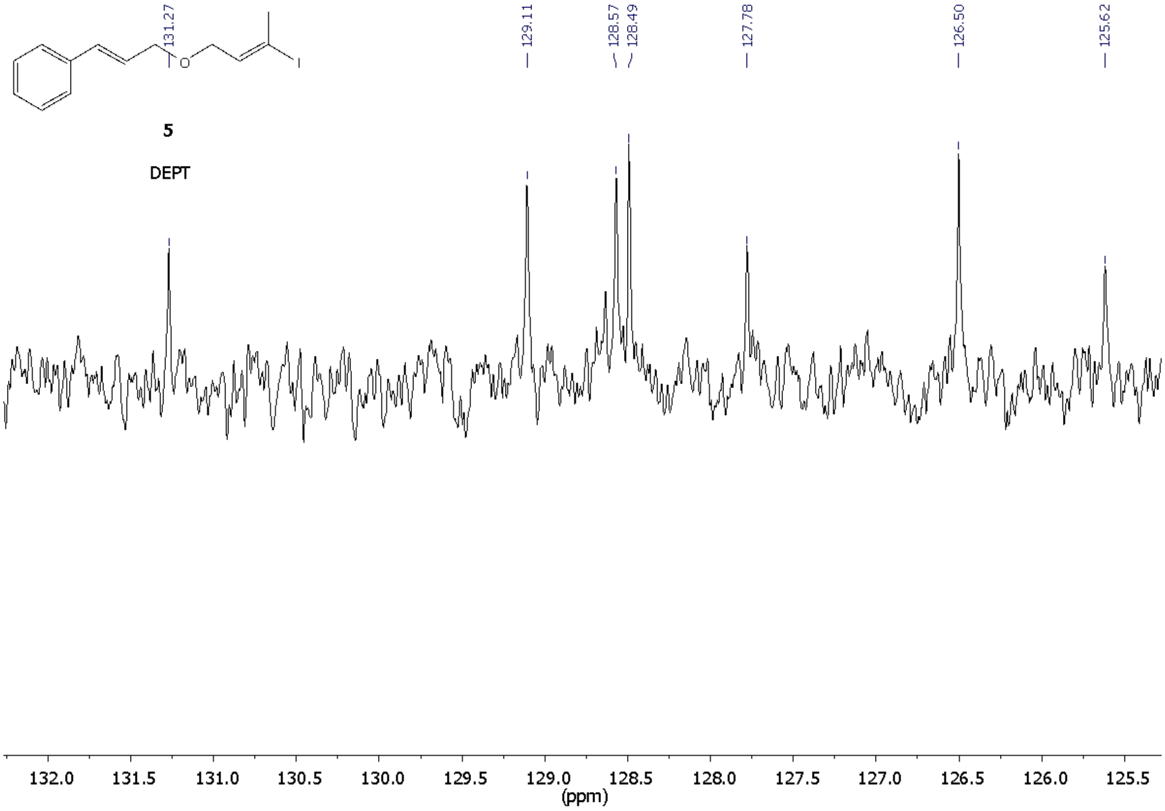
131.27
129.11
128.57
128.49
127.78
126.50
125.62
O
I
5
DEPT
132.0 131.5 131.0 130.5 130.0 129.5 129.0 128.5 128.0 127.5 127.0 126.5 126.0 125.5
(ppm)

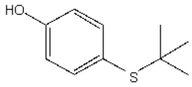

**7**

$^{1}$H NMR

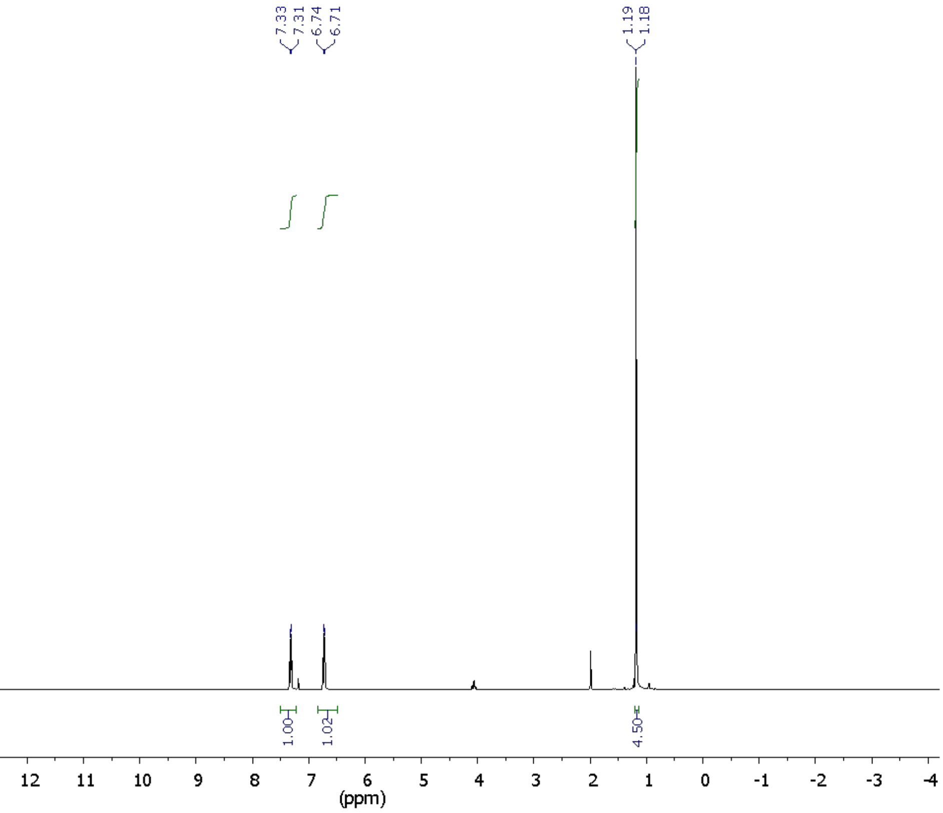


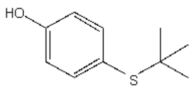

**7**

$^{13}$C NMR

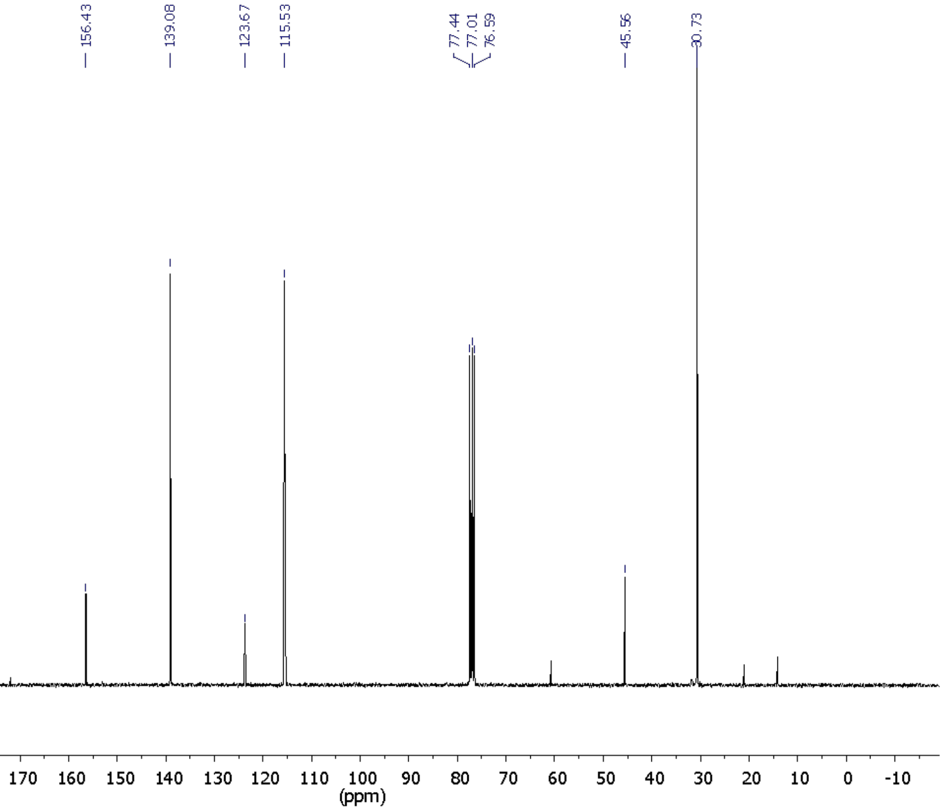

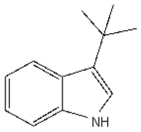


**8a**

$^{1}$H NMR

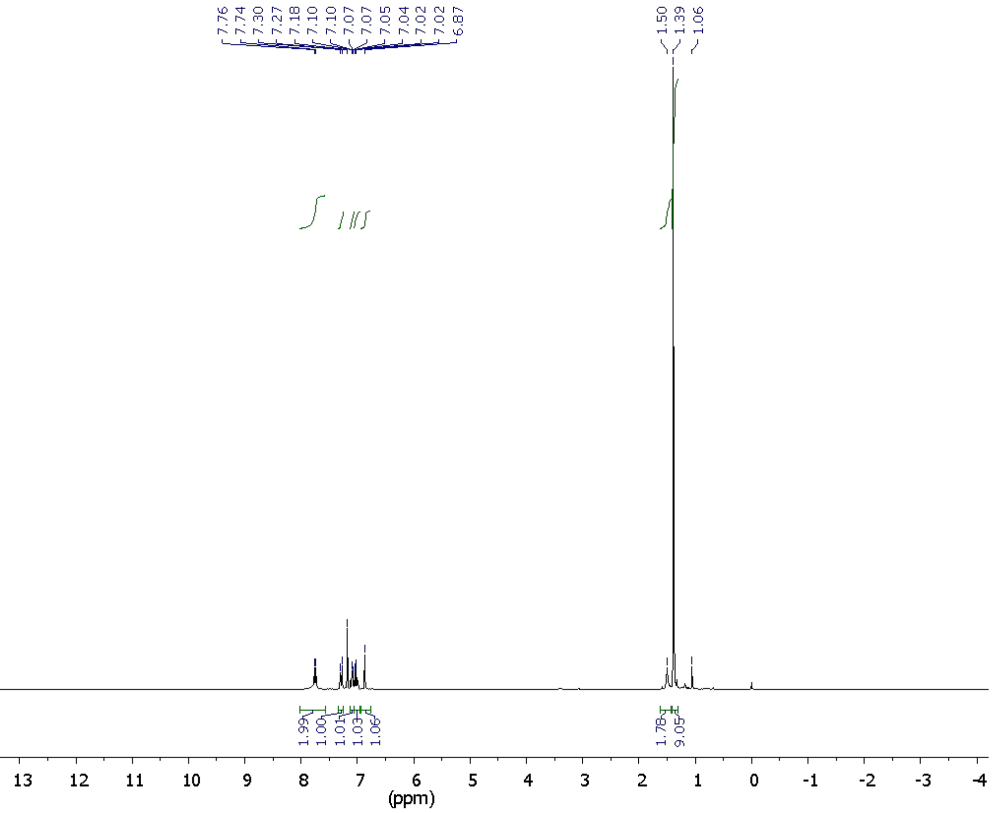


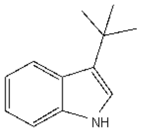


**8a**

$^{13}$C NMR

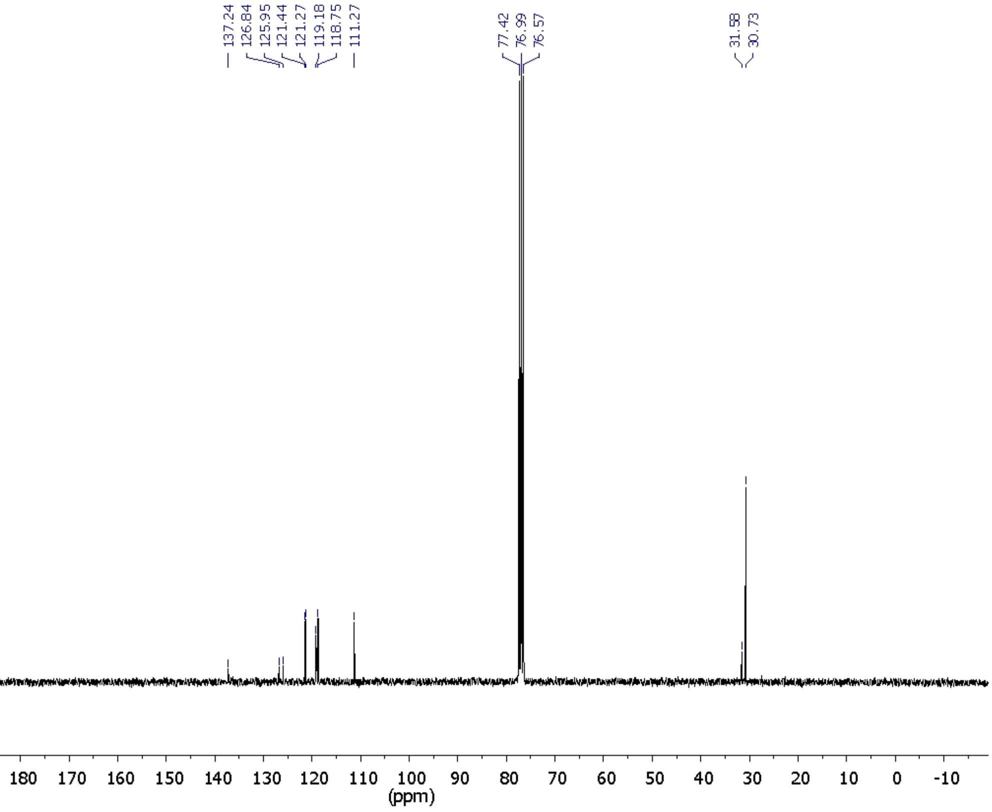

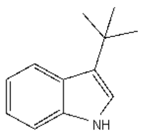

**8a**

DEPT

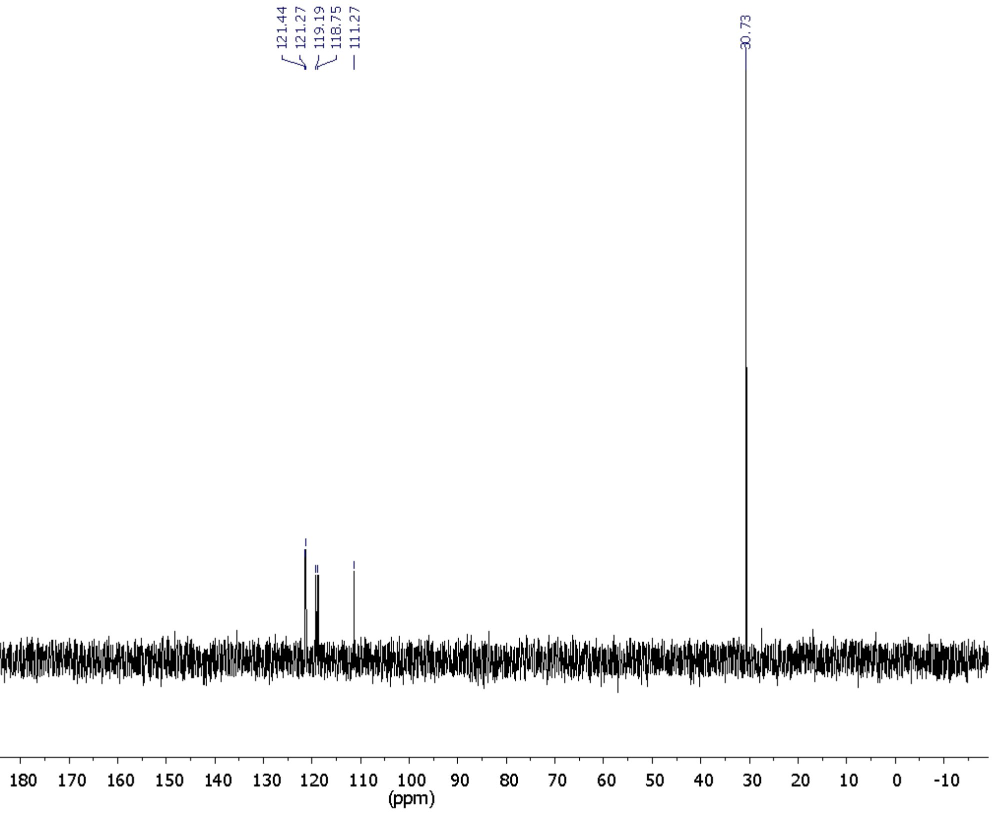


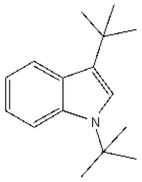

**8b**

$^{1}$H NMR

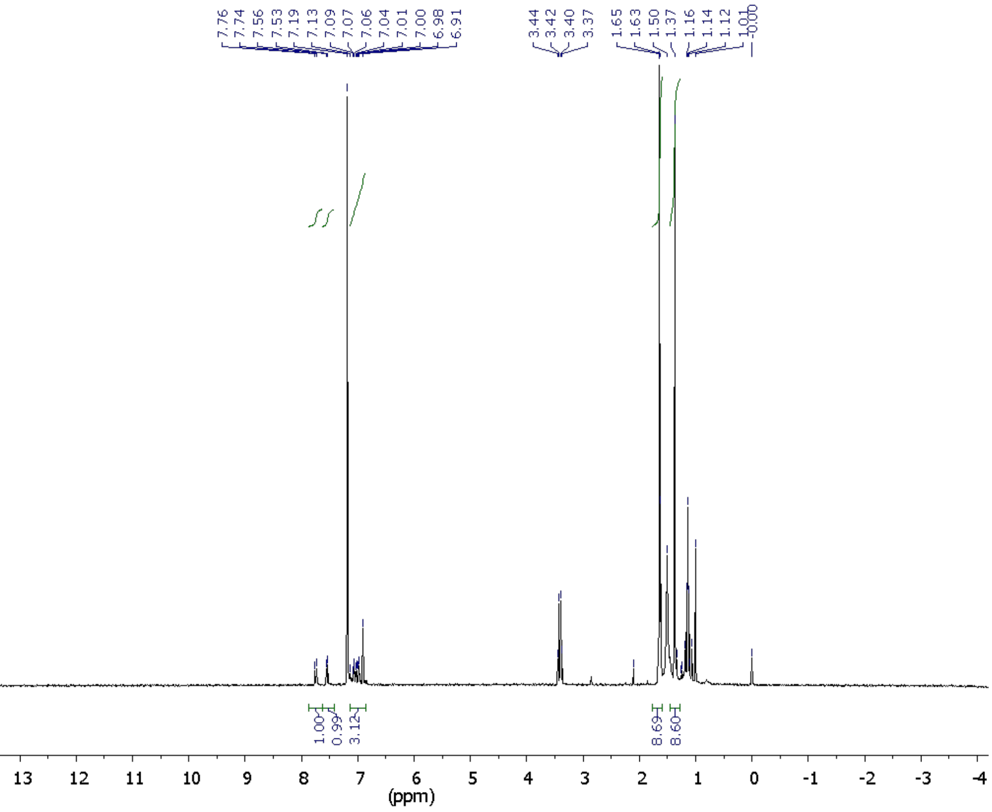

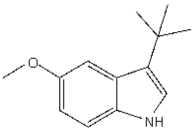

**9a**

$^{1}$H NMR

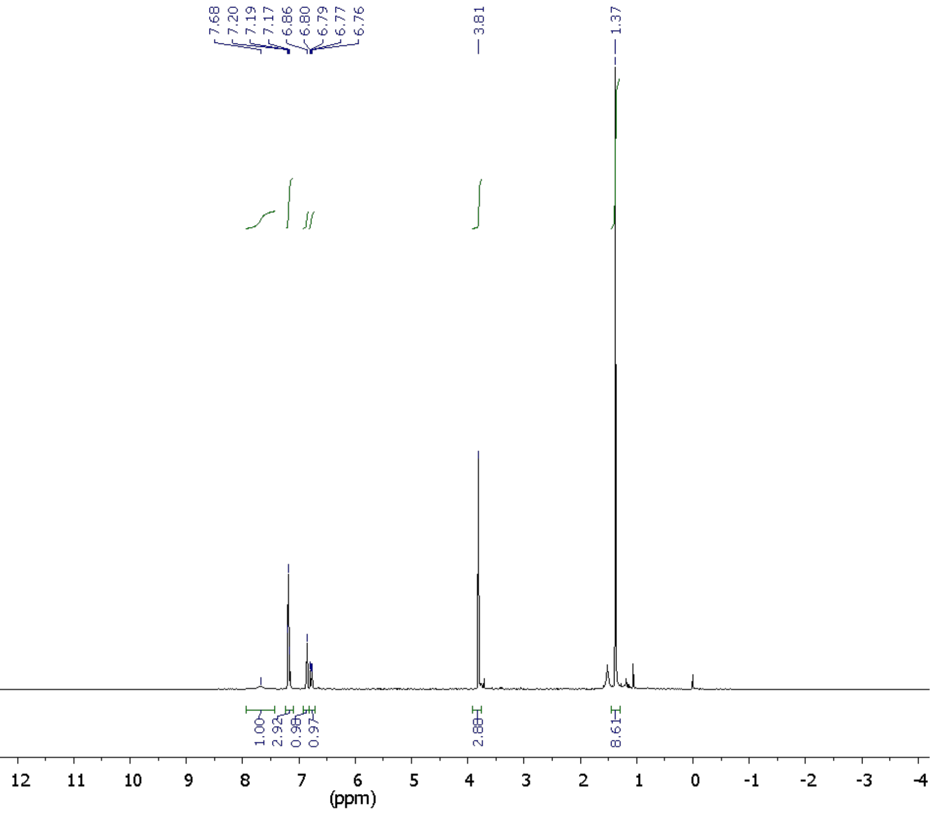


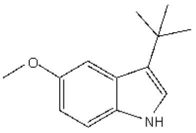

**9a**

$^{13}$C NMR

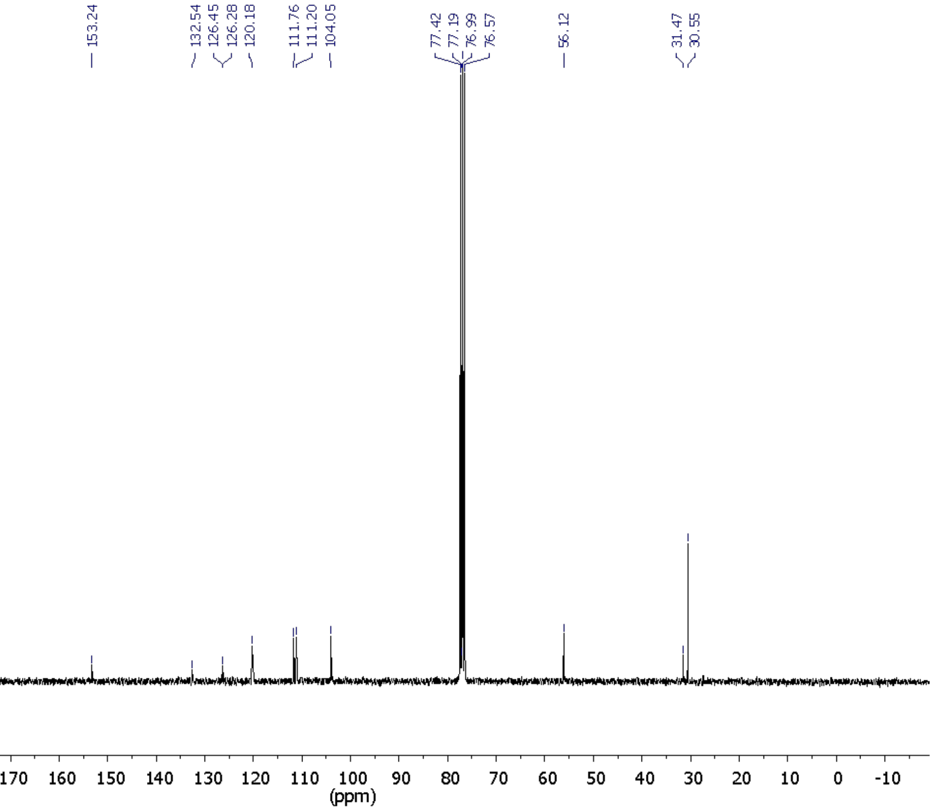

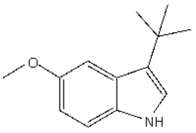

**9a**

DEPT

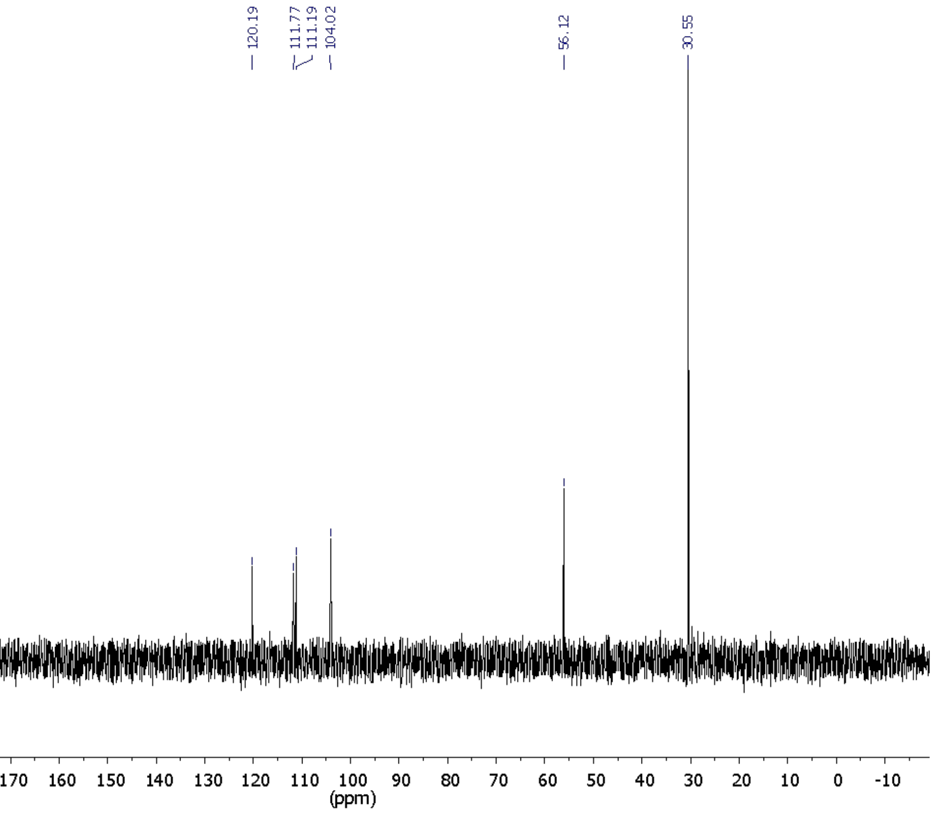


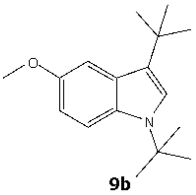

**9b**

$^{1}$H NMR

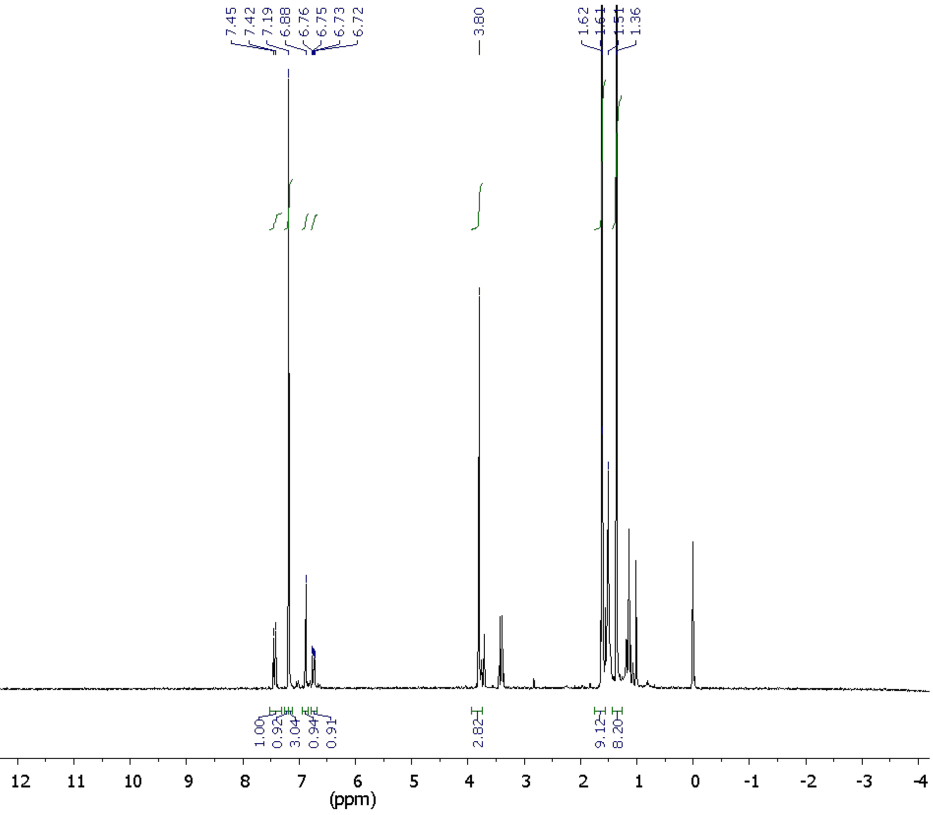

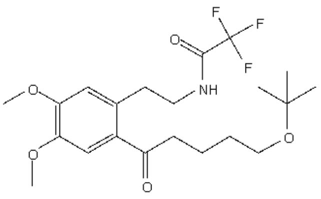


**11**

$^{1}$H NMR

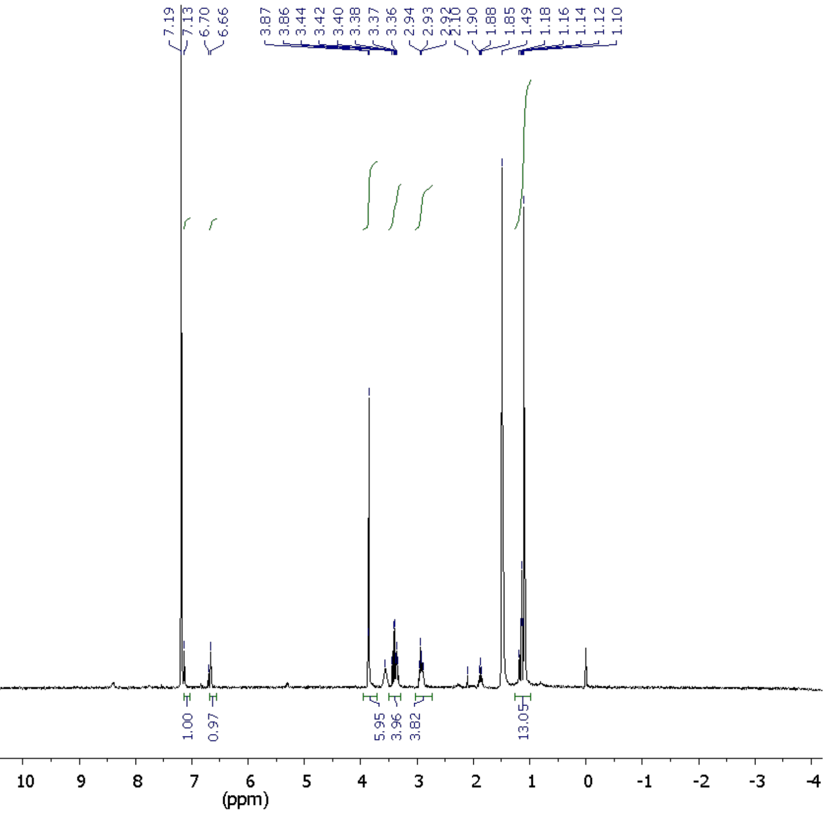


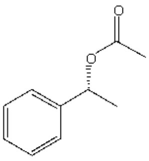


**13**

$^{1}$H NMR

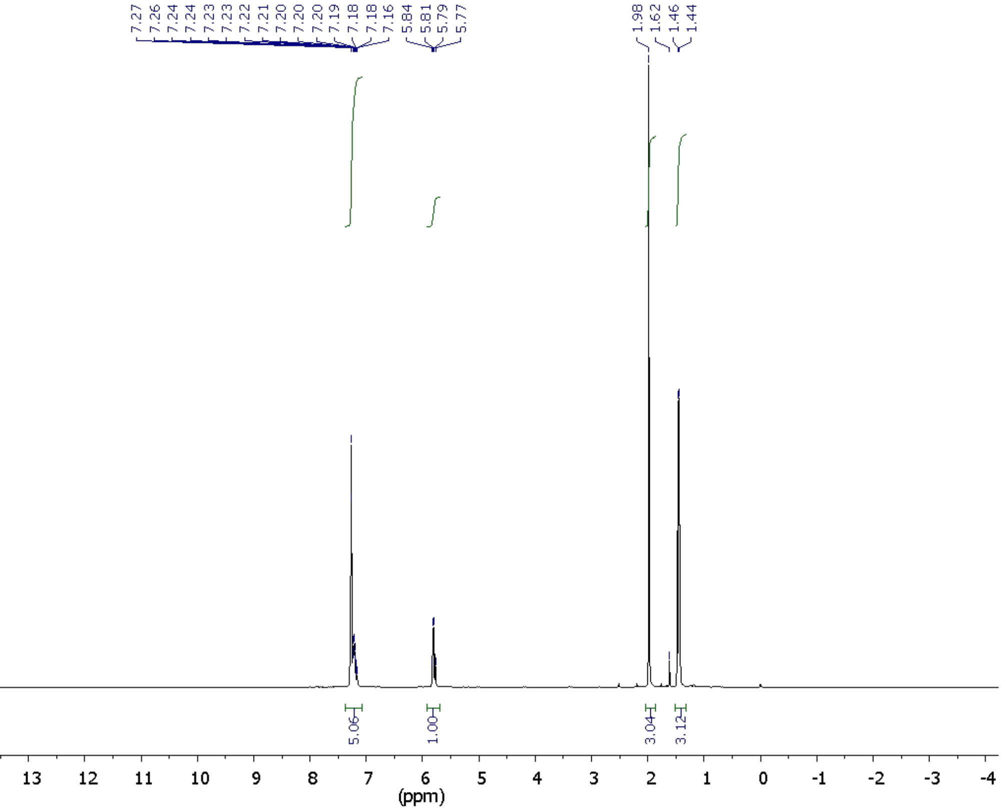

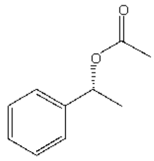

**13**

$^{13}$C NMR

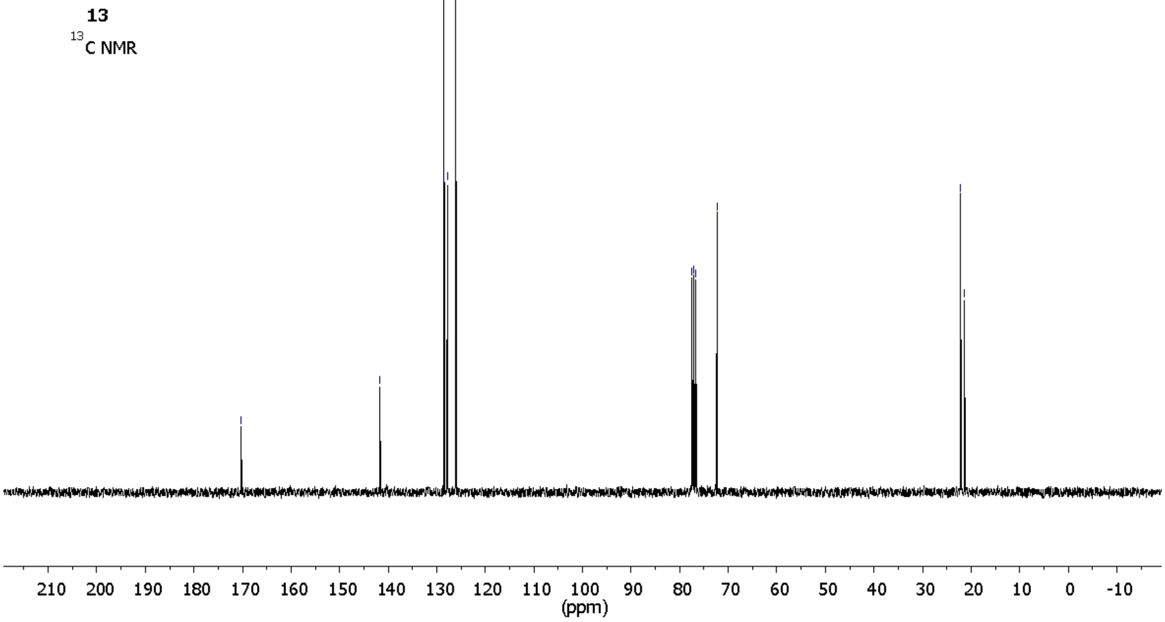


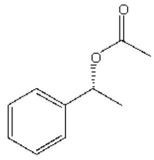

**13**

DEPT

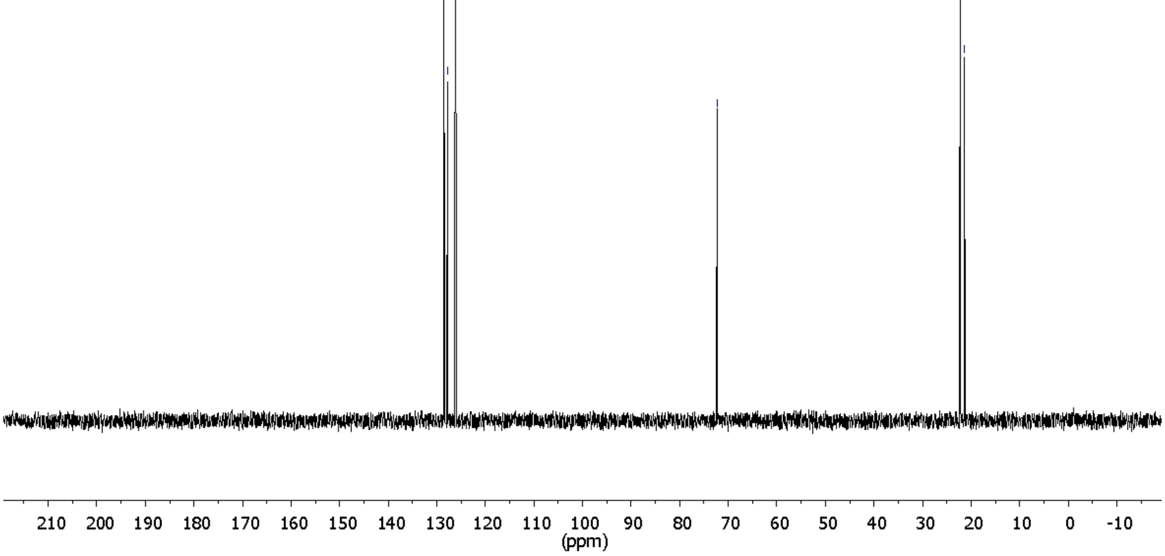

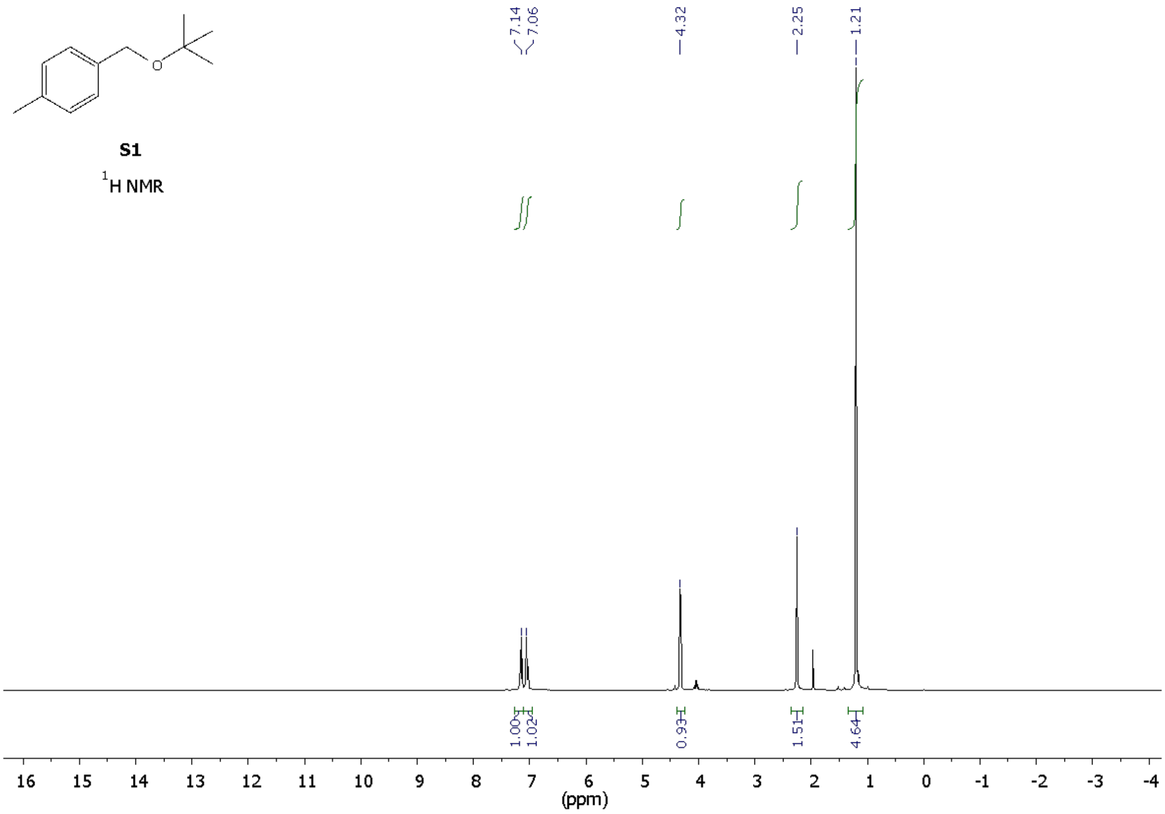
S1
1H NMR
7.14
7.06
4.32
2.25
1.21
1.00
1.02
0.93
1.51
4.64
16 15 14 13 12 11 10 9 8 7 6 5 4 3 2 1 0 -1 -2 -3 -4
(ppm)

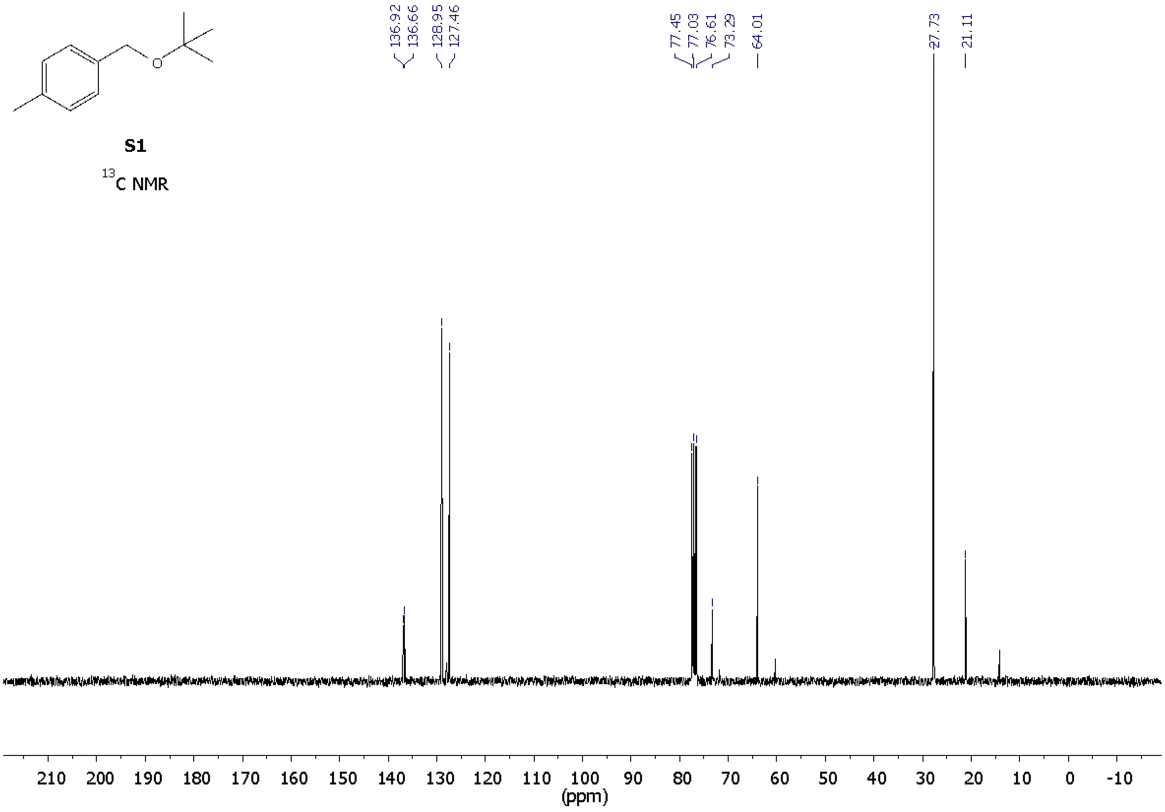
S1
13C NMR
136.92
136.66
128.95
127.46
77.45
77.03
76.61
73.29
64.01
27.73
21.11
210 200 190 180 170 160 150 140 130 120 110 100 90 80 70 60 50 40 30 20 10 0 -10
(ppm)

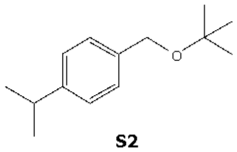

**S2**

$^{1}$H NMR

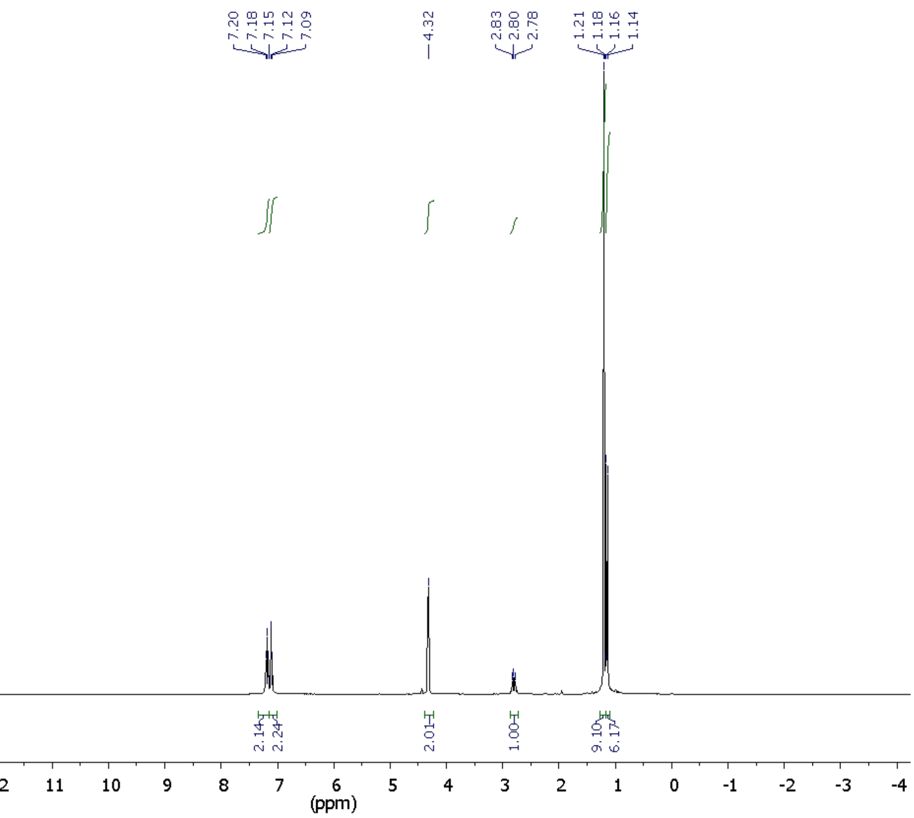


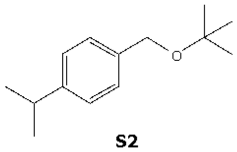

**S2**

$^{13}$C NMR

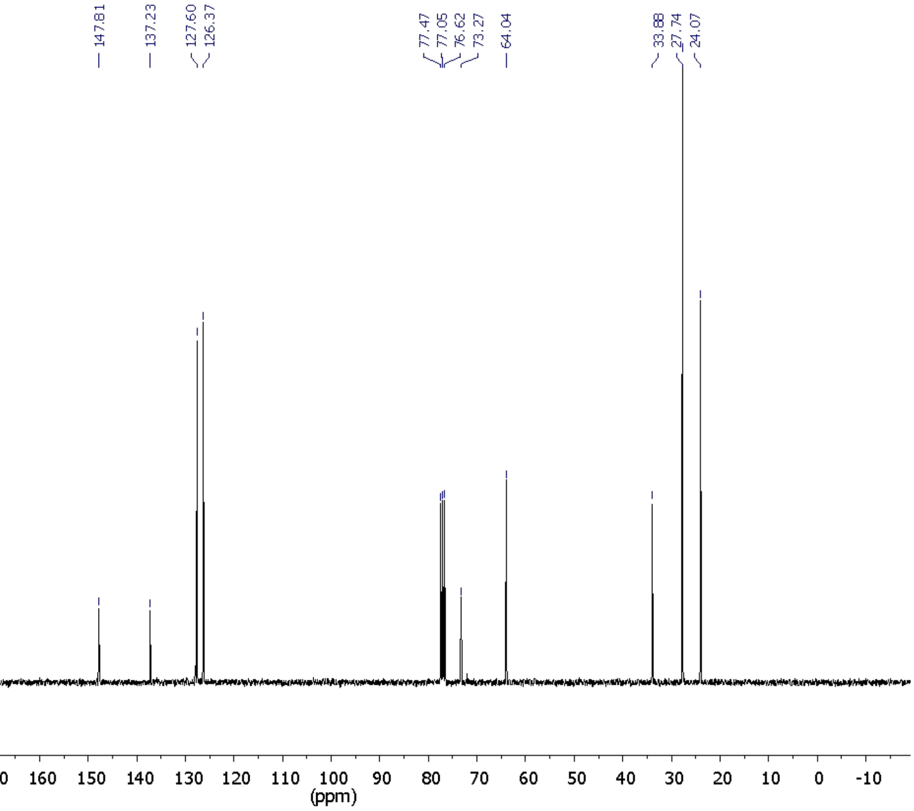

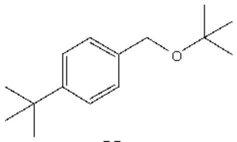

**S3**

$^{1}H$ NMR

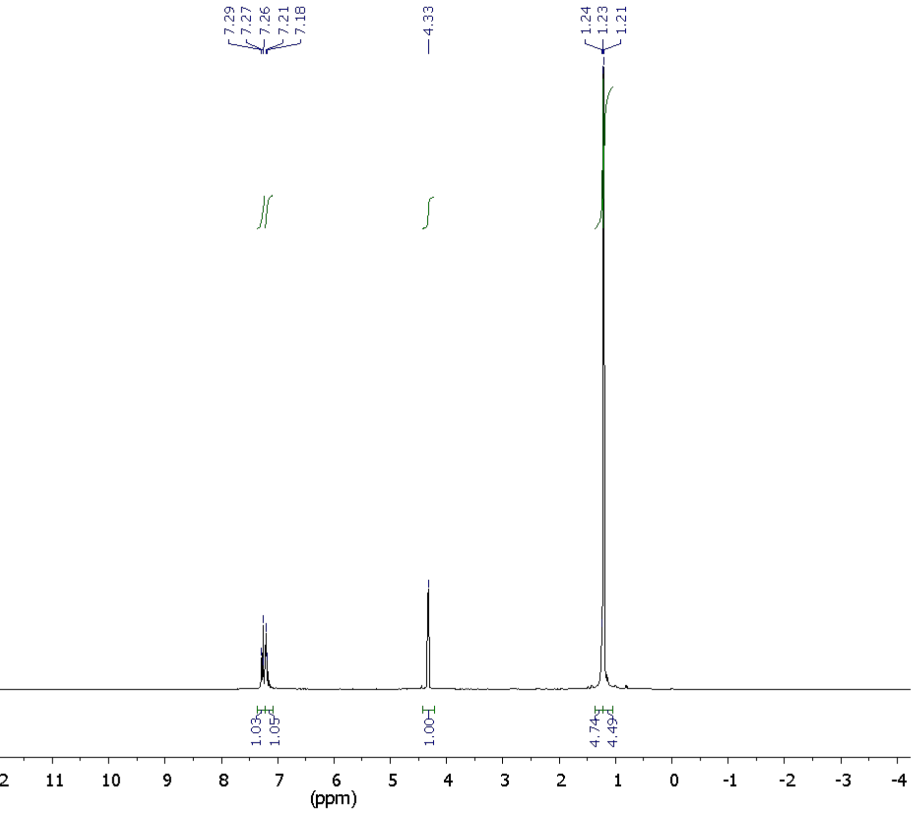


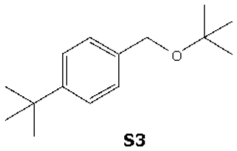

**S3**

$^{13}C$ NMR

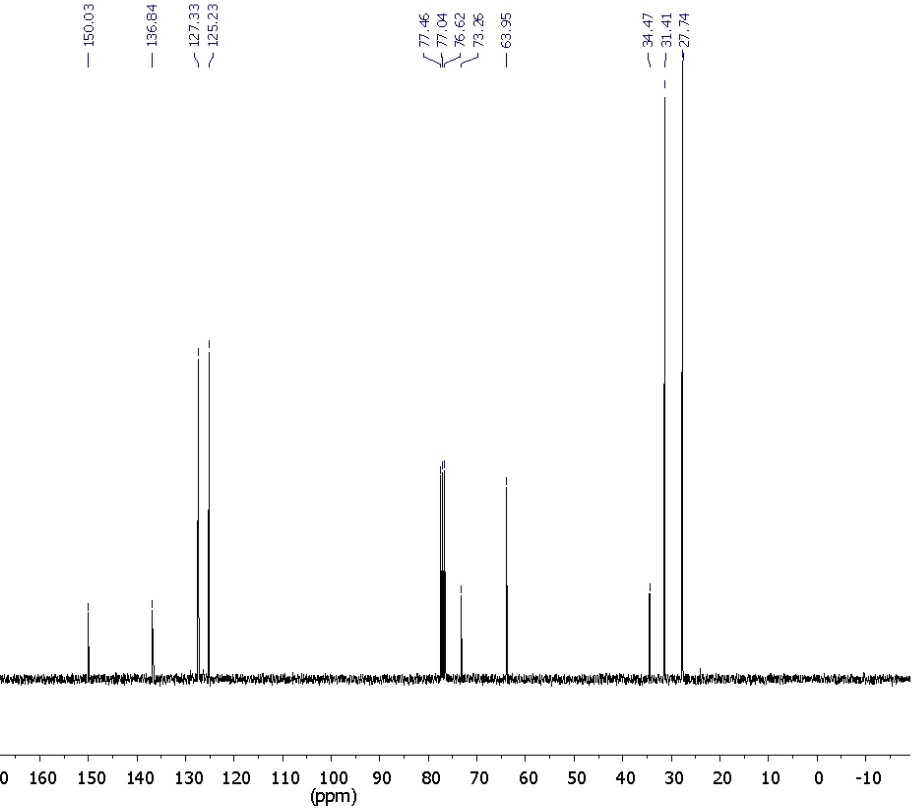

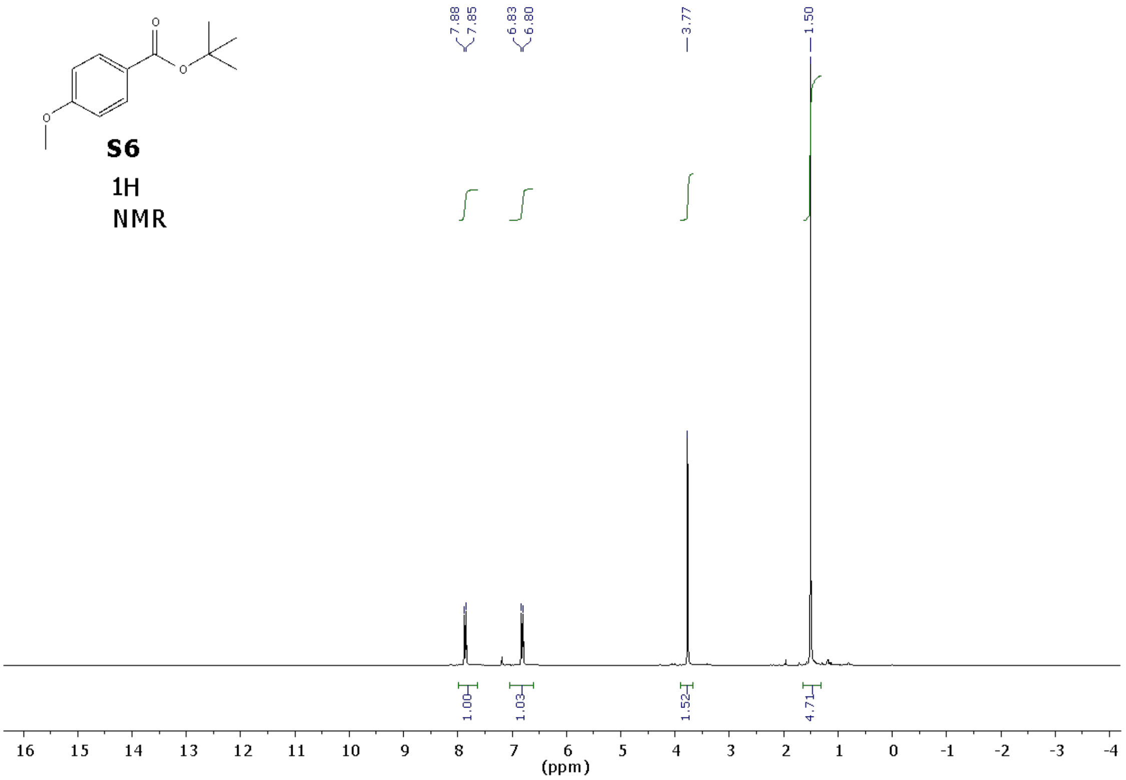
S6
1H
NMR
7.88
7.85
6.83
6.80
3.77
1.50
1.00
1.03
1.52
4.71
16 15 14 13 12 11 10 9 8 7 6 5 4 3 2 1 0 -1 -2 -3 -4
(ppm)

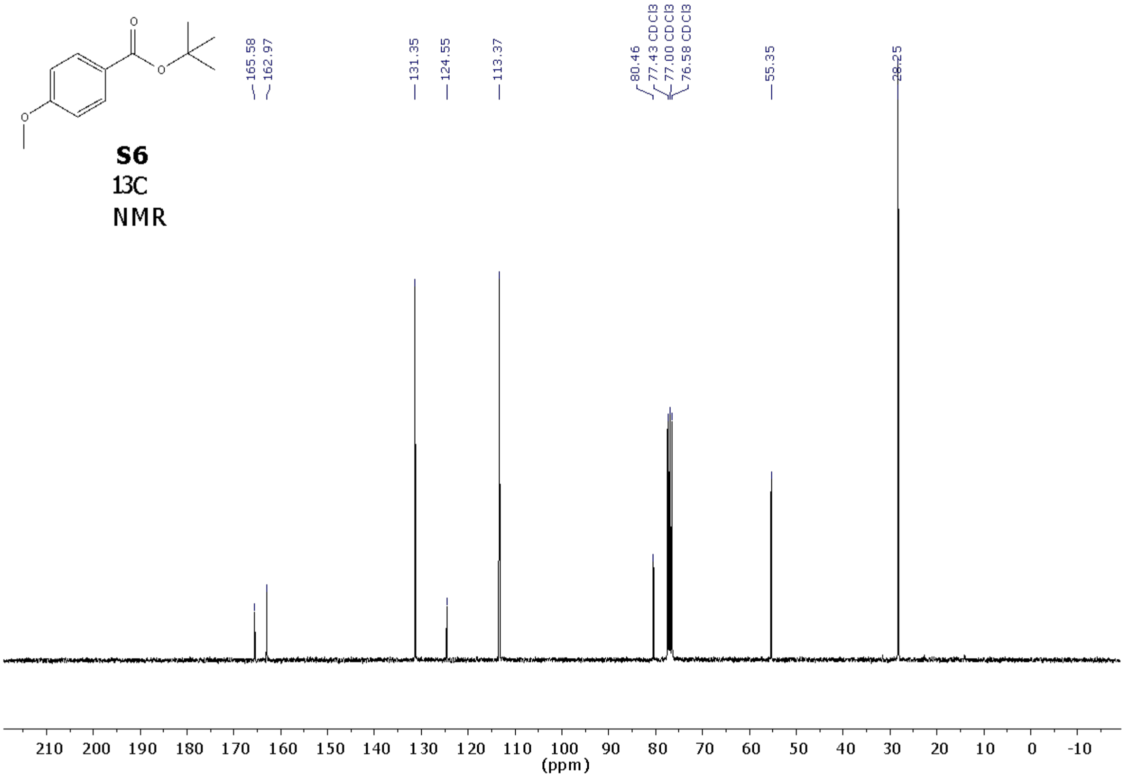
S6
13C
NMR
165.58
162.97
131.35
124.55
113.37
80.46
77.43 CDCl3
77.00 CDCl3
76.58 CDCl3
55.35
28.25
210 200 190 180 170 160 150 140 130 120 110 100 90 80 70 60 50 40 30 20 10 0 -10
(ppm)

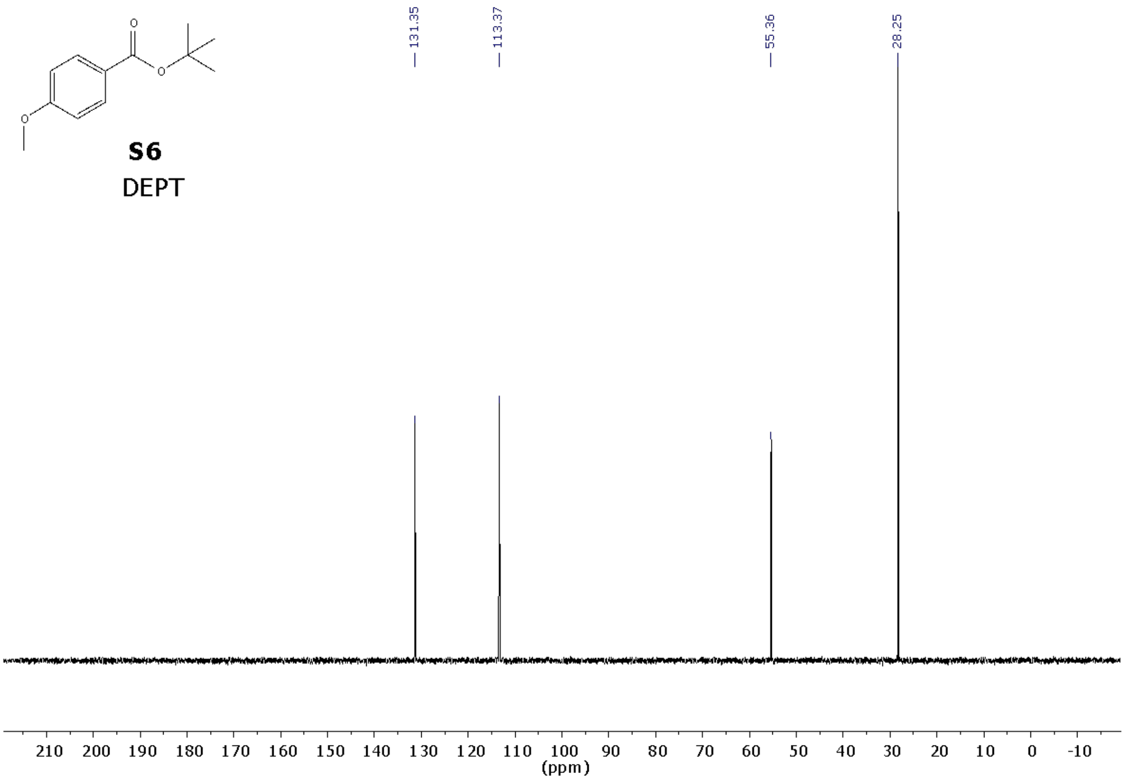
S6
DEPT
131.35
113.37
55.36
28.25
210 200 190 180 170 160 150 140 130 120 110 100 90 80 70 60 50 40 30 20 10 0 -10
(ppm)

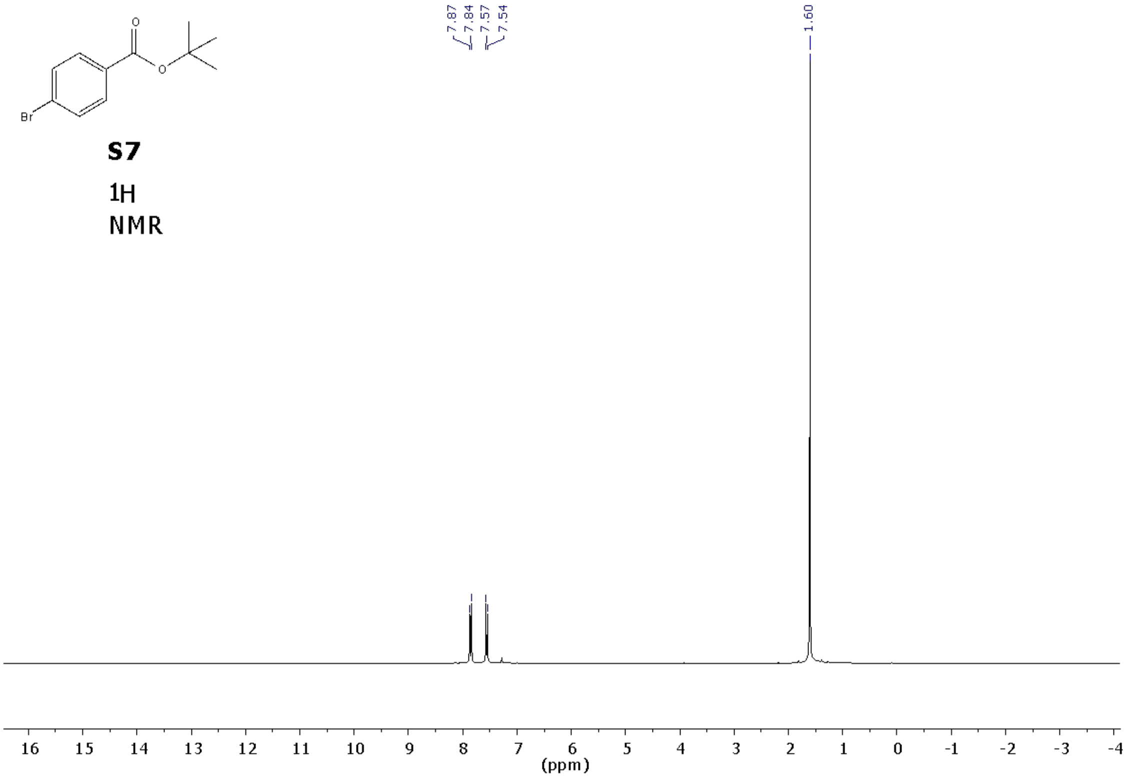
Br
S7
1H
NMR
7.87
7.84
7.57
7.54
1.60
16 15 14 13 12 11 10 9 8 7 6 5 4 3 2 1 0 -1 -2 -3 -4
(ppm)

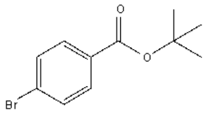


**S7**

$^{13}$C NMR

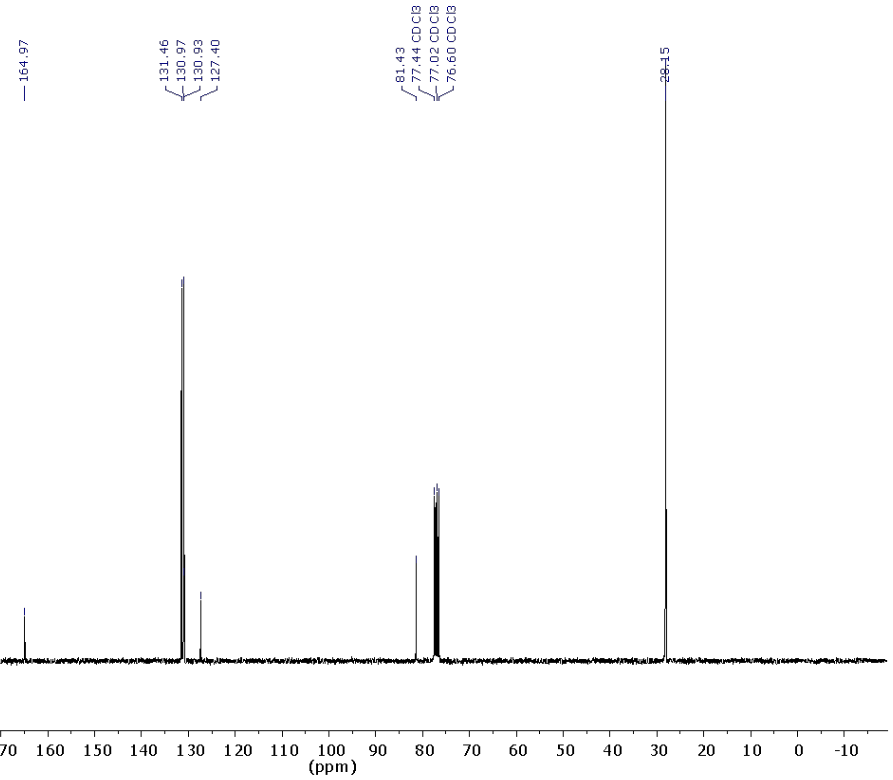


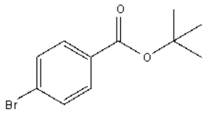


**S7**

DEPT

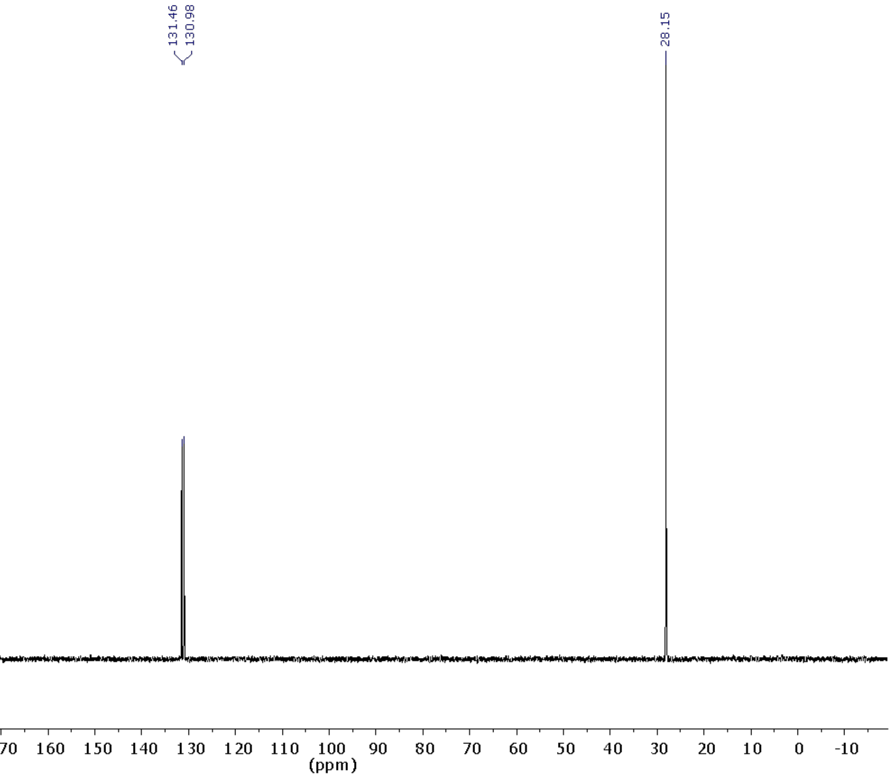

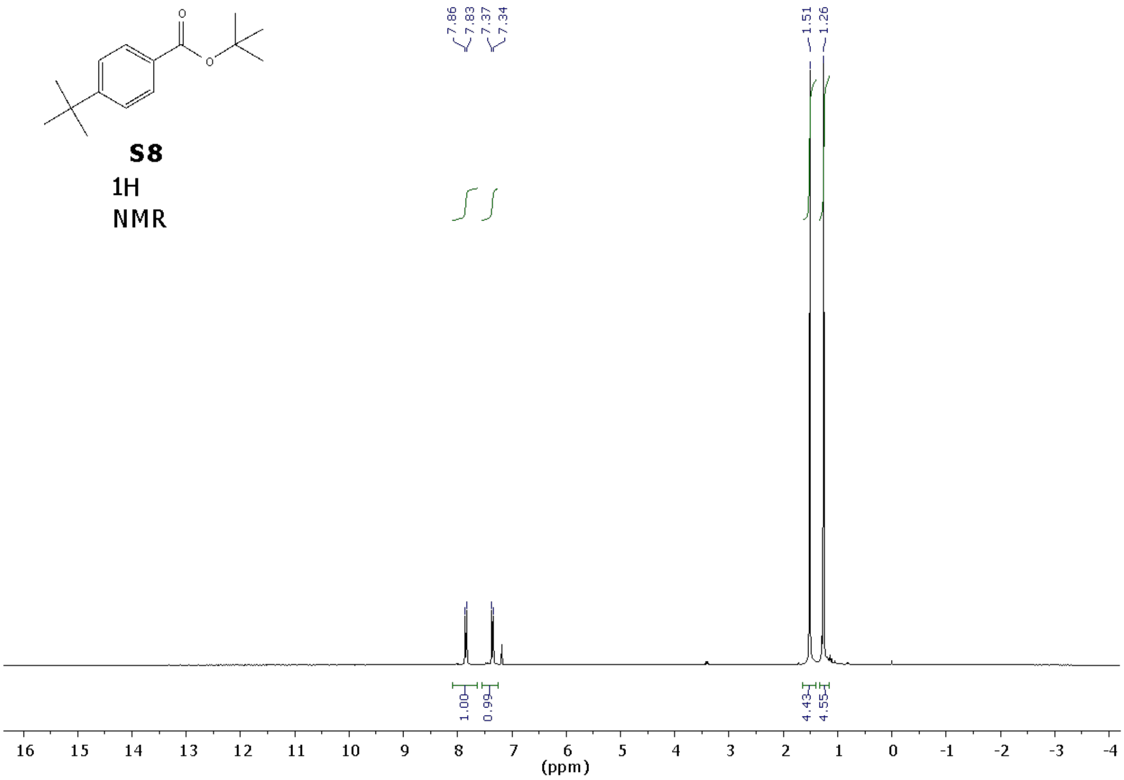
S8
1H
NMR
7.86
7.83
7.37
7.34
1.51
1.26
1.00
0.99
4.43
4.55
16 15 14 13 12 11 10 9 8 7 6 5 4 3 2 1 0 -1 -2 -3 -4
(ppm)

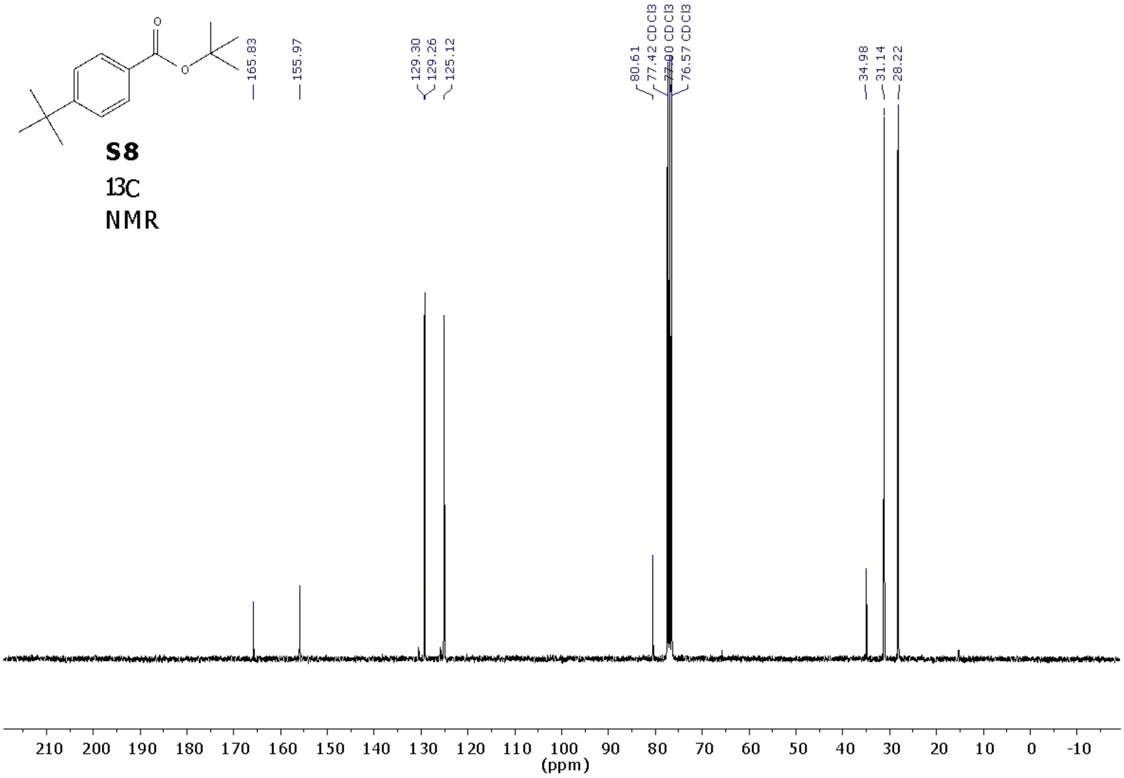
S8
13C
NMR
165.83
155.97
129.30
129.26
125.12
80.61
77.42 CDCl3
77.00 CDCl3
76.57 CDCl3
34.98
31.14
28.22
210 200 190 180 170 160 150 140 130 120 110 100 90 80 70 60 50 40 30 20 10 0 -10
(ppm)

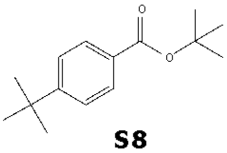

**S8**

DEPT

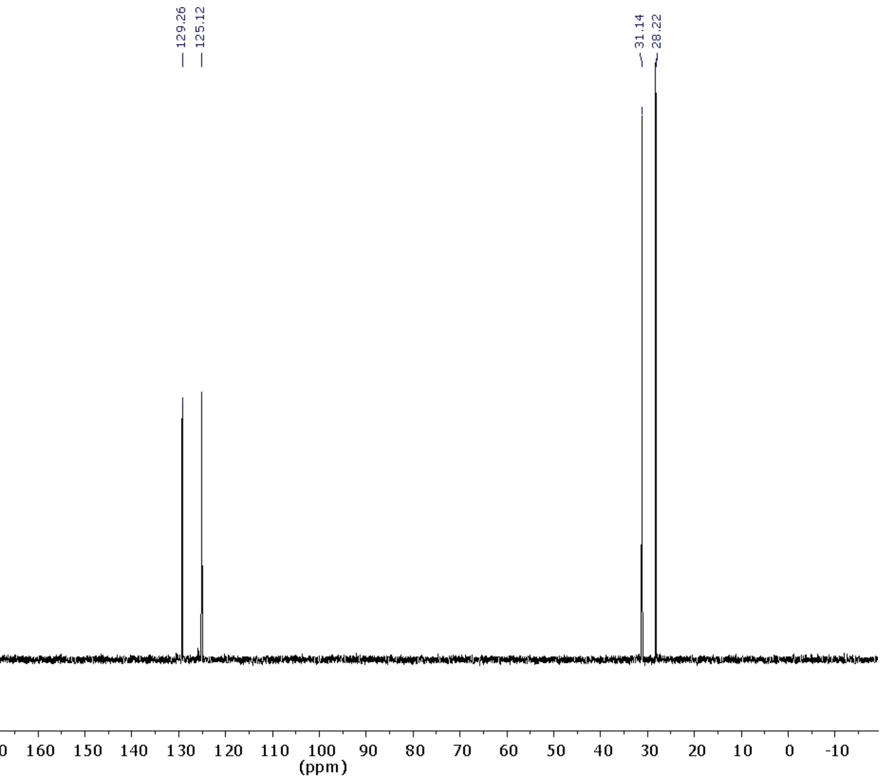


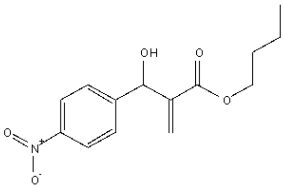

**S9**

1H NMR

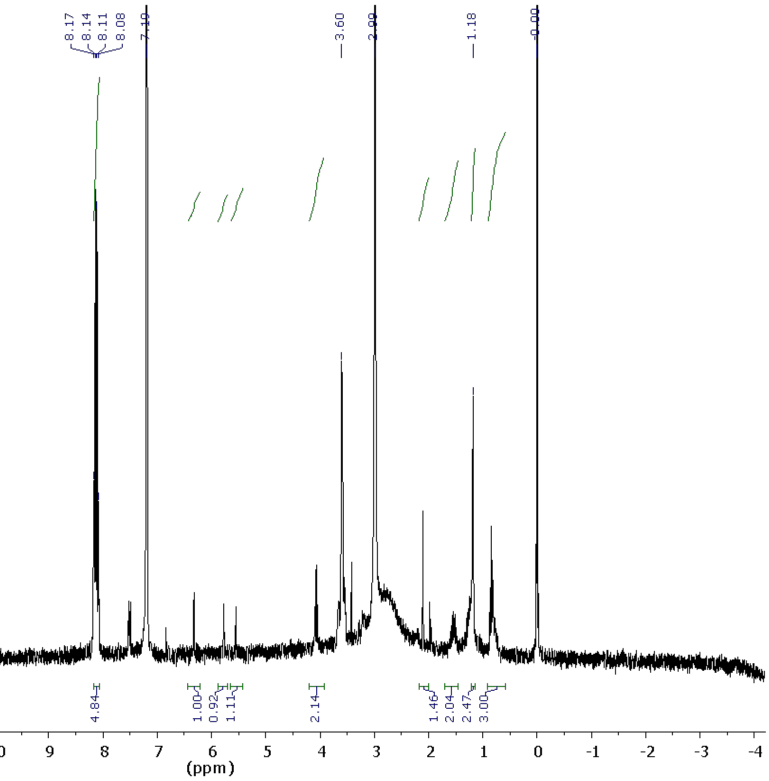

**Supplementary references.**


1. Crossley, S. W. M., Barabé, F. & Shenvi, R. A. Simple, Chemoselective, Catalytic Olefin Isomerization. *J. Am. Chem. Soc.* **136**, 16788-16791 (2014).
2. Tietze, L. F., Bachmann, J., Wichmann, J. & Burkhardt, O. *Synthesis*, **11**, 1185-1194 (1994).
3. Inazaki, T., Ito, T. & Tsuchimura, T. Method for producing vinyl ether compound. JP2012020954A (2012).
4. Bazin, J. F. Y. Approaches towards the synthesis of ebelactone A using silicon reagents. *Doctoral thesis* (University of Cambridge, Cambridge, United Kingdom, 1990).
5. Milata, V., Radl, S. & Voltrova, S. Science of Synthesis: Houben-Weyl Methods of Molecular Transformations, enol ethers. *Sci. Synth.* **32**, 589-756 (2008).
6. Motoyama, Y., Abe, M., Kamo, K., Kosako, Y. & Nagashima, H. Encapsulated molecular catalysts in polysiloxane gels: ruthenium cluster-catalyzed isomerization of alkenes. *Chem. Commun.* **42**, 5321-5323 (2008).
7. Nordmann, G. & Buchwald, S. L. A Domino Copper-Catalyzed C-O Coupling-Claisen Rearrangement Process. *J. Am. Chem. Soc.* **125**, 4978-4979 (2003).
8. Buchwald, S. L. et al. Copper-catalyzed formation of carbon-heteroatom and carbon-carbon bonds. *US Patent* WO2002085838A1 (2002).
9. Waldemar, A. & Encarnacion, L. A. A. Benzyl enol ethers via decarboxylation of α-(benzyloxy)-β-lactones derived from the lithium α(benzyloxy)-α-lithioacetate synthon. *Synthesis,* 388-390 (1979).
10. Liu, F. et al. Sulfated graphene as an efficient solid catalyst for acid-catalyzed liquid reactions. *J. Mater. Chem.* **22**, 5495-5502 (2012).
11. Sreedhar, B. et al. Acylation of alcohols and amines with carboxylic acids: a first report catalyzed by iron(III) oxide-containing activated carbon. *J. Mol. Catal. A-Chem.* **191**, 141-147 (2003).
12. Srivastava, P. et al. Synthesis, anti-inflammatory, analgesic, 5-lipoxygenase (5-LOX) inhibition activities, and molecular docking study of 7-substituted coumarin derivatives. *Bioorg. Chem.* **67**, 130-138 (2016).
13. Bo, L. et al. Hydration of Epoxides on [CoIII(salen)] Encapsulated in Silica-Based Nanoreactors. *Angew. Chem. Int. Ed.* **51**, 11517-11521 (2012).
14. Gao, Y., Ma, D., Wang, C., Guan, J. & Bao, X. Reduced graphene oxide as a catalyst for hydrogenation of nitrobenzene at room temperature. *Chem. Commun.* **47**, 2432-2434 (2011).
15. Mase, N. et al. Fine-Bubble-Based Strategy for the Palladium-Catalyzed Hydrogenation of Nitro Groups: Measurement of Ultrafine Bubbles in Organic Solvents. *Synlett* **28**, 2184-2188 (2017).
16. Primo, A., Neatu, F., Florea, M., Parvulescu, V. & García, H. Graphenes in the absence of metals as carbocatalysts for selective acetylene hydrogenation and alkene hydrogenation. *Nat. Commun.* **5**, 5291 (2014).
17. Cao, Y., Sui, Z., Zhu, Y., Zhou, X. & Chen, D. Selective Hydrogenation of Acetylene over Pd-In/Al2O3 Catalyst: Promotional Effect of Indium and Composition-Dependent Performance. *ACS Catalysis* **7**, 7835-7846 (2017).
18. Dreyer, D. R., Jia, H.-P. & Bielawski, C. W. Graphene Oxide: A Convenient Carbocatalyst for Facilitating Oxidation and Hydration Reactions. *Angew. Chem. Int. Ed.* **49**, 6813-6816 (2010).

19. Liu, J. et al. Room temperature selective oxidation of benzyl alcohol under base-free aqueous conditions on Pt/TiO2. *Cat. Commun.* **99**, 6-9 (2017).
20. Liu, W., Wang, H. & Li, C.-J. Metal-Free Markovnikov-Type Alkyne Hydration under Mild Conditions. *Org. Lett.* **18**, 2184-2187 (2016).
21. Dhakshinamoorthy, A., Primo, A., Concepcion, P., Alvaro, M. & Garcia H. Doped Graphene as a Metal-Free Carbocatalyst for the Selective Aerobic Oxidation of Benzylic Hydrocarbons, Cyclooctane and Styrene. *Chem. Eur. J.* **19**, 7547-7554 (2013).
